# Modification of the PM_2.5_- and extreme heat-mortality relationships by historical redlining: a case-crossover study in thirteen U.S. states

**DOI:** 10.1186/s12940-024-01055-5

**Published:** 2024-02-07

**Authors:** Edgar Castro, Abbie Liu, Yaguang Wei, Anna Kosheleva, Joel Schwartz

**Affiliations:** Harvard T.H. Chan School of Public Health, Boston, MA 02115 USA

**Keywords:** Air pollution, Extreme heat, Temperature, Environmental justice, Redlining, Effect modification

## Abstract

**Background:**

Redlining has been associated with worse health outcomes and various environmental disparities, separately, but little is known of the interaction between these two factors, if any. We aimed to estimate whether living in a historically-redlined area modifies the effects of exposures to ambient PM_2.5_ and extreme heat on mortality by non-external causes.

**Methods:**

We merged 8,884,733 adult mortality records from thirteen state departments of public health with scanned and georeferenced Home Owners Loan Corporation (HOLC) maps from the University of Richmond, daily average PM_2.5_ from a sophisticated prediction model on a 1-km grid, and daily temperature and vapor pressure from the Daymet V4 1-km grid. A case-crossover approach was used to assess modification of the effects of ambient PM_2.5_ and extreme heat exposures by redlining and control for all fixed and slow-varying factors by design. Multiple moving averages of PM_2.5_ and duration-aware analyses of extreme heat were used to assess the most vulnerable time windows.

**Results:**

We found significant statistical interactions between living in a redlined area and exposures to both ambient PM_2.5_ and extreme heat. Individuals who lived in redlined areas had an interaction odds ratio for mortality of 1.0093 (95% confidence interval [CI]: 1.0084, 1.0101) for each 10 µg m^−3^ increase in same-day ambient PM_2.5_ compared to individuals who did not live in redlined areas. For extreme heat, the interaction odds ratio was 1.0218 (95% CI 1.0031, 1.0408).

**Conclusions:**

Living in areas that were historically-redlined in the 1930’s increases the effects of exposures to both PM_2.5_ and extreme heat on mortality by non-external causes, suggesting that interventions to reduce environmental health disparities can be more effective by also considering the social context of an area and how to reduce disparities there. Further study is required to ascertain the specific pathways through which this effect modification operates and to develop interventions that can contribute to health equity for individuals living in these areas.

**Supplementary Information:**

The online version contains supplementary material available at 10.1186/s12940-024-01055-5.

## Introduction

Redlining is a discriminatory practice that arose out of the Great Depression with the formation of the Home Owners Loan Corporation (HOLC) in 1933. In the following years, the HOLC would produce its infamous security maps to guide decisions concerning who among those facing foreclosure could receive HOLC “rescue” mortgages to avoid default. Guided by racist housing practices and public opinion at the time, contractors responsible for creating these maps attributed excessive risk to neighborhoods that housed people of color [39]. The security maps they produced classified neighborhoods as being in one of four different risk categories: grade A, labeled “Best”; grade B, labeled “Still Desirable”; grade C; labeled “Still Declining”; and grade D, labeled “Hazardous”. Areas categorized as grade D were colored red on the maps, giving rise to the term “redlining”. In recent years, these maps have been digitized and georeferenced by researchers at the University of Richmond, providing researchers with the ability to investigate these inequities using GIS [36].

Though the HOLC’s security maps have long since been abolished, their effects continue to persist today, especially in neighborhoods that have been redlined. Researchers have hypothesized various mechanisms through which the HOLC’s security maps continue to influence the present-day makeup of cities, such as through the persistence of differential patterns of housing stock between neighborhoods that were attributed different levels of risk [2]. Studies have found that neighborhoods that were attributed higher risk, such as those that were redlined, were less likely targets for the construction of new homes on account of their residents’ decreased ability to receive credit; today, these same areas are more likely to have older housing stock, fewer housing units, higher proportions of multifamily homes with rented units, and lower housing values [1, 2, 26]. Further downstream of these effects, the physical environment of the neighborhood as a whole can then be negatively impacted by the decreased material, social, and political capital of their residents.

Accordingly, studies have outlined how individuals living in previously-redlined areas experience lower exposure to green space [34]; higher temperatures [20, 46],worse air pollution [27], and worse health outcomes such as lower life expectancy, higher risk of preterm birth, and worse cardiovascular health, among others [21, 25, 31–33] nearly a century after the establishment of the HOLC in 1933 and many decades after its abolishment. There has also been recent research detailing the persistence of the effects of past persecution and discrimination on present-day neighborhoods in other contexts, with even longer time periods, giving additional credence to such findings [14].

Separately, there is also extensive literature on the synergistic effects of air pollution and chronic psychosocial stress, such as that arising from exposure to violence [8, 10]. One hypothesized physiologic mechanism is the modulation of immune and inflammatory pathways through allostatic load caused by chronic stress, which increases susceptibility to air pollution [7, 9]. However, no previous studies have investigated the effect modification, if any, of the effect of exposures to PM_2.5_ or extreme heat on health outcomes by redlining. One recent study in Texas showed that historically-redlined neighborhoods have both higher land surface temperatures and higher risks of heat-related illnesses, but it’s unclear how much of this disparity is due to the increased temperature and how much is due to increased vulnerability [28].

**Fig. 1:**
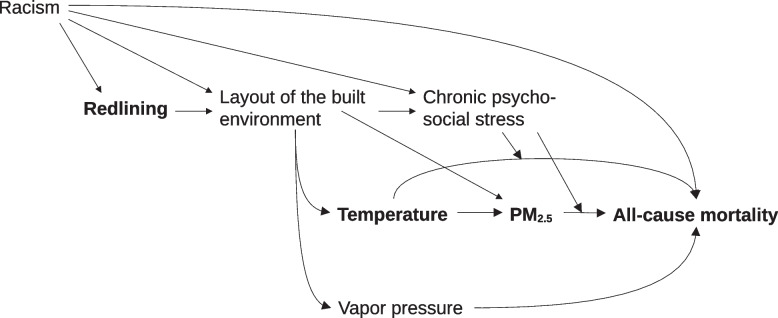
Proposed DAG illustrating the modification of the PM_2.5_- and temperature-all cause mortality relationships by redlining. The exposures of interest are presented in boldface and underlined, and the relationships of interest are indicated with thicker lines

In this study, we leverage extensive, geocoded, case-level mortality records from thirteen states; digitized and georeferenced HOLC maps; and sophisticated, high-resolution ambient PM_2.5_ and meteorological models to investigate modification of the PM_2.5_- and extreme heat-mortality relationships by living in a redlined area. Figure 1 encodes our assumptions of the causal relations pertinent to the present study. Based on literature, we hypothesize that the way in which redlining affects the PM_2.5_-mortality and extreme heat-mortality relationships is through chronic psychosocial stress driven both directly and indirectly by the layout of the built environment, involving for example reduced accessed to services, social and material deprivation, exposure to violence, and other hazards. Notably, patterns of land use resulting from redlining open backdoor paths through which the PM_2.5_- and extreme heat-all cause mortality relationships, respectively, as well as their interactions, become confounded. Historical racism additionally confounds the redlining-mortality relationship. All of these confounders apart from humidity are time-invariant or very slow-varying and can easily be adjusted for with time series methods. However, although these relatively fixed factors cannot be confounders in a time series analysis, they can be effect modifiers and increase susceptibility. Other downstream impacts of redlining that could potentially impact effect modification by redlining, such as lower present-day SES, occurred post-1937 and so cannot be confounders but are instead mediators.

The results from this study can be used to help justify future studies and inform future interventions aimed at reducing the health disparities caused by PM_2.5_ and extreme heat exposure in disadvantaged neighborhoods and additionally help to elucidate the degree to which historical discrimination influences present-day physiological responses to both exposures.

## Methods

### Data sources

Individual-level statewide mortality data and additional characteristics including age, race, geocodes, and sex were obtained from the California, Florida, Georgia, Illinois, Indiana, Kansas, Massachusetts, Michigan, Missouri, New Hampshire, New Jersey, Ohio, and Texas departments of public health. Records had listed either the individual’s home address (California, Florida, Illinois, Indiana, Kansas, Massachusetts, Missouri, New Hampshire, Ohio, and Texas only) or the encompassing Census block, block group, tract, or county geographic identifier (GEOID). Prior to joining other variables, all home locations were standardized to Census block groups. Home locations that were reported as addresses were geocoded to coordinates using ArcGIS Pro and then spatially joined via point-in-polygon to the encompassing Census block group using the *sf* package to obtain the Census block group GEOID [16, 37]. In other words, each death was assigned the GEOID of the block group that it was contained within. Records where the home address was reported as a block group GEOID were left as-is. Records with block-level GEOIDs had their GEOIDs, which are 15 characters long, truncated at the 12th character to obtain the block group GEOIDs [44]. Records from coarser geographies (i.e. tract- and county-level) were dropped. Once all geographic information had been standardized to block groups, these GEOIDs served as the basis for joining the other data.

Daily PM_2.5_ predictions from January 1st, 2001 to December 31st, 2016 were generated nationwide on a 1-km grid using data from air quality monitors, remotely-sensed satellite data, outputs from two chemical transport models, meteorological data, and land-use data using machine learning models described elsewhere [12]. Briefly, air pollution measurements and predictors from various sources were used to train a geographically-weighted ensemble of machine learners which then were used to predict daily air pollution within 1-km grid cells covering the contiguous USA. The overall hybrid model has yielded strong performance, with ten-fold cross-validation R^2^ values of 0.77–0.92 depending on the region and an R^2^ value of 0.86 overall. For each Census block, daily PM_2.5_ values were assigned by taking the average of predictions from encompassed grid cell centroids. For Census blocks that did not encompass any grid cell centroids, data was assigned by linking those blocks’ centroids to the nearest grid cell centroid by Cartesian distance [15]. Block group-level PM_2.5_ predictions were then calculated as the population-weighted average of these block-level assignments.

Daily minimum and maximum temperature and vapor pressure were obtained from the Daymet V4 meteorological model on a 1-km grid [43]. Minimum and maximum temperatures and vapor pressure in each Census block group were then assessed as the areal-weighted average of values from encompassed grid cells using the *exactextract* program [4], and the daily mean temperature was assessed by averaging these two values. To derive measures of extreme heat, we first calculated various percentiles of minimum temperature in each block group in each year. For our main analysis, we considered the 95th percentile. These percentiles were then used as lower bound cutoffs in our determination of what constitutes extreme heat and indicator variables were created for days where the minimum temperatures were higher than these cutoffs. In other words, if the minimum temperature on a certain day met or exceeded the 95th percentile of minimum temperature in that block group in that year, then that day was marked as an extreme heat day.

This approach to identifying days of extreme heat affords two advantages. Firstly, we account for regional and temporal adaptation to rising temperatures by using spatiotemporally local definitions of “extreme”. Additionally, by focusing on the minimum temperature, we guarantee that the temperature on a given extreme heat day was at least as hot as the cutoff for that day. After creating extreme heat indicators, we categorized all contiguous sequences of two or more days of extreme heat as heat waves and, among these days, calculated the heat wave day as the number of days from the start of the heat wave.

**Fig. 2:**
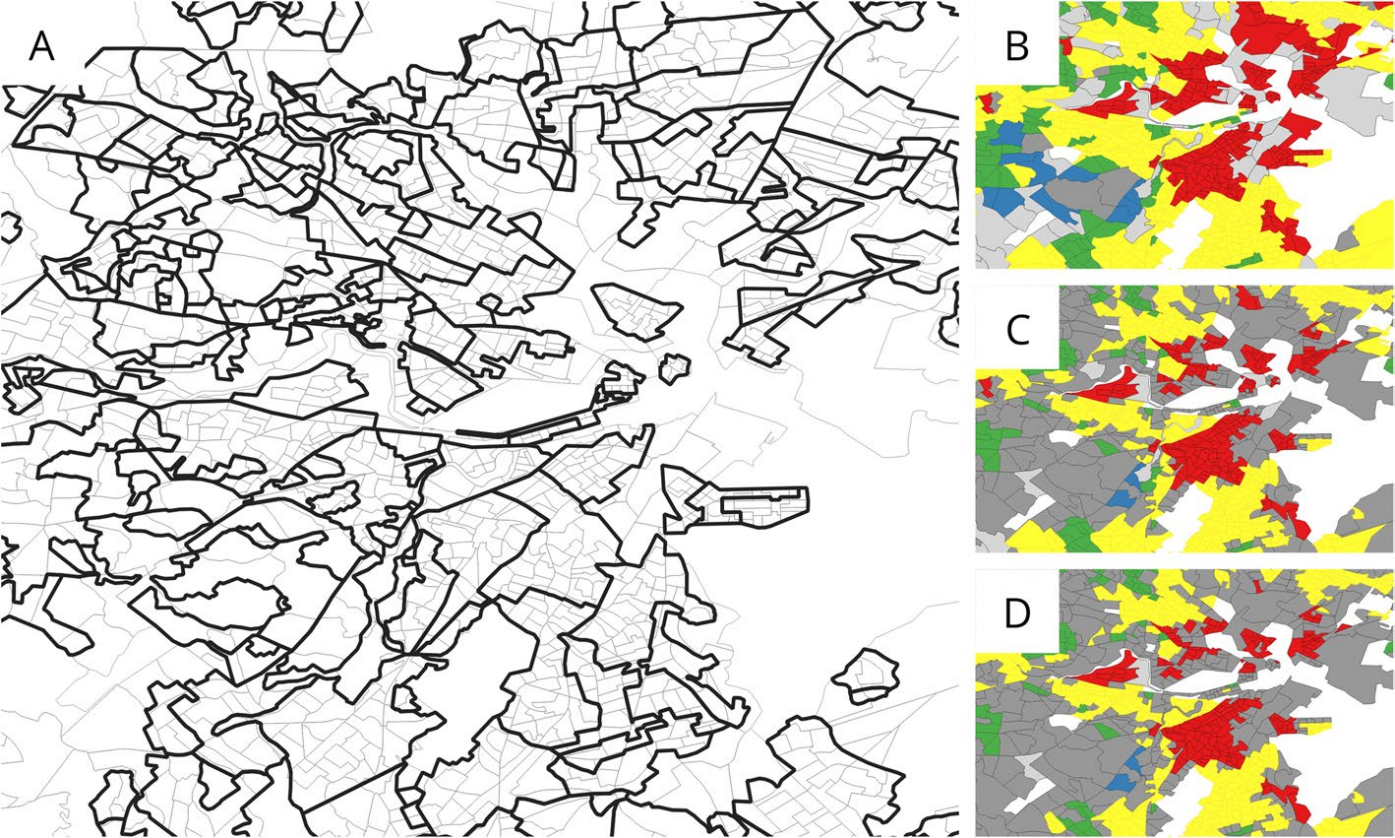
Figure demonstrating (**A**) the size comparison and spatial misalignment between HOLC-graded areas (outlined in black) and Census block groups (2010, outlined in gray), and (**B**-**D**) block groups being graded according to population cutoffs of 50%, 90%, and 99%, respectively. Blue areas are grade A, green areas are grade B, yellow areas are grade C, red areas are grade D (“redlined”), dark grey areas are marked as ambiguous (i.e. no grade bin exceeded the threshold), and light grey areas are marked as unclassified (i.e. the unclassified bin exceeded the threshold). White areas are either bodies of water or block groups that did not intersect with HOLC-graded areas

Digitized and georeferenced Home Owners Loan Corporation (HOLC) maps were obtained from the Mapping Inequality project at the University of Richmond [36]. Because HOLC geographies do not perfectly align with Census block groups, we assigned block groups to HOLC grades based on the proportion of encompassed population at the block level, which is the finest resolution for which national population data is available from the Census. First, the population of each Census block was binned into the grade of the encompassing HOLC area, if any, via point-in-polygon spatial join on block centroids. Block groups were then assigned a grade by selecting the bin that contained greater than some percentage threshold of the block group’s total population, if any (Fig. 2). Unlike other methods that assign HOLC grades based on the proportion of intersecting area, this method does not make an assumption of uniform population density across block groups and can more accurately capture what historically-assigned HOLC grade encompasses the most people in present day. In our main analysis, we used a 90% population threshold. Lower thresholds make the apportionment of HOLC grades to present-day block groups more ambiguous, but higher thresholds exclude more block groups due to no HOLC grade bin sizes meeting the population threshold.

Use of mortality records was permitted by the California, Florida, Georgia, Illinois, Indiana, Kansas, Massachusetts, Michigan, Missouri, New Hampshire, New Jersey, Ohio, and Texas departments of public health and approved by the IRBs of each. This study was reviewed by the IRB of the Harvard School of Public Health and classified as not human research.

### Statistical analysis

Prior to statistical analyses, deaths were restricted to only those from internal causes (ICD codes A00-R99) involving individuals 18 years of age or older. A case-crossover analysis was used to control for confounding by all fixed or slow-varying factors by design, such as sex, race and ethnicity, smoking history, etc. Specifically, for each case, we sampled control days bidirectionally from the days within the same month of the case that were on the same day of the week as the case to control for all factors that are either fixed on the scale of a month or vary on a cyclic weekly basis. This includes slowly varying individual or neighborhood predictors. Time-varying exposures were then reassessed for each case on each of these control days. Since each individual is compared to themselves at a different point in time, all fixed and cyclic weekly confounding factors are controlled for by design. Additionally, since we sample controls from days occurring both before and after the case, we are able to control for bias arising from time trends in the exposures [35]. In exchange for controlling for these time trends by design by sampling bidirectionally, a small bias is induced by sampling from controls post-death, but the size of this bias is very small due to the low daily risk of death at baseline [29].

In our primary analysis, we fit two sets of conditional logistic regressions within strata of each individual. For the redlining-PM_2.5_ interaction, we fit the following:

$$clogit\left(Pr\left(\mathrm{death}\right)\right)=\beta_0+\beta_1{\mathrm{PM}}_{2.5}+\left(\sum\limits_{i=2}^5\beta_1\right)ns\left(\mathrm{TMEAN},\;4\right)+\left(\sum\limits_{j=6}^9\beta_j\right)ns\left(\mathrm{VP},4\right)+\beta_{10}redlined\times{\mathrm{PM}}_{2.5}$$

where *redlined* is an indicator variable for HOLC grade D, PM_2.5_ is either the ambient concentration of PM_2.5_ on the day of the death or a moving average up to 4 days before the death (i.e. the 5-day moving average), *TMEAN* and *VP* are mean temperature and vapor pressure, respectively, and *β*_*10*_ is the primary estimand of interest. For the redlining-extreme heat interaction, we instead fit the following:

$$clogit\left(Pr\left(\mathrm{death}\right)\right)=\beta_0+\beta_1\mathrm{extreme}+\left(\sum\limits_{i=2}^5\beta_i\right)ns\left(\mathrm{VP},4\right)+\beta_6redlined\times\mathrm{extreme}$$

where *extreme* is an indicator variable either for extreme heat or for the 1st, 2nd, 3rd, or 4th day of extreme heat occurring on the day of the death and *β*_*6*_ is the primary estimand of interest.

To investigate the robustness of our findings, we carried out several sensitivity analyses. Firstly, we alternatively considered different block group-level HOLC grade apportionments based on cutoffs of 50% and 99% of the block group-level population. Secondly, we also alternatively considered 85th and 99th percentile cutoffs of minimum temperature in our definition of extreme heat. Thirdly, we carried out subgroup analyses within Black individuals, Black neighborhoods, White individuals, and White neighborhoods, where Black and White neighborhoods were defined as block groups where the proportion of residents that identified as Black or White was 50% or greater to investigate variation in vulnerability across individual- and area-level demographics. Lastly, we carried out subgroup analyses by year and by state to investigate spatial and temporal variation in vulnerability.

Results are presented for each 10 µg m^−3^ change in PM_2.5_ concentration. R version 4.1.0 was used for all analyses [38].

## Results

**Fig. 3:**
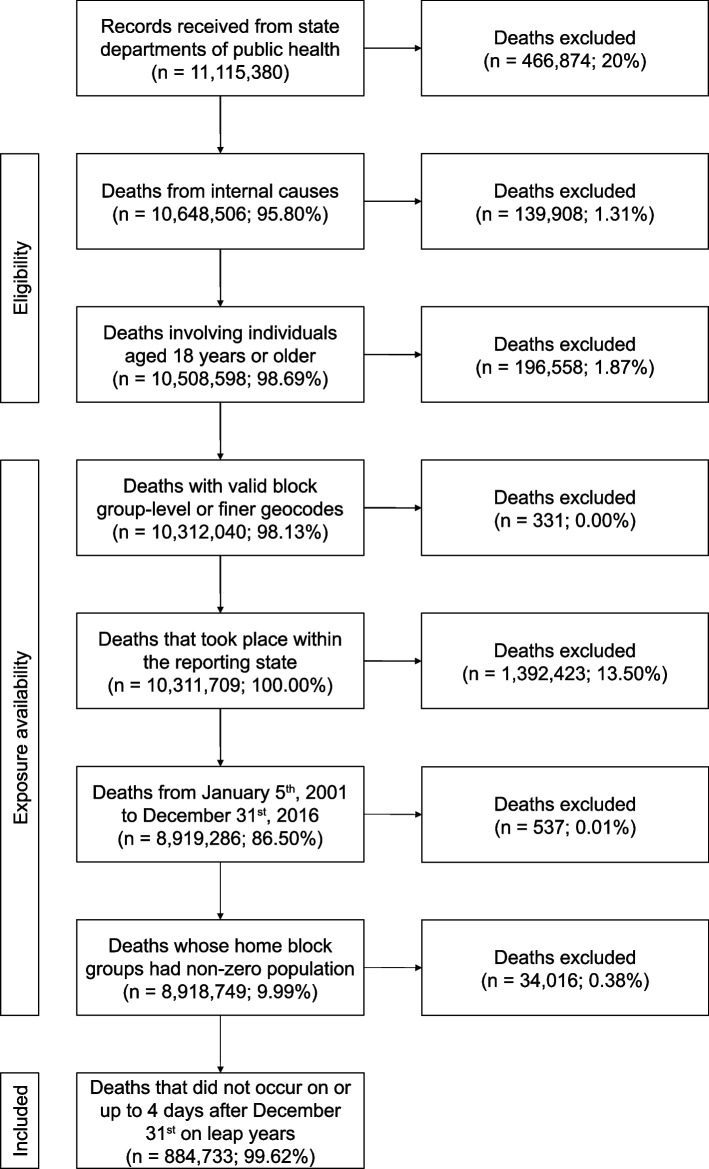
Subject restriction flowchart

We obtained 11,115,380 mortality records from the twelve state departments of public health. From these records, we sequentially excluded 466,874 deaths involving external causes; 139,908 deaths involving individuals younger than 18 years old; 196,558 deaths with geocodes that were missing or coarser than block group-level; 331 deaths involving individuals whose home locations were outside of the state that reported their death; 1,392,423 deaths before January 5th, 2001 or after December 31st, 2016 and 537 deaths whose home block groups had a population of zero according to the preceding Decennial Census (for which 4-day moving averages of population-weighted PM_2.5_ could not be calculated); and 34,016 deaths with lag days from 0 to 4 that included December 31st on leap years (for which Daymet predictions are not available; Fig. 3). This resulted in a final data set of 8,884,733 records.

Baseline characteristics are shown in Table 1. In the full data set, cases mostly involved individuals who were white (85.72%), had high school education alone (41.15%), and lived in areas not classified by the HOLC (84.35%, generally established post-1935). Of those that lived in areas that were classified, grade C was the most frequent, followed by grades D and B; deaths in areas graded as A represented less than 1% of records. Of those that lived in areas that were not classified, the vast majority lived in block groups that did not touch the areas assessed by the HOLC (99.24); the remainder were assigned as unclassified using the apportionment algorithm. There were also 53 deaths that occurred in areas classified as E which were left as-is since there is no indication as to what this classification could signify. The mean age of cases was 75.96 years with a standard deviation of 14.68 years.

**Table 1:** Baseline characteristics of mortality cases, showing mean ± SD or N (%). Deaths that occurred in block groups

Variable	All included individuals (*n* = 8,884,733)	Individuals from historically-redlined areas (*n* = 189,687)
Age (years)	75.96 (14.68)	72.55 (15.69%)
Sex		
Male	4,322,095 (48.65%)	93,776 (49.44%)
Female	4,562,367 (51.35%)	95,906 (50.56%)
Unknown	271 (0.00%)	5 (0.00%)
Race		
White	7,615,811 (85.72%)	101,892 (53.72%)
Black	917,202 (10.32%)	73,568 (38.78%)
Other	319,191 (3.59%)	13,604 (7.17%)
Unknown	32,529 (0.37%)	623 (0.33%)
Education level		
Less than high school	1,964,879 (22.12%)	63,522 (33.49%)
High school	3,656,272 (41.15%)	76,351 (40.25%)
More than high school	2,888,161 (32.51%)	39,646 (20.90%)
Unknown	375,421 (4.23%)	10,168 (5.36%)
Reporting state		
California (2009–2016)	1,771,689 (19.94%)	48,406 (25.52%)
Florida (2007–2016)	1,583,311 (17.82%)	11,576 (6.10%)
Georgia (2007–2009)	179,105 (2.02%)	1,688 (0.89%)
Illinois (2008–2016)	848,851 (9.55%)	39,819 (20.99%)
Indiana (2007–2008)	97,459 (1.10%)	3,715 (1.96%)
Kansas (2007–2009)	62,279 (0.70%)	1,439 (0.76%)
Massachusetts (2000–2015)	778,304 (8.76%)	23,398 (12.34%)
Michigan (2007–2013)	546,603 (6.15%)	15,707 (8.28%)
Missouri (2010–2016)	354,958 (4.00%)	6,378 (3.36%)
New Hampshire (2007–2016)	85,521 (0.96%)	283 (0.15%)
New Jersey (2004–2009)	349,596 (3.93%)	14,175 (7.47%)
Ohio (2007–2013)	679,135 (7.64%)	9,701 (5.11%)
Texas (2007–2016)	1,547,922 (17.42%)	13,402 (7.07%)
HOLC grade (> 90% block group pop.)		
A (“Best”)	29,605 (0.33%)	N/A
B (“Still Desirable”)	117,492 (1.32%)	N/A
C (“Definitely Declining”)	378,468 (4.26%)	N/A
D (“Hazardous”, i.e. redlined)	189,687 (2.13%)	189,687 (100%)
E (Unknown)	53 (0.00%)	N/A
Ambiguous	675,266 (7.60%)	N/A
Unclassified	7,437,289 (84.35%)	N/A
BG outside of HOLC areas	7,380,316 (99.23%)	N/A

Cases involving individuals who lived in historically-redlined areas (HOLC class D, “Hazardous”) comprised 2.13% of all observed cases. This subpopulation had a higher proportion of people of color (45.95%, compared to 13.91% in the full population) and individuals who had an education level of high school or less (73.74%, compared to 63.27%). The average age of individuals at the time of death was also lower (72.55 years old, compared to 75.96). Higher proportions of cases in this subpopulation came from the states of California, Illinois, Indiana, Kansas, Massachusetts, Michigan, and New Jersey.

Assigned environmental exposures at the time that each case was reported are shown in Table 2. In the full data set, the mean ambient concentration of PM_2.5_ was 9.61 µg m^−3^ (SD 5.72 µg m^−3^) on the day of the case and 9.59 µg m^−3^ (SD 4.51 µg m^−3^) for the moving average comprising the day of the case and the 4 preceding days. 9.57% of cases occurred on extreme heat days, of which 32.63% occurred on the first day of extreme heat. Ambient PM_2.5_ both on the day of the case and in the 5-day moving average were higher for individuals who lived in historically-redlined areas (10.95 µg m^−3^; SD 6.38 µg m^−3^ and 10.91 µg m^−3^; SD 4.67 µg m^−3^, respectively). Additionally, a slightly larger proportion of deaths from historically-redlined areas occurred on days of extreme heat (9.71%). Both ambient temperature and vapor pressure were higher among all included individuals vs. cases among individuals from historically-redlined areas.

**Table 2:** Assessed exposures, showing mean ± SD or N (%)

Variable	All included individuals (*n* = 8,884,733)	Individuals from historically-redlined areas (*n* = 189,687)
Temperature (°C)		
On the day of the death	15.63 (9.89)	13.74 (9.88)
5-day moving average (lags 0–4)	15,59 (9.63)	13.69 (9.59)
Vapor pressure (kPa)		
On the day of the death	1.30 (0.81)	1.15 (0.71)
5-day moving average (lags 0–4)	1.30 (0.77)	1.15 (0.69)
Ambient PM_2.5_ (µg m^−3^)		
On the day of the death	9.61 (5.72)	10.95 (6.38)
5-day moving average (lags 0–6)	9.59 (4.51)	10.91 (4.67)
Extreme heat (> 90th percentile of TMIN)	850,275 (9.57%)	18,426 (9.71%)
1st day in heat wave	277,404 (32.63%)	5,978 (32.44%)
2nd day in heat wave	181,331 (21.33%)	3,898 (21.15%)
3rd day in heat wave	117,652 (13.84%)	2,554 (13.86%)
4th day in heat wave	77,771 (9.15%)	1,657 (8.99%)

**Fig. 4:**
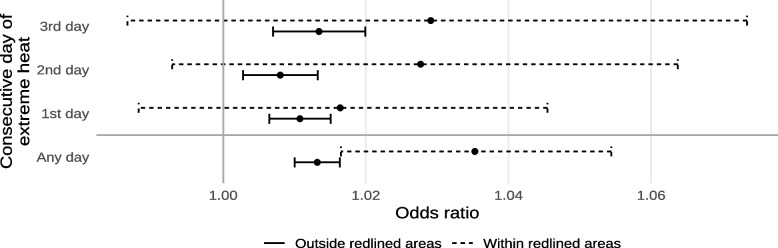
Estimated odds ratios of mortality from non-external causes due to exposure to any extreme heat or the n^th^ consecutive day of extreme heat, within and outside of historically-redlined areas

The estimated effects of exposure to extreme heat on mortality within and outside of historically-redlined neighborhoods are shown in Fig. 4 and Supplement A. In general, results suggested that living in a historically-redlined neighborhood increases susceptibility to death by exposure to extreme heat. We found a significant interaction with exposure to any extreme heat (interaction odds ratio 1.0218; 95% CI 1.0031, 1.0408) while we did not observe significant interactions for singleton heat events or when looking at length-specific exposures. In absolute terms, this amounts to a 2.157% (95% CI 0.307%, 4.036%) increase in the daily risk of death death from non-external causes by exposure to any extreme heat in historically-redlined neighborhoods compared to other neighborhoods. The highest overall effects were observed for exposure to any extreme heat, followed by 3, 1, and 2 consecutive days of extreme heat, respectively.

**Fig. 5:**
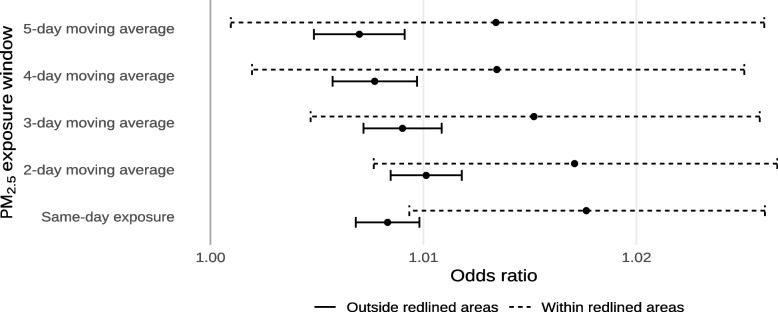
Estimated odds ratios of mortality from non-external causes for each 10 µg/m^−3^ increase in ambient PM_2.5_, or the average ambient PM_2.5_ across multiple days, within and outside of historically-redlined areas

The estimated effects of exposure to ambient PM_2.5_ on mortality within and outside of historically-redlined neighborhoods are shown in Fig. 5 and Supplement B. As with extreme heat, we found that living in a historically-redlined neighborhood increases susceptibility to death by exposure to ambient PM_2.5_. We found a significant interaction with same-day ambient PM_2.5_ (interaction odds ratio for each 10 µg/m^−3^ increase: 1.0093; 95% CI 1.0084, 1.0101) while we did not observe interactions for different moving averages of ambient PM_2.5_. In absolute terms, this amounts to a 0.930% (95% CI 0.831%, 1.000%) increase in the daily risk of death from non-external causes for each 10 µg/m^−3^ increase in ambient PM_2.5_ in historically-redlined neighborhoods compared to other neighborhoods. However, the point estimates for interactions with 2- to 5-day moving averages of ambient PM_2.5_ were similar. The highest overall effect was observed for same-day exposure and the lowest overall effect was observed for the 5-day moving average.

**Fig. 6:**
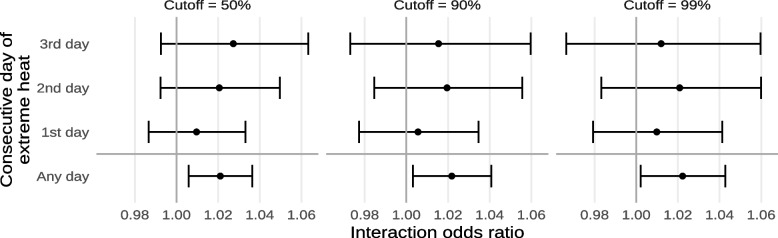
Estimated interaction odds ratios of mortality from non-external causes due to exposure to any extreme heat or the n^th^ consecutive day of extreme heat, using different cutoffs of population for HOLC grade apportionment

**Fig. 7:**
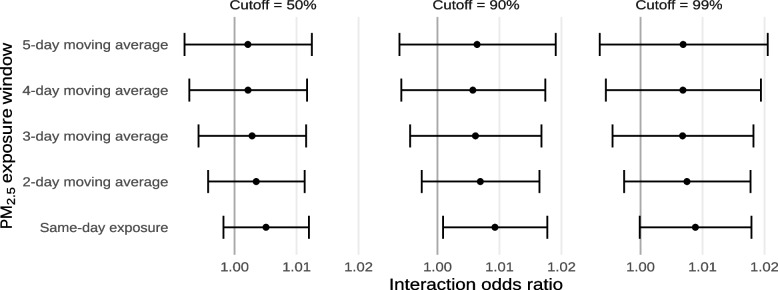
Estimated interaction odds ratios of mortality from non-external causes for each 10 µg/m^−3^ increase in ambient PM_2.5_, or the average ambient PM_2.5_ across multiple days, using different cutoffs of population for HOLC grade apportionment

Results from our sensitivity analyses considering different cutoffs of population for the apportionment between HOLC geographies and Census block groups are shown in Figs. 6 and 7 and Supplements A and B. Among each combination of exposure and exposure window, we did not observe significant differences between the different cutoffs. However, for PM_2.5_, we did observe that the interaction with same-day ambient PM_2.5_ was not significant for population cutoffs of 50% and 99%. We also observed that, for PM_2.5_, estimates using a population cutoff of 50% were smaller.

**Fig. 8:**
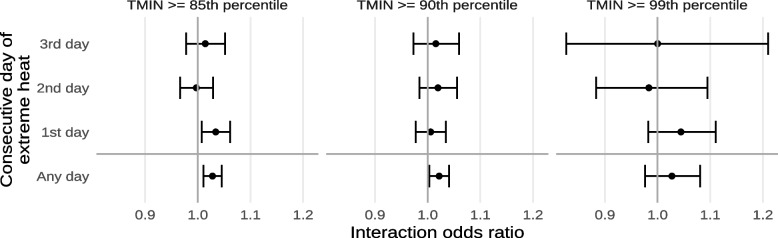
Estimated interaction odds ratios of mortality from non-external causes due to exposure to any extreme heat or the n^th^ consecutive day of extreme heat, using different cutoffs of minimum temperature

Results from our sensitivity analyses considering different cutoffs of minimum temperature for the determination of what constitutes extreme heat are shown in Fig. 8 and Supplement C. We observed that, for the most part, interactions were similar across the different cutoffs. We also observed that the 85th and 95th percentile cutoffs of minimum temperature had higher interactions than the 90th percentile cutoff, with the 85th percentile being the highest for any exposure and the 99th percentile being the highest for the 1st day of extreme heat.

**Fig. 9:**
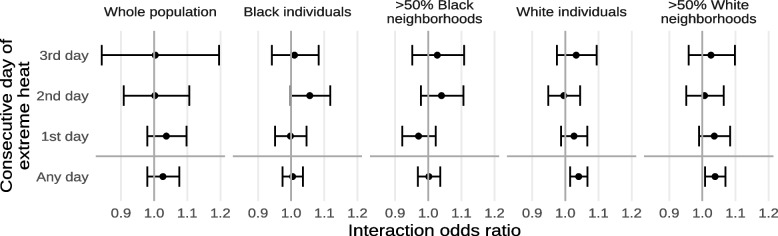
Estimated interaction odds ratios of mortality from non-external causes due to exposure to any extreme heat or the n^th^ consecutive day of extreme heat, restricted to different subsets of the population by individual- and area-level demographics

**Fig. 10:**
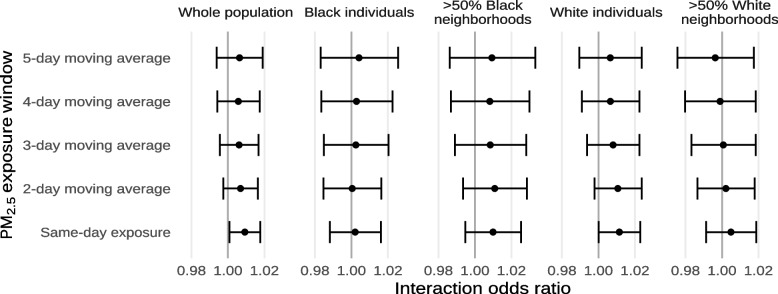
Estimated interaction odds ratios for mortality from non-external causes for each 10 µg/m^−3^ increase in ambient PM_2.5_, or the average ambient PM_2.5_ across multiple days, restricted to different subsets of the population by individual- and area-level demographics

Results from our sensitivity analyses by individual- and area-level demographics are shown in Figs. 9 and 10 and Supplements D and E. For extreme heat, we found that Black individuals and individuals from Black neighborhoods tended to be less susceptible while White individuals and individuals from White neighborhoods were more susceptible. In particular, we found significant interactions between exposure to extreme heat and both self-identification as White and living in a White neighborhood while the corresponding interactions for self-identification as Black and living in a Black neighborhood were close to null. For PM_2.5_, interactions were more similar among the different subgroups.

**Fig. 11:**
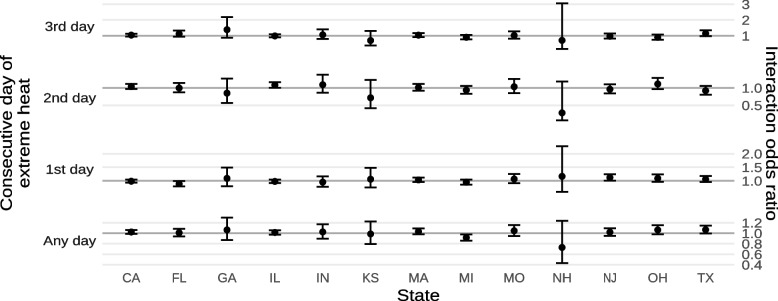
Estimated interaction odds ratios of mortality from non-external causes due to exposure to any extreme heat or the n^th^ consecutive day of extreme heat by state

**Fig. 12:**
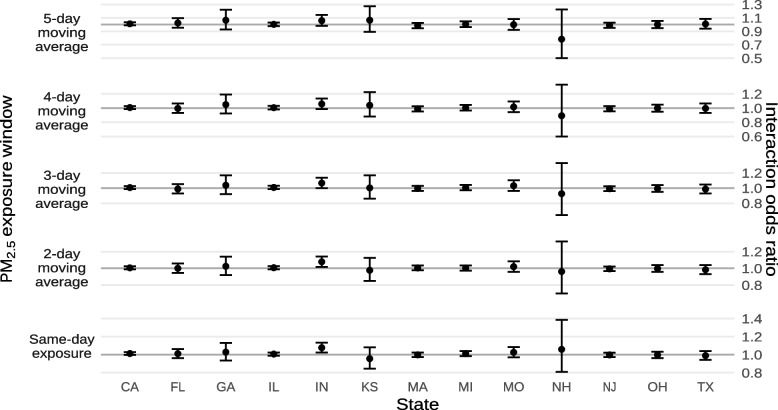
Estimated interaction odds ratios of mortality from non-external causes for each 10 µg/m^−3^ increase in ambient PM_2.5_, or the average ambient PM_2.5_ across multiple days by state

Results from our sensitivity analyses by state are shown in Figs. 11 and 12 and Supplements F and G. In general, we did not observe significant heterogeneity in the interactions between living in a historically-redlined neighborhood and exposure either extreme heat or ambient PM_2.5_, though the interactions between historical redlining and ambient PM_2.5_ tended to be stronger in Indiana.

**Fig. 13:**
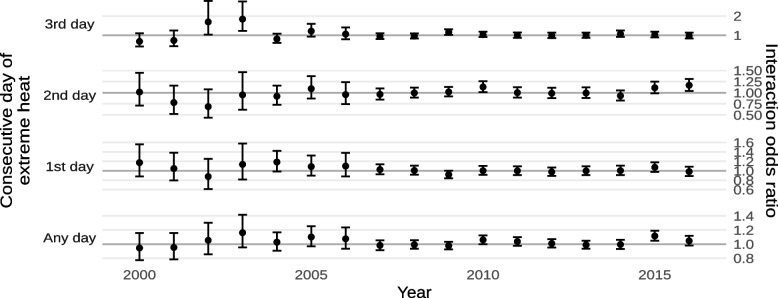
Estimated interaction odds ratios of mortality from non-external causes due to exposure to any extreme heat or the n^th^ consecutive day of extreme heat by year

**Fig. 14:**
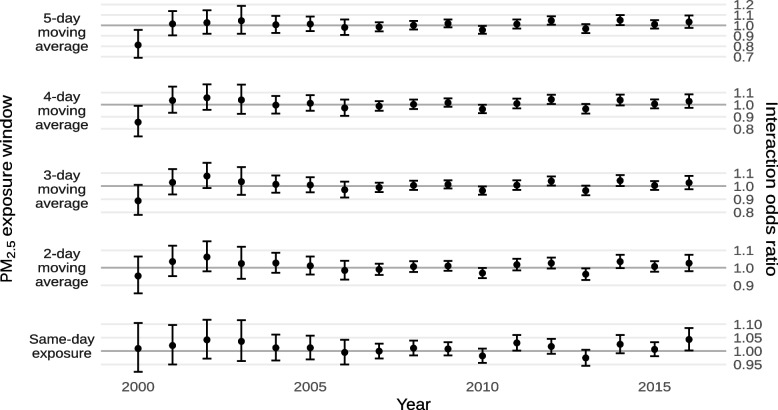
Estimated interaction odds ratios of mortality from non-external causes due for each 10 µg/m^−3^ increase in ambient PM_2.5_, or the average ambient PM_2.5_ across multiple days by year

Results from our sensitivity analyses by year are shown in Figs. 13 and 14 and Supplements H and I. As with our sensitivity analyses by state, there was no clear heterogeneity in the interactions between living in a historically-redlined neighborhood and exposure to either extreme heat or ambient PM_2.5_ by year. There also did not appear to be any consistent time trend in those interactions, though both exposures exhibited suggestive cyclic patterns with multi-year periods.

## Discussion

In this study, we found that some disparities in mortality risks due to exposures to ambient air pollution and extreme heat experienced by individuals living in previously-redlined areas persist nearly a century after the initial creation of the HOLC security maps, and that the injustices fostered by these maps go beyond the quantity of exposure itself and include differential susceptibility. Using a case-crossover design, we were able to control for all time-invariant and individual-level confounders and demonstrated that living in a previously-redlined area has synergistic effects with both ambient PM_2.5_ and exposure to extreme heat on mortality by non-external causes. These findings have implications for policy going forward, as these results suggest that redlined communities will experience air pollution- and extreme heat-related health disparities even after local air pollution levels are brought to levels comparable to not-redlined communities or interventions to reduce heat through green space and reflective sidewalk installations have been implemented.

Notably, 53.72% of the deaths in historically-redlined communities involved White individuals, so these findings are not simply an effect of larger effect sizes in minority population. This was further confirmed by our sensitivity analyses – we did not find any significant differences between the response of Black or White individuals, individuals from present-day majority Black or White neighborhoods, and the population as a whole. Additionally, we found that our results were consistent for different definitions of what Census block groups count as having been redlined and for different definitions of extreme heat.

Interestingly, we observed that the interaction between living in a historically-redlined neighborhood and exposure to extreme heat was stronger in White individuals. It’s unclear what could explain this finding and more work is needed to investigate its robustness and determine potential mechanisms. Previous studies have shown that Black and White individuals spend similar amounts of time outdoors and that Black neighborhoods may have more outdoor amenities, though these amenities tend to be of worse quality, so this may not be an effect of behavioral differences between these subpopulations [5, 11, 18, 23].

Previous studies also identified acute effects of extreme heat on mortality ([3, 22, 30], p. 50). Simultaneously, previous studies have also identified acute effects of PM_2.5_ [17, 19, 24, 42], including studies looking at lags up to 30 or 40 days [47, 48]. The present study complements these prior analyses by contributing evidence that historical redlining modifies those effects, which is an important finding for environmental justice concerns. Moreover, previous literature has found that the effects of both extreme heat and PM_2.5_ exposures are more severe in Black individuals [6, 13, 30, 40, 41, 45], though findings are conflicted. In our sensitivity analysis, we find that the effect modification persists in individual- and neighborhood-level demographic subsets, suggesting that the effect is not simply one of neighborhood composition but rather represents lasting, structural impacts of historic redlining.

The present study has a few major strengths, the most significant being the use of a case-crossover analysis to control for confounding, and the focus on block-groups instead of the more common city level models. By using a case-crossover analysis rather than more traditional epidemiologic methods of confounding control, we were able to significantly limit the number of potential uncontrolled confounders by design. Previous case-crossover studies conducted at a city level used citywide means of PM2.5 or extreme heat, introducing substantial exposure error. Using block-group level exposure reduces this error, better captures urban heat islands, and local adaptation to prevailing temperature. This study also benefits from the generalizability of the data source used – rather than looking at a subset of the population, the death records used encompass the entire population of people who have died in each state during the years collected. Another strength of our study is that our findings remained robust across extensive sensitivity analyses.

This study also has a few limitations. Chiefly, air pollution and meteorology were assessed at the home block group of each individual, which may not have captured their true exposures if they regularly commuted to a work far from home. However, because we looked at acute effects, and the mean age of our population was 76 years old, this misclassification issue is less of a concern. It is also possible that additional factors that influence both air pollution and redlining or both extreme heat and redlining that were not adjusted for may have confounded these findings and biased these results. Additionally, though our data includes deaths from several states across the country with different physical and sociopolitical environments, they are not all-encompassing and it is possible that estimates could be different in areas that we missed, such as in the Pacific Northwest and Great Plains regions. Lastly, these findings also do not identify any single causative agent, as redlining can influence mortality and chronic psychosocial stress in a variety of different ways. This is an important area of future research, as identifying these causative agents will be crucial to designing effective interventions to reduce disparities.

## Conclusions

We have observed that the actions of the HOLC nearly a century ago that upheld structural discrimination through the creation and distribution of its racist security maps are still felt today, and individuals living in affected areas will continue to experience extreme heat- and air pollution-related health disparities even after these adverse environmental agents are mitigated. This study highlights the urgent need for future investigation into the specific causal agents driving this health disparity in order to design specific, targeted interventions that can address both extreme heat and air pollution as well as socioeconomic inequalities present in disadvantaged neighborhoods. In the meantime, interventions to reverse the impact of redlining in general, such as efforts to reduce local PM_2.5_ by increasing access to alternative forms of transportation or plant trees to reduce the effect of urban heat, are a good start. In general, our findings suggest that interventions that focus on the environment alone may not be able to fully achieve environmental health equity and a more holistic approach may be more well-suited to achieving these goals.

### Supplementary Information

**Additional file 1: Supplement A.** Estimated odds ratios for the main effects of exposure to extreme heat on mortality and interactions with HOLC grade D, using different cutoffs for HOLC grade apportionment. **Supplement B.** Estimated odds ratios for the main effects of each 10 µg/m^-3^ increase in ambient PM_2.5_ on mortality and interactions with HOLC grade D, using different cutoffs for HOLC grade apportionment. **Supplement C.** Estimated odds ratios for the main effects of exposure to extreme heat on mortality and interactions with HOLC grade D, using different cutoffs for extreme heat. **Supplement D.** Estimated odds ratios for the main effects of exposure to extreme heat on all-cause mortality and interactions with HOLC grade D, within different subpopulations. **Supplement E.** Estimated odds ratios for the main effects of each 10 µg/m_-3_ increase in ambient PM^2.5^ on all-cause mortality and interactions with HOLC grade D, within different subpopulations. **Supplement F.** Estimated odds ratios for the main effects of exposure to extreme heat on mortality and interactions with HOLC grade D, by state. **Supplement G.** Estimated odds ratios for the main effects of exposure to PM_2.5_ on mortality and interactions with HOLC grade D, by state. **Supplement H.** Estimated odds ratios for the main effects of exposure to extreme heat on mortality and interactions with HOLC grade D, by year. **Supplement I.** Estimated odds ratios for the main effects of exposure to PM_2.5_ on mortality and interactions with HOLC grade D, by year.

## Data Availability

The mortality data that support the findings of this study were obtained from the departments of public health of the states of California, Florida, Georgia, Illinois, Indiana, Kansas, Massachusetts, Michigan, Missouri, New Hampshire, New Jersey, Ohio, and Texas under data sharing agreements that do not allow these data to be shared. Exposure data is publicly available at the following locations: • Home Owners Loan Corporation security maps: https://dsl.richmond.edu/panorama/redlining/ • Daymet V4 meteorological predictions: https://daymet.ornl.gov/ • Ambient PM_2.5_ predictions: https://sedac.ciesin.columbia.edu/data/set/aqdh-pm2-5-concentrations-contiguous-us-1-km-2000-2016
