# Correction: Modification of the PM_2.5_- and extreme heat-mortality relationships by historical redlining: A case-crossover study in thirteen U.S. states

**DOI:** 10.1186/s12940-024-01072-4

**Published:** 2024-04-02

**Authors:** Edgar Castro, Abbie Liu, Yaguang Wei, Anna Kosheleva, Joel Schwartz

**Affiliations:** Harvard T.H. Chan School of Public Health, Boston, MA 02115 USA

**Correction:** **Environ Health 23, 16 (2024)**

**https://doi.org/10.1186/s12940-024-01055-5**

Following publication of [1], errors were found in the code used to prepare the cohort for a case-crossover analysis and the resulting data that was used for the analysis. Despite these errors, results were only marginally effected and all conclusions remain the same. A few typos were also found in the manuscript. A table of all affected texts is shown below.

**Table:** 

Section	Lines	Text
Abstract	48-51	Individuals who lived in redlined areas had an interaction odds ratio for mortality of 1.0093 **1.0104** (95% confidence interval [CI]: 1.0084 **1.0095**, 1.0101 **1.0114)** for each 10 µg m^-3^ increase in same-day ambient PM2.5 compared to individuals who did not live in redlined areas. For extreme heat, the interaction odds ratio was 1.0218 **1.0146** (95% CI 1.0031 **1.0039**, 1.0408 **1.0457**).
Methods	159-161	To derive measures of extreme heat, we first calculated various percentiles of minimum temperature in each block group in each year. For our main analysis, we considered the 95 ^th^ **90** ^**th**^ percentile.
Methods	163-165	In other words, if the minimum temperature on a certain day met or exceeded the 95 ^th^ **90** ^**th**^ percentile of minimum temperature in that block group in that year, then that day was marked as an extreme heat day.
Results	229-237	We obtained 11,115,380 **11,076,020** mortality records from the twelve **thirteen** state departments of public health. From these records, we sequentially excluded 466,874 **453,754** deaths involving external causes; 139,908 **133,348** deaths involving individuals younger than 18 years old; 196,558 deaths with geocodes that were missing or coarser than block group-level; 331 deaths involving individuals whose home locations were outside of the state that reported their death; 1,392,423 **1,372,743** deaths before January 5^th^, 2001 or after December 31^st^, 2016 and 537 deaths whose home block groups had a population of zero according to the preceding Decennial Census (for which 4-day moving averages of population-weighted PM2.5 could not be calculated); and 34,016 deaths with lag days from 0 to 4 that included December 31^st^ on leap years (for which Daymet predictions are not available; Figure 3)
Results	272-278	We found a significant interaction with exposure to any extreme heat (interaction odds ratio 1.0218 **1.0246**; 95% CI 1.0031 **1.0039**, 1.0408 **1.0457**) while we did not observe significant interactions for singleton heat events or when looking at length-specific exposures. In absolute terms, this amounts to a 2.157% **2.434%** (95% CI 0.307% **0.386%**, 4.036% **4.521%**) increase in the daily risk of death death from non-external causes by exposure to any extreme heat in historically-redlined neighborhoods compared to other neighborhoods. The highest overall effects were observed for exposure to any extreme heat, followed by 3, 1 **2**, and 2 **1** consecutive days of extreme heat, respectively.
Results	283-287	We found a significant interaction with same-day ambient PM2.5 (interaction odds ratio for each 10 µg/m^-3^ increase: 1.0093 **1.0104**; 95% CI 1.0084 **1.0095**, 1.0101 **1.0114**) while we did not observe interactions for different moving averages of ambient PM2.5. In absolute terms, this amounts to a 0.930% **1.029%** (95% CI 0.831% **0.940%**, 1.000% **1.128%**) increase in the daily risk of death from non-external causes for each 10 µg/m-3 increase in ambient PM2.5 in historically-redlined neighborhoods compared to other neighborhoods.
Results	295-296	However, for PM2.5, we did observe that the interaction with same-day ambient PM2.5 was not significant for **a** population cutoffs of 50% and 99%.
Results	302-305	We also observed that the 85^th^ and 95^th^ percentile cutoffs of minimum temperature had higher interactions than the 90^th^ percentile cutoff **on earlier days**, with the 85^th^ percentile being the highest for any exposure** or the 1** ^**st**^ ** day of extreme heat **and the 99^th^ percentile being the highest for **any exposure or **the 1^st^ ** or 2** ^**nd**^ day**s** of extreme heat.

Firstly, 39,360 deaths from the raw data were duplicated (0.36% of total deaths), resulting in an erroneous Fig. 1 and numbers in the accompanying paragraph. However, the total number of deaths used for the analysis remained the same, though this was misreported in the original Fig. 1 (884,733 deaths rather than 8,884,733). A few percentages were also incorrect. The corrected Fig. 1 is shown below next to the original Fig. 1, followed by a table of changes in the text.

**Figure:**
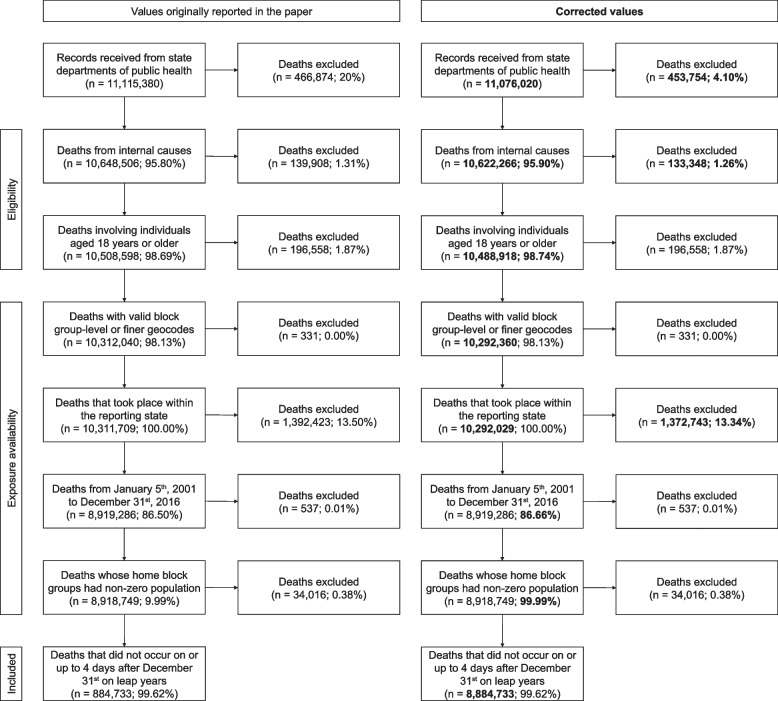
* Original and corrected Fig. 1 with changed bolded*.

Additionally, code that was meant to restrict control days only to those occurring in the same month of the case was not run, resulting in a mean of 7.91 controls for each case (SD: 0.57). After corrections, this was reduced to a mean of 3.38 controls per case (SD: 0.49). Unlike the previous error, this slightly altered the results, though only marginally. The corrected text, figures, and coefficient tables are presented below alongside what was originally reported. Old and new results are differentiated by different shades in the figures and changes in significance are noted in bold in the tables.

**Figure:**
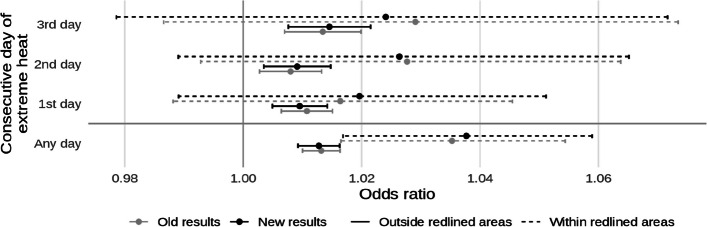
* Original and corrected Fig. 4 combined*.

**Figure:**
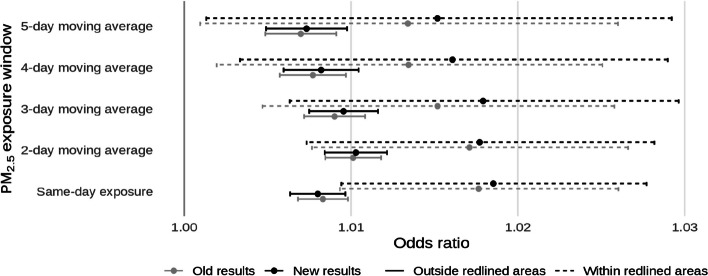
* Original and corrected Fig. 5 combined*.

**Figure:**
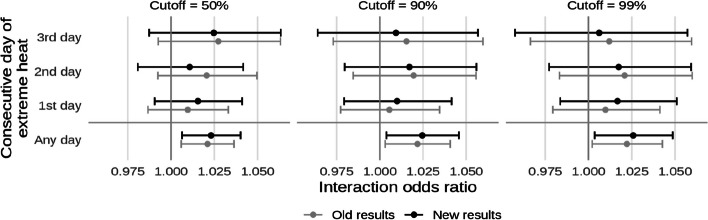
* Original and corrected Fig. 6 combined*.

**Figure:**
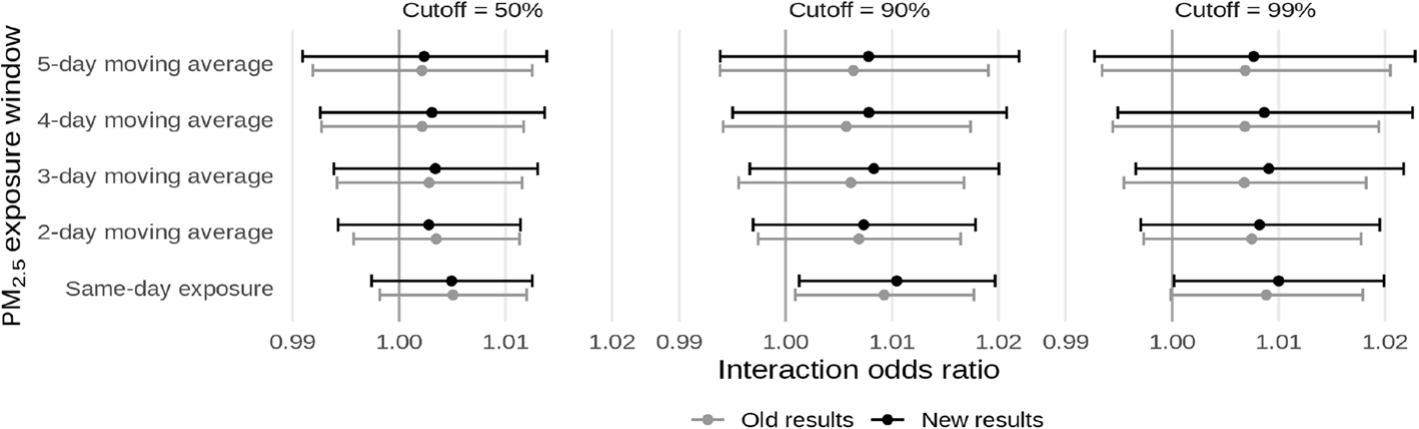
* Original and corrected Fig. 7 combined*.

**Figure:**
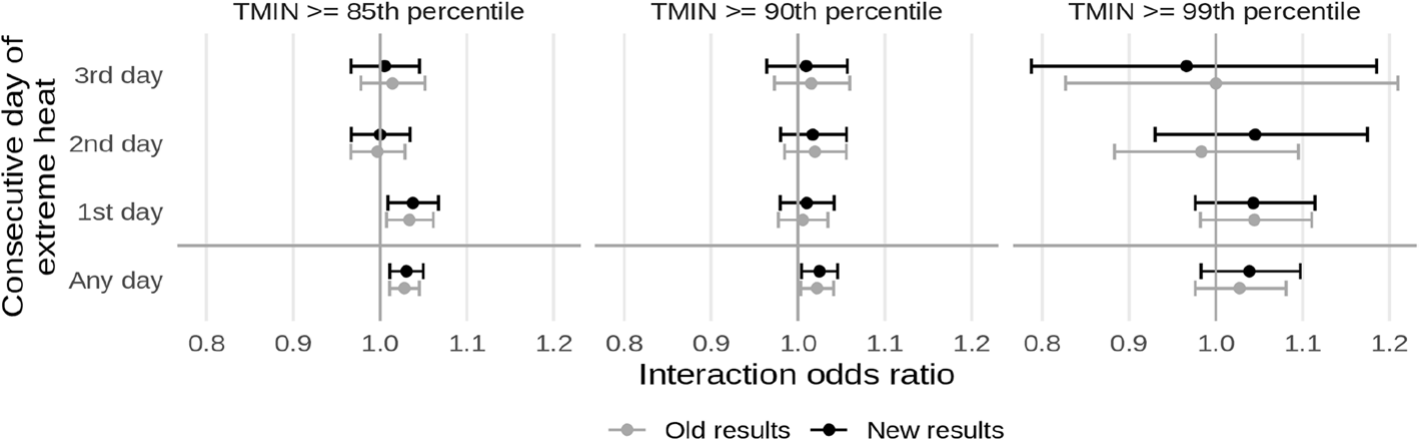
* Original and corrected Fig. 8 combined*.

**Figure:**
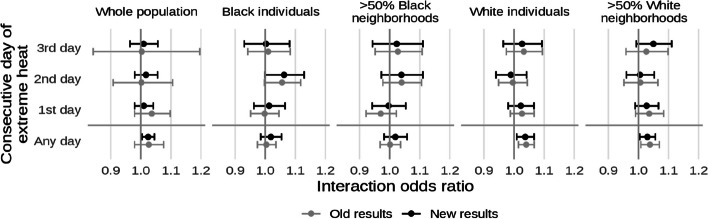
* Original and corrected Fig. 9 combined. The plots for “Whole population” originally showed results from the sensitivity analysis of 99% population cutoff for HOLC apportionment rather than the main analysis of 90%. As a result, the confidence intervals for the new results are smaller*.

**Figure:**
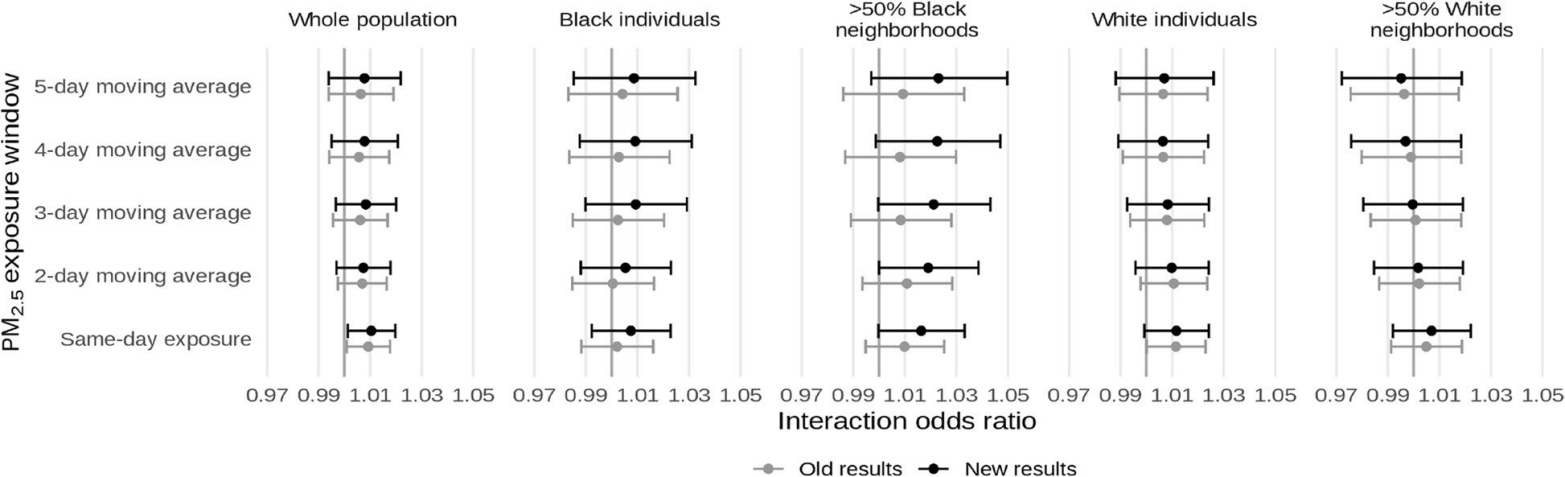
* Original and corrected Fig. 10 combined*.

**Figure:**
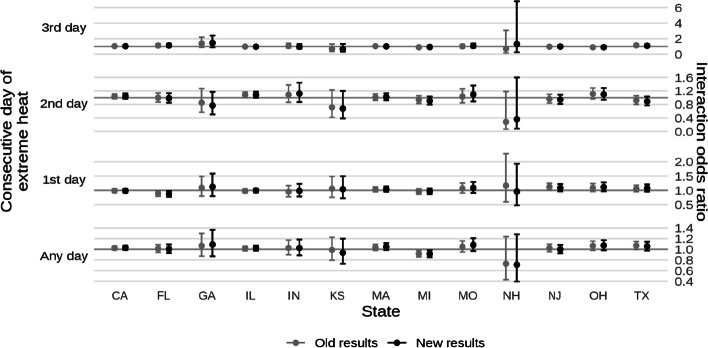
* Original and corrected Fig. 11 combined*.

**Figure:**
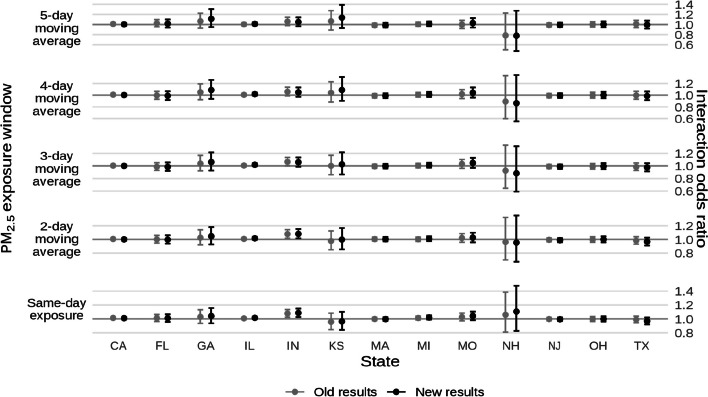
* Original and corrected Fig. 12 combined*.

**Figure:**
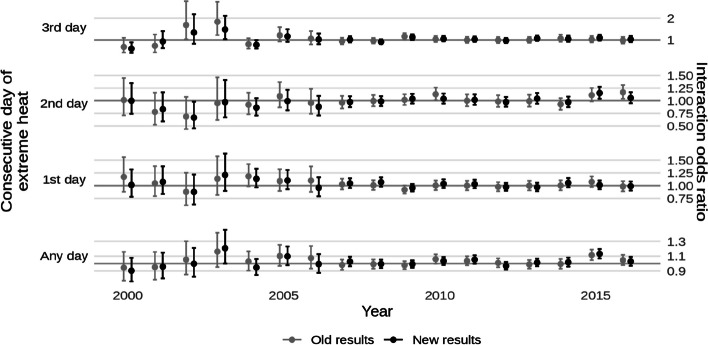
* Original and corrected Fig. 13 combined*.

**Figure:**
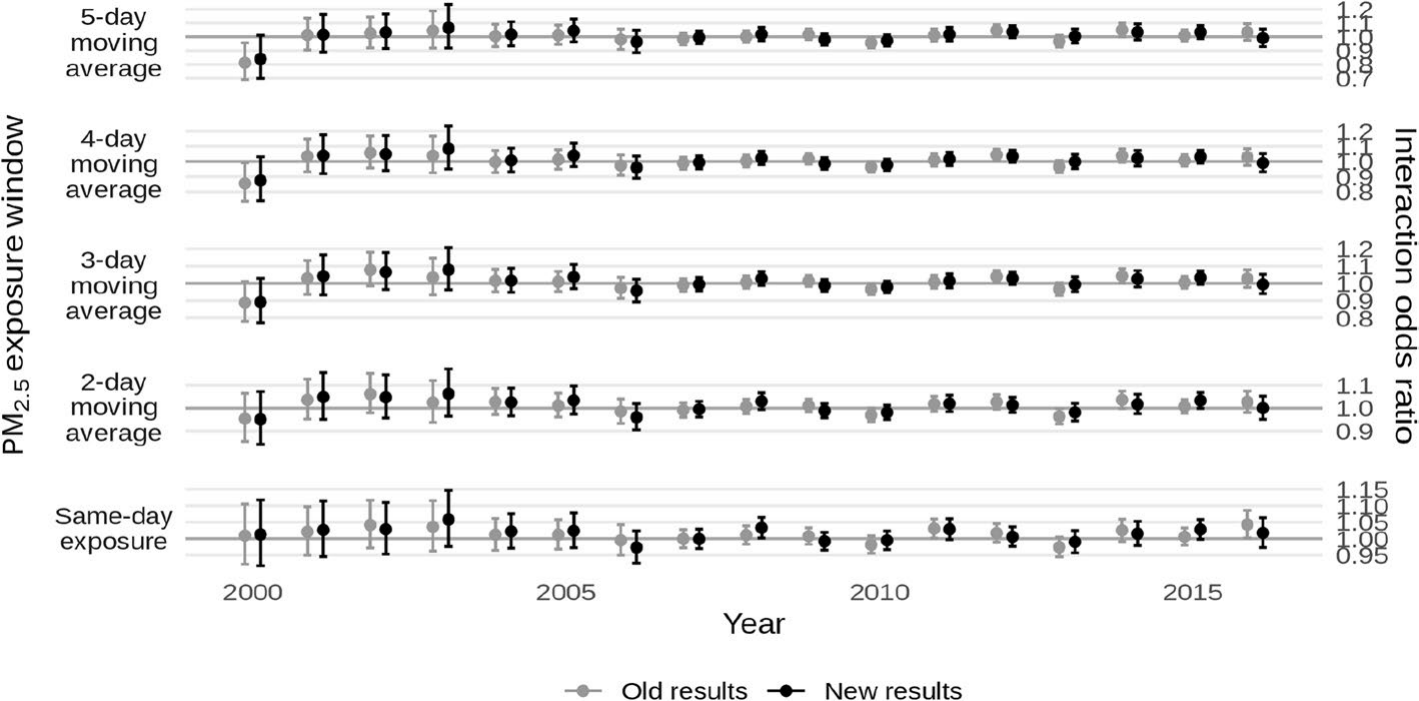
* Original and corrected Fig. 14 combined*.

**Table:** *Original and corrected estimates for main effects from Supplement A.*

	Old estimate				New estimate			
Heat day	Estimate	2.5%	97.5%	p	Estimate	2.5%	97.5%	p
Population cutoff for apportionment = 50%								
Any day	1.013	1.0098	1.0161	< 0.05	1.0125	1.0090	1.0161	< 0.05
1st day	1.011	1.0062	1.0149	< 0.05	1.0093	1.0046	1.0139	< 0.05
2nd day	1.008	1.0025	1.0130	< 0.05	1.0091	1.0035	1.0148	< 0.05
3rd day	1.013	1.0064	1.0194	< 0.05	1.0139	1.0070	1.0210	< 0.05
Population cutoff for apportionment = 90%								
Any day	1.013	1.0100	1.0164	< 0.05	1.0128	1.0093	1.0163	< 0.05
1st day	1.011	1.0065	1.0151	< 0.05	1.0096	1.0049	1.0142	< 0.05
2nd day	1.008	1.0028	1.0133	< 0.05	1.0091	1.0035	1.0148	< 0.05
3rd day	1.013	1.0070	1.0199	< 0.05	1.0146	1.0076	1.0215	< 0.05
Population cutoff for apportionment = 99%								
Any day	1.013	1.0101	1.0164	< 0.05	1.0128	1.0093	1.0163	< 0.05
1st day	1.011	1.0064	1.0150	< 0.05	1.0095	1.0048	1.0141	< 0.05
2nd day	1.008	1.0028	1.0133	< 0.05	1.0092	1.0035	1.0148	< 0.05
3rd day	1.014	1.0071	1.0200	< 0.05	1.0146	1.0077	1.0216	< 0.05

**Table:** *Original and corrected estimates for interactions from Supplement A.*

	Old estimate				New estimate			
Heat day	Estimate	2.5%	97.5%	p	Estimate	2.5%	97.5%	p
Population cutoff for apportionment = 50%								
Any day	1.0211	1.0059	1.0364	< 0.05	1.0232	1.0064	1.0402	< 0.05
1st day	1.0096	0.9867	1.0332	0.41	1.0155	0.9906	1.0411	0.23
2nd day	1.0206	0.9923	1.0496	0.16	1.0108	0.9808	1.0417	0.49
3rd day	1.0273	0.9926	1.0633	0.13	1.0247	0.9874	1.0634	0.20
Population cutoff for apportionment = 90%								
Any day	1.0218	1.0031	1.0408	< 0.05	1.0246	1.0039	1.0457	< 0.05
1st day	1.0056	0.9773	1.0346	0.70	1.0100	0.9794	1.0415	0.53
2nd day	1.0195	0.9846	1.0557	0.28	1.0171	0.9797	1.0559	0.37
3rd day	1.0155	0.9731	1.0597	0.48	1.0094	0.9641	1.0568	0.69
Population cutoff for apportionment = 99%								
Any day	1.0222	1.0021	1.0428	< 0.05	1.0259	1.0036	1.0487	< 0.05
1st day	1.0098	0.9793	1.0413	0.53	1.0168	0.9836	1.0510	0.32
2nd day	1.0208	0.9832	1.0599	0.28	1.0174	0.9772	1.0593	0.40
3rd day	1.0119	0.9664	1.0596	0.61	1.0061	0.9575	1.0572	0.81

**Table:** *Original and corrected estimates for main effects from Supplement B.*

	Old estimate				New estimate			
Exposure	Estimate	2.5%	97.5%	p	Estimate	2.5%	97.5%	p
Population cutoff for apportionment = 50%								
Lag 0	1.0084	1.0082	1.0085	< 0.05	1.0081	1.0079	1.0083	< 0.05
Lags 0–1	1.0102	1.0100	1.0103	< 0.05	1.0104	1.0102	1.0106	< 0.05
Lags 0–2	1.0091	1.0089	1.0093	< 0.05	1.0096	1.0094	1.0098	< 0.05
Lags 0–3	1.0078	1.0076	1.0080	< 0.05	1.0083	1.0081	1.0085	< 0.05
Lags 0–4	1.0084	1.0082	1.0085	< 0.05	1.0074	1.0072	1.0077	< 0.05
Population cutoff for apportionment = 90%								
Lag 0	1.0083	1.0082	1.0085	< 0.05	1.0080	1.0078	1.0082	< 0.05
Lags 0–1	1.0101	1.0100	1.0103	< 0.05	1.0103	1.0101	1.0105	< 0.05
Lags 0–2	1.0090	1.0088	1.0092	< 0.05	1.0096	1.0093	1.0098	< 0.05
Lags 0–3	1.0077	1.0075	1.0079	< 0.05	1.0082	1.0080	1.0084	< 0.05
Lags 0–4	1.0070	1.0068	1.0072	< 0.05	1.0073	1.0071	1.0076	< 0.05
Population cutoff for apportionment = 99%								
Lag 0	1.0084	1.0082	1.0085	< 0.05	1.0081	1.0079	1.0082	< 0.05
Lags 0–1	1.0101	1.0100	1.0103	< 0.05	1.0103	1.0101	1.0105	< 0.05
Lags 0–2	1.0090	1.0088	1.0092	< 0.05	1.0096	1.0094	1.0098	< 0.05
Lags 0–3	1.0077	1.0075	1.0079	< 0.05	1.0082	1.0080	1.0084	< 0.05
Lags 0–4	1.0070	1.0068	1.0072	< 0.05	1.0074	1.0071	1.0076	< 0.05

**Table:** *Original and corrected estimates for interactions from Supplement B.*

	Old estimate				New estimate			
Exposure	Estimate	2.5%	97.5%	p	Estimate	2.5%	97.5%	p
Population cutoff for apportionment = 50%								
Lag 0	1.0051	1.0044	1.0058	0.15	1.0049	1.0042	1.0057	0.2
Lags 0–1	1.0035	1.0027	1.0043	0.38	1.0028	1.0019	1.0037	0.52
Lags 0–2	1.0028	1.0020	1.0037	0.52	1.0034	1.0024	1.0044	0.48
Lags 0–3	1.0022	1.0012	1.0031	0.65	1.0031	1.0020	1.0041	0.57
Lags 0–4	1.0021	1.0011	1.0032	0.68	1.0024	1.0012	1.0035	0.69
Population cutoff for apportionment = 90%								
Lag 0	1.0093	1.0084	1.0101	< 0.05	1.0104	1.0095	1.0114	< 0.05
Lags 0–1	1.0069	1.0059	1.0078	0.15	1.0073	1.0063	1.0084	0.17
Lags 0–2	1.0061	1.0051	1.0072	0.26	1.0083	1.0071	1.0095	0.16
Lags 0–3	1.0057	1.0045	1.0069	0.34	1.0078	1.0065	1.0091	0.23
Lags 0–4	1.0064	1.0051	1.0076	0.32	1.0078	1.0064	1.0092	0.27
Population cutoff for apportionment = 99%								
Lag 0	1.0088	1.0079	1.0098	0.05	1.0100	1.0090	1.0110	< 0.05
Lags 0–1	1.0075	1.0065	1.0085	0.15	1.0082	1.0071	1.0093	0.15
Lags 0–2	1.0068	1.0056	1.0079	0.24	1.0091	1.0078	1.0103	0.15
Lags 0–3	1.0068	1.0056	1.0081	0.28	1.0087	1.0073	1.0100	0.22
Lags 0–4	1.0069	1.0055	1.0082	0.32	1.0077	1.0062	1.0092	0.32

**Table:** *Original and corrected estimates for main effects from Supplement C.*

	Old estimate				New estimate			
Heat day	Estimate	2.5%	97.5%	p	Estimate	2.5%	97.5%	p
Minimum temperature cutoff for extreme heat = 85th percentile								
Any day	1.0109	1.0080	1.0138	< 0.05	1.0117	1.0084	1.0149	< 0.05
1st day	1.0069	1.0030	1.0109	< 0.05	1.0080	1.0037	1.0122	< 0.05
2nd day	1.0124	1.0077	1.0171	< 0.05	1.0135	1.0085	1.0186	< 0.05
3rd day	1.0079	1.0024	1.0133	< 0.05	1.0086	1.0028	1.0145	< 0.05
Minimum temperature cutoff for extreme heat = 90th percentile								
Any day	1.0132	1.0100	1.0164	< 0.05	1.0128	1.0093	1.0163	< 0.05
1st day	1.0108	1.0065	1.0151	< 0.05	1.0096	1.0049	1.0142	< 0.05
2nd day	1.0080	1.0028	1.0133	< 0.05	1.0091	1.0035	1.0148	< 0.05
3rd day	1.0134	1.0070	1.0199	< 0.05	1.0146	1.0076	1.0215	< 0.05
Minimum temperature cutoff for extreme heat = 99th percentile								
Any day	1.0217	1.0143	1.0292	< 0.05	1.0211	1.0130	1.0293	< 0.05
1st day	1.0181	1.0092	1.0271	< 0.05	1.0176	1.0080	1.0273	< 0.05
2nd day	1.0227	1.0081	1.0376	< 0.05	1.0193	1.0036	1.0353	< 0.05
3rd day	1.0501	1.0238	1.0771	< 0.05	1.0623	1.0336	1.0917	< 0.05

**Table:** *Original and corrected estimates for interactions from Supplement C.*

	Old estimate				New estimate			
Heat day	Estimate	2.5%	97.5%	p	Estimate	2.5%	97.5%	p
Minimum temperature cutoff for extreme heat = 85th percentile								
Any day	1.0279	1.0109	1.045	< 0.05	1.0302	1.0110	1.0497	< 0.05
1st day	1.0340	1.0074	1.061	< 0.05	1.0378	1.0090	1.0674	< 0.05
2nd day	0.9972	0.9665	1.029	0.86	1.0001	0.9670	1.0344	1.00
3rd day	1.0142	0.9779	1.052	0.45	1.0052	0.9666	1.0454	0.79
Minimum temperature cutoff for extreme heat = 90th percentile								
Any day	1.0218	1.0031	1.041	< 0.05	1.0246	1.0039	1.0457	< 0.05
1st day	1.0056	0.9773	1.035	0.70	1.0100	0.9794	1.0415	0.53
2nd day	1.0195	0.9846	1.056	0.28	1.0171	0.9797	1.0559	0.37
3rd day	1.0155	0.9731	1.060	0.48	1.0094	0.9641	1.0568	0.69
Minimum temperature cutoff for extreme heat = 99th percentile								
Any day	1.0266	0.9793	1.076	0.28	1.0350	0.9836	1.0892	0.19
1st day	1.0368	0.9793	1.098	0.21	1.0344	0.9727	1.1001	0.28
2nd day	1.0021	0.9081	1.106	0.97	1.0553	0.9482	1.1746	0.32
3rd day	1.0034	0.8419	1.196	0.97	0.9774	0.8095	1.1802	0.81

**Table:** *Original and corrected estimates for main effects from Supplement D.*

	Old estimate				New estimate			
Heat day	Estimate	2.5%	97.5%	p	Estimate	2.5%	97.5%	p
Whole population								
Any day	1.0132	1.0100	1.0164	< 0.05	1.0128	1.0093	1.0163	< 0.05
1st day	1.0108	1.0065	1.0151	< 0.05	1.0096	1.0049	1.0142	< 0.05
2nd day	1.0080	1.0028	1.0133	< 0.05	1.0091	1.0035	1.0148	< 0.05
3rd day	1.0134	1.0070	1.0199	< 0.05	1.0146	1.0076	1.0215	< 0.05
Black individuals								
Any day	1.0058	0.9956	1.0160	0.27	1.0052	0.9939	1.0165	0.37
1st day	1.0008	0.9872	1.0146	0.91	0.9984	0.9839	1.0132	0.83
2nd day	0.9975	0.9810	1.0141	0.76	0.9971	0.9794	1.0150	0.75
3rd day	1.0191	0.9988	1.0399	0.07	1.0209	0.9989	1.0433	0.06
> 50% Black neighborhoods								
Any day	1.0067	0.9936	1.0201	0.32	1.0011	0.9865	1.0159	0.88
1st day	1.0086	0.9913	1.0261	0.33	1.0010	0.9826	1.0198	0.91
2nd day	1.0007	0.9801	1.0218	0.94	0.9969	0.9748	1.0195	0.78
3rd day	1.0133	0.9877	1.0395	0.31	1.0142	0.9867	1.0425	0.32
White individuals								
Any day	1.0141	1.0107	1.0175	< 0.05	1.0139	1.0101	1.0177	< 0.05
1st day	1.0127	1.0080	1.0173	< 0.05	1.0118	1.0068	1.0168	< 0.05
2nd day	1.0089	1.0033	1.0145	< 0.05	1.0099	1.0038	1.0159	< 0.05
3rd day	1.0110	1.0041	1.0180	< 0.05	1.0123	1.0048	1.0198	< 0.05
> 50% White neighborhoods								
Any day	1.0128	1.0093	1.0162	< 0.05	1.0125	1.0086	1.0163	< 0.05
1st day	1.0118	1.0071	1.0164	< 0.05	1.0110	1.0060	1.0161	< 0.05
2nd day	1.0060	1.0003	1.0116	< 0.05	1.0070	1.0009	1.0131	< 0.05
3rd day	1.0139	1.0069	1.0209	< 0.05	1.0148	1.0073	1.0224	< 0.05

**Table:** *Original and corrected estimates for interactions from Supplement D.*

	Old estimate				New estimate			
Heat day	Estimate	2.5%	97.5%	p	Estimate	2.5%	97.5%	p
Whole population								
Any day	1.0218	1.0031	1.0408	< 0.05	1.0246	1.0039	1.0457	< 0.05
1st day	1.0056	0.9773	1.0346	0.70	1.0100	0.9794	1.0415	0.53
2nd day	1.0195	0.9846	1.0557	0.28	1.0171	0.9797	1.0559	0.37
3rd day	1.0155	0.9731	1.0597	0.48	1.0094	0.9641	1.0568	0.69
Black individuals								
Any day	1.0043	0.9739	1.0356	0.79	1.0190	0.9850	1.0542	0.28
1st day	0.9977	0.9516	1.0460	0.92	1.0128	0.9624	1.0659	0.62
2nd day	1.0561	0.9978	1.1178	0.06	1.0625	0.9995	1.1295	0.05
3rd day	1.0099	0.9417	1.0830	0.78	1.0029	0.9304	1.0811	0.94
> 50% Black neighborhoods								
Any day	1.0017	0.9685	1.0361	0.92	1.0193	0.9819	1.0580	0.32
1st day	0.9707	0.9216	1.0223	0.26	0.9962	0.9420	1.0537	0.9
2nd day	1.0399	0.9775	1.1062	0.22	1.0393	0.9724	1.1107	0.26
3rd day	1.0272	0.9520	1.1084	0.49	1.0236	0.9433	1.1108	0.58
White individuals								
Any day	1.0403	1.0146	1.0667	< 0.05	1.0373	1.0090	1.0665	< 0.05
1st day	1.0262	0.9874	1.0666	0.19	1.0225	0.9808	1.0660	0.29
2nd day	0.9954	0.9487	1.0443	0.85	0.9894	0.9397	1.0418	0.69
3rd day	1.0327	0.9745	1.0944	0.28	1.0266	0.9643	1.0930	0.41
> 50% White neighborhoods								
Any day	1.0383	1.0079	1.0695	< 0.05	1.0296	1.0038	1.0560	< 0.05
1st day	1.0359	0.9903	1.0836	0.12	1.0269	0.9890	1.0662	0.17
2nd day	1.0064	0.9516	1.0645	0.82	1.0051	0.9599	1.0524	0.83
3rd day	1.0259	0.9585	1.0980	0.46	1.0499	0.9927	1.1104	0.09

**Table:** *Original and corrected estimates for main effects from Supplement E.*

	Old estimate				New estimate			
Heat day	Estimate	2.5%	97.5%	p	Estimate	2.5%	97.5%	p
Whole population								
Lag 0	1.0083	1.0082	1.0085	< 0.05	1.0080	1.0078	1.0082	< 0.05
Lags 0–1	1.0101	1.0100	1.0103	< 0.05	1.0103	1.0101	1.0105	< 0.05
Lags 0–2	1.0090	1.0088	1.0092	< 0.05	1.0096	1.0093	1.0098	< 0.05
Lags 0–3	1.0077	1.0075	1.0079	< 0.05	1.0082	1.0080	1.0084	< 0.05
Lags 0–4	1.0070	1.0068	1.0072	< 0.05	1.0073	1.0071	1.0076	< 0.05
Black individuals								
Lag 0	1.0117	1.0112	1.0121	< 0.05	1.0105	1.0100	1.0110	< 0.05
Lags 0–1	1.0130	1.0124	1.0135	< 0.05	1.0125	1.0120	1.0131	< 0.05
Lags 0–2	1.0130	1.0124	1.0136	< 0.05	1.0139	1.0132	1.0145	< 0.05
Lags 0–3	1.0128	1.0122	1.0134	< 0.05	1.0145	1.0138	1.0152	< 0.05
Lags 0–4	1.0127	1.0121	1.0134	< 0.05	1.0152	1.0145	1.0160	< 0.05
> 50% Black neighborhoods								
Lag 0	1.0065	1.0059	1.0071	< 0.05	1.0067	1.0060	1.0073	< 0.05
Lags 0–1	1.0060	1.0053	1.0066	0.08	1.0072	1.0065	1.0079	0.05
Lags 0–2	1.0061	1.0053	1.0068	0.10	1.0092	1.0084	1.0100	**< 0.05**
Lags 0–3	1.0039	1.0031	1.0047	0.34	1.0081	1.0072	1.0090	0.07
Lags 0–4	1.0019	1.0011	1.0028	0.66	1.0076	1.0067	1.0086	0.12
White individuals								
Lag 0	1.0081	1.0079	1.0082	< 0.05	1.0080	1.0078	1.0081	< 0.05
Lags 0–1	1.0100	1.0098	1.0101	< 0.05	1.0104	1.0102	1.0106	< 0.05
Lags 0–2	1.0086	1.0084	1.0088	< 0.05	1.0093	1.0091	1.0096	< 0.05
Lags 0–3	1.0071	1.0069	1.0073	< 0.05	1.0078	1.0076	1.0081	< 0.05
Lags 0–4	1.0063	1.0061	1.0066	< 0.05	1.0069	1.0067	1.0072	< 0.05
> 50% White neighborhoods								
Lag 0	1.0079	1.0077	1.0081	< 0.05	1.0083	1.0082	1.0085	< 0.05
Lags 0–1	1.0098	1.0096	1.0100	< 0.05	1.0108	1.0106	1.0110	< 0.05
Lags 0–2	1.0082	1.0080	1.0084	< 0.05	1.0096	1.0094	1.0098	< 0.05
Lags 0–3	1.0067	1.0065	1.0069	< 0.05	1.0081	1.0078	1.0083	< 0.05
Lags 0–4	1.0058	1.0056	1.0061	< 0.05	1.0072	1.0070	1.0075	< 0.05

**Table:** *Original and corrected estimates for interactions from Supplement E.*

	Old estimate				New estimate			
Heat day	Estimate	2.5%	97.5%	p	Estimate	2.5%	97.5%	p
Whole population								
Lag 0	1.0093	1.0084	1.0101	< 0.05	1.0104	1.0095	1.0114	< 0.05
Lags 0–1	1.0069	1.0059	1.0078	0.15	1.0073	1.0063	1.0084	0.17
Lags 0–2	1.0061	1.0051	1.0072	0.26	1.0083	1.0071	1.0095	0.16
Lags 0–3	1.0057	1.0045	1.0069	0.34	1.0078	1.0065	1.0091	0.23
Lags 0–4	1.0064	1.0051	1.0076	0.32	1.0078	1.0064	1.0092	0.27
Black individuals								
Lag 0	1.0020	1.0006	1.0034	0.78	1.0074	1.0059	1.0090	0.34
Lags 0–1	1.0004	0.9988	1.0020	0.96	1.0053	1.0036	1.0071	0.55
Lags 0–2	1.0024	1.0006	1.0042	0.79	1.0093	1.0073	1.0113	0.35
Lags 0–3	1.0028	1.0008	1.0047	0.78	1.0091	1.0069	1.0113	0.41
Lags 0–4	1.0041	1.0020	1.0063	0.70	1.0086	1.0063	1.0110	0.47
> 50% Black neighborhoods								
Lag 0	1.0099	1.0084	1.0114	0.20	1.0164	1.0147	1.0180	0.05
Lags 0–1	1.0108	1.0091	1.0126	0.22	1.0191	1.0172	1.0210	0.05
Lags 0–2	1.0084	1.0064	1.0103	0.40	1.0212	1.0190	1.0234	0.05
Lags 0–3	1.0081	1.0060	1.0103	0.46	1.0226	1.0202	1.0250	0.06
Lags 0–4	1.0093	1.0069	1.0116	0.44	1.0230	1.0204	1.0257	0.08
White individuals								
Lag 0	1.0115	1.0103	1.0126	< 0.05	1.0117	1.0104	1.0129	**0.07**
Lags 0–1	1.0106	1.0093	1.0119	0.11	1.0099	1.0084	1.0113	0.17
Lags 0–2	1.0080	1.0066	1.0094	0.27	1.0083	1.0067	1.0099	0.30
Lags 0–3	1.0065	1.0049	1.0081	0.42	1.0064	1.0047	1.0081	0.47
Lags 0–4	1.0065	1.0048	1.0082	0.46	1.0069	1.0050	1.0088	0.47
> 50% White neighborhoods								
Lag 0	1.0049	1.0035	1.0063	0.48	1.0069	1.0054	1.0084	0.37
Lags 0–1	1.0021	1.0006	1.0037	0.79	1.0017	1.0000	1.0034	0.85
Lags 0–2	1.0007	0.9990	1.0025	0.94	0.9996	0.9977	1.0016	0.97
Lags 0–3	0.9989	0.9970	1.0009	0.91	0.9968	0.9947	0.9990	0.77
Lags 0–4	0.9963	0.9942	0.9984	0.73	0.9951	0.9928	0.9975	0.68

**Table:** *Original and corrected estimates for main effects from Supplement F.*

	Old estimate				New estimate			
Heat day	Estimate	2.5%	97.5%	p	Estimate	2.5%	97.5%	p
California								
Any day	1.0150	1.0087	1.0214	< 0.05	1.2317	1.2232	1.2403	< 0.05
1st day	1.0166	1.0069	1.0264	< 0.05	1.2076	1.1952	1.2202	< 0.05
2nd day	1.0024	0.9906	1.0142	0.69	1.0891	1.0753	1.1030	0.19
3rd day	1.0117	0.9973	1.0262	0.11	1.1726	1.1547	1.1908	**< 0.05**
Florida								
Any day	1.0042	0.9956	1.0129	0.34	1.0208	1.0111	1.0306	0.67
1st day	1.0066	0.9968	1.0164	0.19	1.0338	1.0231	1.0447	0.53
2nd day	0.9954	0.9833	1.0077	0.46	0.9616	0.9490	0.9744	0.56
3rd day	1.0053	0.9898	1.0210	0.51	1.0157	0.9989	1.0328	0.85
Georgia								
Any day	0.9935	0.9658	1.0219	0.65	0.9617	0.9319	0.9924	0.81
1st day	0.9909	0.9589	1.0239	0.58	0.9385	0.9056	0.9726	0.73
2nd day	1.0154	0.9767	1.0557	0.44	1.3822	1.3251	1.4418	0.13
3rd day	0.9768	0.9333	1.0224	0.31	0.7916	0.7537	0.8314	0.35
Illinois								
Any day	0.9945	0.9805	1.0087	0.45	0.9659	0.9511	0.9809	0.66
1st day	1.0089	0.9944	1.0235	0.23	1.1091	1.0921	1.1264	0.19
2nd day	0.9912	0.9740	1.0087	0.32	0.9506	0.9329	0.9685	0.60
3rd day	1.0148	0.9933	1.0368	0.18	1.1250	1.0996	1.1511	0.31
Indiana								
Any day	0.9819	0.9385	1.0273	0.43	0.6862	0.6534	0.7207	0.13
1st day	1.0208	0.9802	1.0631	0.32	1.0518	1.0066	1.0991	0.82
2nd day	1.0022	0.9552	1.0515	0.93	0.8497	0.8069	0.8948	0.54
3rd day	0.9960	0.9364	1.0594	0.90	1.0909	1.0211	1.1655	0.80
Kansas								
Any day	1.0164	0.9618	1.0741	0.56	1.2327	1.1602	1.3096	0.50
1st day	0.9920	0.9349	1.0525	0.79	0.8290	0.7780	0.8835	0.56
2nd day	0.9833	0.9118	1.0604	0.66	0.8378	0.7723	0.9088	0.67
3rd day	0.9584	0.8742	1.0508	0.37	0.7700	0.6972	0.8503	0.61
Massachusetts								
Any day	1.0090	0.9933	1.0249	0.26	1.0228	1.0055	1.0404	0.80
1st day	1.0293	1.0141	1.0447	< 0.05	1.2644	1.2444	1.2847	< 0.05
2nd day	1.0068	0.9888	1.0251	0.46	1.0798	1.0591	1.1009	0.44
3rd day	0.9766	0.9544	0.9993	< 0.05	0.8990	0.8771	0.9215	**0.40**
Michigan								
Any day	0.9947	0.9771	1.0126	0.56	0.9648	0.9464	0.9835	0.72
1st day	1.0077	0.9905	1.0251	0.38	1.1096	1.0892	1.1304	0.27
2nd day	1.0106	0.9898	1.0319	0.32	1.2030	1.1763	1.2302	0.11
3rd day	1.0044	0.9786	1.0309	0.74	0.9753	0.9486	1.0028	0.86
Missouri								
Any day	1.0208	0.9980	1.0441	0.07	1.0411	1.0155	1.0673	0.75
1st day	1.0145	0.9912	1.0384	0.22	1.0484	1.0227	1.0747	0.71
2nd day	1.0190	0.9908	1.0480	0.19	1.1461	1.1121	1.1812	0.37
3rd day	1.0287	0.9959	1.0625	0.09	1.2878	1.2440	1.3332	0.15
New Hampshire								
Any day	1.0007	0.9561	1.0474	0.98	0.8465	0.8054	0.8897	0.51
1st day	0.9961	0.9545	1.0395	0.86	0.8501	0.8122	0.8899	0.49
2nd day	1.0596	1.0053	1.1167	< 0.05	1.5480	1.4627	1.6383	**0.13**
3rd day	0.9796	0.9140	1.0499	0.56	0.8454	0.7847	0.9108	0.66
New Jersey								
Any day	0.9732	0.9510	0.9959	< 0.05	0.7422	0.7236	0.7612	**< 0.05**
1st day	0.9871	0.9649	1.0099	0.27	0.8486	0.8281	0.8695	0.19
2nd day	1.0011	0.9740	1.0289	0.94	1.0317	1.0019	1.0623	0.83
3rd day	1.0080	0.9756	1.0415	0.63	1.1852	1.1442	1.2277	0.34
Ohio								
Any day	1.0083	0.9917	1.0251	0.33	1.1022	1.0827	1.1220	0.28
1st day	1.0096	0.9938	1.0257	0.23	1.1219	1.1030	1.1411	0.18
2nd day	1.0104	0.9919	1.0293	0.27	1.0785	1.0573	1.1002	0.46
3rd day	1.0015	0.9789	1.0246	0.90	1.0670	1.0411	1.0937	0.61
Texas								
Any day	1.0015	0.9933	1.0097	0.73	0.9984	0.9894	1.0075	0.97
1st day	0.9931	0.9827	1.0035	0.19	0.9285	0.9181	0.9391	0.20
2nd day	1.0053	0.9925	1.0183	0.42	1.0396	1.0253	1.0540	0.58
3rd day	1.0240	1.0086	1.0398	< 0.05	1.2998	1.2787	1.3213	< 0.05

**Table:** *Original and corrected estimates for interactions from Supplement F.*

	Old estimate				New estimate			
Heat day	Estimate	2.5%	97.5%	p	Estimate	2.5%	97.5%	p
California								
Any day	1.0239	0.9882	1.0607	0.19	1.3192	1.2689	1.3716	0.16
1st day	0.9851	0.9306	1.0429	0.61	0.8294	0.7802	0.8817	0.55
2nd day	1.0389	0.9679	1.1151	0.29	1.5664	1.4515	1.6905	0.25
3rd day	1.0402	0.9549	1.1331	0.37	1.4147	1.2893	1.5523	0.46
Florida								
Any day	1.0089	0.9381	1.0849	0.81	1.0615	0.9789	1.1510	0.89
1st day	0.8867	0.7949	0.9891	< 0.05	0.2448	0.2178	0.2751	< 0.05
2nd day	0.9972	0.8718	1.1407	0.97	0.8372	0.7250	0.9667	0.81
3rd day	1.1187	0.9424	1.3279	0.20	3.5029	2.9067	4.2214	0.19
Georgia								
Any day	1.0635	0.8713	1.2982	0.54	2.3353	1.8653	2.9237	0.46
1st day	1.0903	0.7976	1.4905	0.59	3.1935	2.2622	4.5081	0.51
2nd day	0.8493	0.5684	1.2689	0.43	0.0714	0.0467	0.1092	0.22
3rd day	1.3865	0.8795	2.1856	0.16	43.2690	26.2281	71.3815	0.14
Illinois								
Any day	1.0152	0.9739	1.0583	0.48	1.2365	1.1810	1.2946	0.37
1st day	0.9766	0.9163	1.0409	0.47	0.9484	0.8853	1.0161	0.88
2nd day	1.0812	1.0024	1.1663	< 0.05	2.3017	2.1222	2.4964	< 0.05
3rd day	0.9878	0.8984	1.0860	0.80	0.7562	0.6837	0.8365	0.59
Indiana								
Any day	1.0256	0.8980	1.1713	0.71	1.2758	1.1030	1.4755	0.74
1st day	0.9508	0.7765	1.1641	0.62	0.8328	0.6673	1.0394	0.87
2nd day	1.0904	0.8633	1.3773	0.47	3.1247	2.4260	4.0246	0.38
3rd day	1.0593	0.8005	1.4017	0.69	0.7652	0.5657	1.0351	0.86
Kansas								
Any day	0.9867	0.7948	1.2248	0.90	0.5135	0.4006	0.6583	0.60
1st day	1.0590	0.7564	1.4827	0.74	1.4871	1.0361	2.1343	0.83
2nd day	0.7153	0.4172	1.2265	0.22	0.0206	0.0117	0.0365	0.18
3rd day	0.6994	0.3742	1.3071	0.26	0.0222	0.0115	0.0430	0.26
Massachusetts								
Any day	1.0336	0.9793	1.0909	0.23	1.6045	1.5096	1.7055	0.13
1st day	1.0332	0.9530	1.1201	0.43	1.5079	1.3818	1.6455	0.36
2nd day	1.0112	0.9175	1.1144	0.82	1.2129	1.0928	1.3462	0.72
3rd day	1.0376	0.9195	1.1708	0.55	1.0991	0.9656	1.2511	0.89
Michigan								
Any day	0.9166	0.8583	0.9788	< 0.05	0.4083	0.3801	0.4386	< 0.05
1st day	0.9491	0.8608	1.0464	0.29	0.6647	0.5979	0.7389	0.45
2nd day	0.9356	0.8298	1.0550	0.28	0.3779	0.3321	0.4300	0.14
3rd day	0.9029	0.7768	1.0493	0.18	0.4820	0.4108	0.5655	0.37
Missouri								
Any day	1.0474	0.9477	1.1576	0.36	2.1684	1.9404	2.4231	0.17
1st day	1.0663	0.9074	1.2532	0.44	2.2724	1.9092	2.7047	0.36
2nd day	1.0331	0.8472	1.2597	0.75	2.5343	2.0453	3.1401	0.40
3rd day	1.0194	0.8148	1.2753	0.87	2.1669	1.6973	2.7665	0.53
New Hampshire								
Any day	0.7292	0.4301	1.2364	0.24	0.0331	0.0184	0.0596	0.26
1st day	1.1650	0.5956	2.2786	0.66	0.6494	0.3223	1.3085	0.90
2nd day	0.2818	0.0671	1.1839	0.08	0.0000	0.0000	0.0002	0.18
3rd day	0.7075	0.1639	3.0548	0.64	15.2258	2.9415	78.8129	0.75
New Jersey								
Any day	1.0203	0.9514	1.0942	0.57	0.9928	0.9172	1.0747	0.99
1st day	1.1206	1.0069	1.2471	< 0.05	2.2716	2.0244	2.5489	**0.16**
2nd day	0.9623	0.8421	1.0996	0.57	0.5515	0.4784	0.6358	0.41
3rd day	0.9680	0.8248	1.1361	0.69	0.8477	0.7117	1.0096	0.85
Ohio								
Any day	1.0632	0.9809	1.1524	0.14	1.9883	1.8221	2.1698	0.12
1st day	1.0894	0.9608	1.2353	0.18	2.9294	2.5535	3.3606	0.13
2nd day	1.1124	0.9623	1.2860	0.15	2.5414	2.1701	2.9762	0.25
3rd day	0.8973	0.7475	1.0771	0.24	0.3504	0.2876	0.4270	0.30
Texas								
Any day	1.0671	0.9958	1.1435	0.07	1.7130	1.5864	1.8497	0.17
1st day	1.0600	0.9544	1.1772	0.28	2.0693	1.8455	2.3202	0.21
2nd day	0.9206	0.8023	1.0563	0.24	0.3139	0.2708	0.3637	0.12
3rd day	1.1452	0.9800	1.3382	0.09	2.3357	1.9744	2.7630	0.32

**Table:** *Original and corrected estimates for main effects from Supplement G.*

	Old estimate				New estimate			
Exposure	Estimate	2.5%	97.5%	p	Estimate	2.5%	97.5%	p
California								
Lag 0	1.0116	1.0113	1.0119	< 0.05	1.0051	1.0047	1.0054	< 0.05
Lags 0–1	1.0131	1.0128	1.0135	< 0.05	1.0058	1.0055	1.0062	< 0.05
Lags 0–2	1.0136	1.0132	1.0139	< 0.05	1.0055	1.0051	1.0059	< 0.05
Lags 0–3	1.0146	1.0142	1.0149	< 0.05	1.0053	1.0049	1.0057	< 0.05
Lags 0–4	1.0163	1.0160	1.0167	< 0.05	1.0055	1.0051	1.0059	< 0.05
Florida								
Lag 0	0.9980	0.9975	0.9984	0.41	1.0018	1.0012	1.0023	0.52
Lags 0–1	0.9967	0.9962	0.9972	0.23	1.0013	1.0007	1.0019	0.67
Lags 0–2	0.9963	0.9957	0.9969	0.23	1.0019	1.0013	1.0026	0.57
Lags 0–3	0.9943	0.9937	0.9950	0.09	1.0020	1.0013	1.0027	0.59
Lags 0–4	0.9920	0.9913	0.9927	< 0.05	1.0021	1.0013	1.0029	**0.60**
Georgia								
Lag 0	0.9896	0.9885	0.9907	0.07	1.0006	0.9994	1.0018	0.92
Lags 0–1	0.9831	0.9819	0.9844	< 0.05	0.9993	0.9979	1.0007	**0.92**
Lags 0–2	0.9856	0.9842	0.9870	< 0.05	1.0089	1.0073	1.0105	**0.27**
Lags 0–3	0.9829	0.9814	0.9844	< 0.05	1.0082	1.0065	1.0099	**0.35**
Lags 0–4	0.9818	0.9802	0.9834	< 0.05	1.0085	1.0066	1.0104	**0.37**
Illinois								
Lag 0	1.0043	1.0039	1.0048	0.07	1.0091	1.0086	1.0096	**< 0.05**
Lags 0–1	1.0051	1.0045	1.0056	0.06	1.0118	1.0112	1.0124	**< 0.05**
Lags 0–2	1.0025	1.0019	1.0031	0.40	1.0114	1.0107	1.0121	**< 0.05**
Lags 0–3	0.9992	0.9986	0.9999	0.82	1.0098	1.0091	1.0105	**< 0.05**
Lags 0–4	0.9951	0.9944	0.9958	0.16	1.0071	1.0063	1.0078	0.08
Indiana								
Lag 0	0.9706	0.9695	0.9718	< 0.05	0.9956	0.9942	0.9970	**0.53**
Lags 0–1	0.9639	0.9626	0.9653	< 0.05	0.9991	0.9975	1.0007	**0.91**
Lags 0–2	0.9607	0.9592	0.9621	< 0.05	1.0012	0.9995	1.0030	**0.89**
Lags 0–3	0.9583	0.9567	0.9599	< 0.05	1.0033	1.0014	1.0051	**0.74**
Lags 0–4	0.9601	0.9583	0.9619	< 0.05	1.0101	1.0080	1.0121	**0.34**
Kansas								
Lag 0	1.0279	1.0258	1.0300	< 0.05	1.0405	1.0381	1.0428	< 0.05
Lags 0–1	1.0121	1.0097	1.0144	0.31	1.0243	1.0217	1.0270	**0.07**
Lags 0–2	0.9969	0.9944	0.9995	0.81	1.0084	1.0056	1.0113	**0.56**
Lags 0–3	0.9829	0.9801	0.9856	0.22	0.9970	0.9939	1.0001	**0.85**
Lags 0–4	0.9739	0.9710	0.9769	0.09	0.9914	0.9881	0.9948	**0.62**
Massachusetts								
Lag 0	1.0099	1.0093	1.0104	< 0.05	1.0087	1.0081	1.0093	< 0.05
Lags 0–1	1.0166	1.0160	1.0173	< 0.05	1.0162	1.0154	1.0169	< 0.05
Lags 0–2	1.0149	1.0142	1.0157	< 0.05	1.0162	1.0154	1.0170	< 0.05
Lags 0–3	1.0134	1.0126	1.0142	< 0.05	1.0151	1.0142	1.0161	< 0.05
Lags 0–4	1.0106	1.0097	1.0114	< 0.05	1.0118	1.0108	1.0128	< 0.05
Michigan								
Lag 0	1.0049	1.0044	1.0055	0.09	1.0121	1.0114	1.0127	**< 0.05**
Lags 0–1	1.0093	1.0087	1.0100	< 0.05	1.0191	1.0184	1.0199	< 0.05
Lags 0–2	1.0072	1.0064	1.0079	0.06	1.0175	1.0166	1.0183	**< 0.05**
Lags 0–3	1.0040	1.0032	1.0048	0.33	1.0128	1.0119	1.0137	**< 0.05**
Lags 0–4	1.0024	1.0015	1.0033	0.59	1.0096	1.0086	1.0106	0.06
Missouri								
Lag 0	1.0174	1.0165	1.0183	< 0.05	1.0112	1.0102	1.0121	< 0.05
Lags 0–1	1.0190	1.0180	1.0200	< 0.05	1.0123	1.0111	1.0134	< 0.05
Lags 0–2	1.0177	1.0166	1.0188	< 0.05	1.0116	1.0104	1.0128	**0.06**
Lags 0–3	1.0145	1.0133	1.0157	< 0.05	1.0080	1.0067	1.0094	**0.23**
Lags 0–4	1.0078	1.0065	1.0091	0.23	1.0034	1.0020	1.0048	0.64
New Hampshire								
Lag 0	1.0243	1.0222	1.0265	< 0.05	1.0283	1.0259	1.0307	< 0.05
Lags 0–1	1.0183	1.0158	1.0209	0.15	1.0229	1.0201	1.0258	0.11
Lags 0–2	1.0098	1.0070	1.0127	0.50	1.0108	1.0077	1.0140	0.5
Lags 0–3	0.9932	0.9902	0.9963	0.67	0.9889	0.9855	0.9924	0.53
Lags 0–4	0.9899	0.9866	0.9932	0.55	0.9857	0.9819	0.9895	0.46
New Jersey								
Lag 0	1.0152	1.0146	1.0159	< 0.05	1.0106	1.0099	1.0113	< 0.05
Lags 0–1	1.0152	1.0144	1.0159	< 0.05	1.0138	1.0130	1.0146	< 0.05
Lags 0–2	1.0121	1.0112	1.0129	< 0.05	1.0134	1.0125	1.0144	< 0.05
Lags 0–3	1.0095	1.0086	1.0105	< 0.05	1.0129	1.0118	1.0139	< 0.05
Lags 0–4	1.0064	1.0054	1.0074	0.22	1.0113	1.0101	1.0124	0.05
Ohio								
Lag 0	1.0088	1.0083	1.0094	< 0.05	1.0077	1.0071	1.0083	< 0.05
Lags 0–1	1.0096	1.0090	1.0102	< 0.05	1.0091	1.0084	1.0098	< 0.05
Lags 0–2	1.0058	1.0051	1.0065	0.09	1.0041	1.0033	1.0048	0.29
Lags 0–3	1.0030	1.0022	1.0037	0.43	0.9996	0.9988	1.0005	0.93
Lags 0–4	1.0008	1.0000	1.0016	0.85	0.9956	0.9947	0.9965	0.34
Texas								
Lag 0	1.0087	1.0082	1.0092	< 0.05	1.0107	1.0102	1.0112	< 0.05
Lags 0–1	1.0096	1.0091	1.0102	< 0.05	1.0126	1.0121	1.0132	< 0.05
Lags 0–2	1.0049	1.0043	1.0055	0.09	1.0087	1.0081	1.0093	**< 0.05**
Lags 0–3	1.0012	1.0006	1.0019	0.69	1.0059	1.0053	1.0066	0.09
Lags 0–4	0.9999	0.9993	1.0006	0.98	1.0062	1.0055	1.0070	0.10

**Table:** *Original and corrected estimates for interactions from Supplement G.*

	Old estimate				New estimate			
Exposure	Estimate	2.5%	97.5%	p	Estimate	2.5%	97.5%	p
California								
Lag 0	1.0131	1.0115	1.0147	0.12	1.0096	1.0079	1.0114	0.29
Lags 0–1	1.0063	1.0046	1.0081	0.49	1.0009	0.9989	1.0029	0.93
Lags 0–2	1.0064	1.0045	1.0084	0.52	1.0018	0.9996	1.0039	0.87
Lags 0–3	1.0076	1.0055	1.0097	0.48	1.0018	0.9995	1.0041	0.88
Lags 0–4	1.0114	1.0092	1.0137	0.32	1.0024	0.9999	1.0049	0.85
Florida								
Lag 0	1.0115	1.0065	1.0165	0.65	1.0098	1.0044	1.0152	0.72
Lags 0–1	1.0002	0.9946	1.0058	0.99	0.9966	0.9905	1.0027	0.91
Lags 0–2	0.9885	0.9824	0.9947	0.72	0.9857	0.9789	0.9925	0.68
Lags 0–3	0.9952	0.9886	1.0019	0.89	0.9890	0.9817	0.9964	0.77
Lags 0–4	1.0230	1.0159	1.0302	0.52	1.0164	1.0085	1.0244	0.68
Georgia								
Lag 0	1.0284	1.0187	1.0383	0.56	1.0402	1.0292	1.0513	0.47
Lags 0–1	1.0243	1.0134	1.0353	0.66	1.0457	1.0331	1.0585	0.47
Lags 0–2	1.0373	1.0251	1.0496	0.54	1.0619	1.0476	1.0764	0.39
Lags 0–3	1.0495	1.0361	1.0631	0.46	1.0869	1.0710	1.1030	0.27
Lags 0–4	1.0644	1.0499	1.0792	0.37	1.1123	1.0947	1.1301	0.19
Illinois								
Lag 0	1.0071	1.0054	1.0089	0.42	1.0145	1.0125	1.0164	0.14
Lags 0–1	1.0075	1.0055	1.0095	0.46	1.0157	1.0135	1.0180	0.16
Lags 0–2	1.0081	1.0058	1.0104	0.48	1.0193	1.0168	1.0218	0.13
Lags 0–3	1.0058	1.0033	1.0083	0.65	1.0164	1.0136	1.0192	0.25
Lags 0–4	1.0031	1.0004	1.0059	0.82	1.0126	1.0096	1.0157	0.42
Indiana								
Lag 0	1.0775	1.0720	1.0830	< 0.05	1.0856	1.0797	1.0917	< 0.05
Lags 0–1	1.0781	1.0719	1.0843	< 0.05	1.0804	1.0737	1.0872	< 0.05
Lags 0–2	1.0657	1.0589	1.0725	0.05	1.0595	1.0521	1.0669	0.1
Lags 0–3	1.0581	1.0507	1.0655	0.11	1.0484	1.0405	1.0564	0.22
Lags 0–4	1.0586	1.0506	1.0667	0.14	1.0490	1.0405	1.0577	0.25
Kansas								
Lag 0	0.9561	0.9444	0.9680	0.48	0.9625	0.9495	0.9756	0.58
Lags 0–1	0.9770	0.9634	0.9908	0.74	0.9981	0.9827	1.0138	0.98
Lags 0–2	1.0025	0.9873	1.0180	0.97	1.0254	1.0081	1.0431	0.77
Lags 0–3	1.0396	1.0225	1.0570	0.65	1.0873	1.0673	1.1077	0.38
Lags 0–4	1.0649	1.0460	1.0840	0.49	1.1367	1.1140	1.1598	0.21
Massachusetts								
Lag 0	0.9984	0.9960	1.0009	0.90	0.9968	0.9941	0.9995	0.82
Lags 0–1	1.0046	1.0017	1.0075	0.76	1.0036	1.0004	1.0068	0.83
Lags 0–2	0.9955	0.9922	0.9988	0.79	0.9970	0.9933	1.0007	0.87
Lags 0–3	0.9887	0.9851	0.9923	0.54	0.9894	0.9853	0.9935	0.61
Lags 0–4	0.9839	0.9800	0.9879	0.43	0.9851	0.9807	0.9896	0.52
Michigan								
Lag 0	1.0121	1.0093	1.0149	0.39	1.0197	1.0167	1.0228	0.2
Lags 0–1	1.0026	0.9995	1.0057	0.87	1.0094	1.0060	1.0129	0.59
Lags 0–2	1.0060	1.0024	1.0095	0.74	1.0118	1.0079	1.0157	0.55
Lags 0–3	1.0059	1.0020	1.0098	0.77	1.0105	1.0062	1.0147	0.63
Lags 0–4	1.0052	1.0010	1.0095	0.81	1.0127	1.0081	1.0174	0.59
Missouri								
Lag 0	1.0262	1.0206	1.0319	0.35	1.0412	1.0349	1.0475	0.19
Lags 0–1	1.0195	1.0132	1.0258	0.54	1.0253	1.0183	1.0324	0.47
Lags 0–2	1.0311	1.0241	1.0381	0.38	1.0473	1.0394	1.0553	0.23
Lags 0–3	1.0159	1.0084	1.0235	0.68	1.0407	1.0321	1.0494	0.35
Lags 0–4	0.9988	0.9909	1.0069	0.98	1.0288	1.0196	1.0381	0.54
New Hampshire								
Lag 0	1.0593	1.0313	1.0881	0.67	1.1049	1.0732	1.1375	0.5
Lags 0–1	0.9618	0.9318	0.9928	0.81	0.9531	0.9205	0.9868	0.79
Lags 0–2	0.9259	0.8930	0.9601	0.68	0.8824	0.8480	0.9182	0.54
Lags 0–3	0.8920	0.8570	0.9284	0.58	0.8629	0.8255	0.9020	0.51
Lags 0–4	0.7826	0.7483	0.8185	0.28	0.7777	0.7403	0.8169	0.32
New Jersey								
Lag 0	0.9974	0.9950	0.9999	0.84	0.9950	0.9924	0.9977	0.71
Lags 0–1	0.9943	0.9915	0.9972	0.69	0.9890	0.9859	0.9920	0.48
Lags 0–2	0.9920	0.9888	0.9952	0.63	0.9898	0.9863	0.9933	0.57
Lags 0–3	0.9906	0.9870	0.9943	0.61	0.9920	0.9881	0.9960	0.69
Lags 0–4	0.9878	0.9839	0.9918	0.55	0.9899	0.9855	0.9942	0.65
Ohio								
Lag 0	0.9978	0.9942	1.0013	0.90	0.9990	0.9952	1.0028	0.96
Lags 0–1	0.9976	0.9937	1.0017	0.91	1.0002	0.9959	1.0045	0.99
Lags 0–2	0.9937	0.9893	0.9982	0.78	0.9961	0.9913	1.0009	0.87
Lags 0–3	0.9977	0.9928	1.0027	0.93	1.0005	0.9951	1.0058	0.99
Lags 0–4	1.0000	0.9946	1.0054	1.00	1.0018	0.9960	1.0077	0.95
Texas								
Lag 0	0.9901	0.9853	0.9949	0.69	0.9684	0.9634	0.9735	0.23
Lags 0–1	0.9836	0.9782	0.9891	0.56	0.9667	0.9609	0.9725	0.27
Lags 0–2	0.9861	0.9800	0.9921	0.65	0.9765	0.9699	0.9831	0.49
Lags 0–3	0.9957	0.9891	1.0023	0.90	0.9855	0.9783	0.9928	0.7
Lags 0–4	1.0081	1.0010	1.0154	0.82	0.9958	0.9879	1.0037	0.92

**Table:** *Original and corrected estimates for main effects from Supplement H.*

	Old estimate				New estimate			
Heat day	Estimate	2.5%	97.5%	p	Estimate	2.5%	97.5%	p
2000								
Any day	0.9485	0.8922	1.0084	0.09	0.4763	0.4465	0.5081	**< 0.05**
1st day	1.0005	0.9456	1.0587	0.99	0.8542	0.8043	0.9073	0.61
2nd day	0.9966	0.9280	1.0702	0.92	0.7894	0.7312	0.8523	0.55
3rd day	0.9807	0.8987	1.0703	0.66	1.0744	0.9767	1.1818	0.88
2001								
Any day	0.9800	0.9237	1.0397	0.50	0.6833	0.6401	0.7294	0.25
1st day	1.0296	0.9751	1.0872	0.29	1.0925	1.0298	1.1589	0.77
2nd day	1.0372	0.9683	1.1110	0.30	1.9251	1.7879	2.0728	0.08
3rd day	0.9641	0.8776	1.0591	0.45	0.8348	0.7553	0.9226	0.72
2002								
Any day	0.9943	0.9341	1.0585	0.86	1.0278	0.9618	1.0983	0.94
1st day	0.9934	0.9368	1.0534	0.82	0.9970	0.9345	1.0637	0.99
2nd day	0.9974	0.9289	1.0709	0.94	0.8534	0.7901	0.9219	0.69
3rd day	0.9641	0.8776	1.0591	0.45	0.5669	0.5068	0.6343	0.32
2003								
Any day	0.9973	0.9315	1.0677	0.94	0.9187	0.8514	0.9912	0.83
1st day	1.0660	1.0017	1.1344	< 0.05	1.6054	1.5012	1.7169	**0.17**
2nd day	0.9674	0.8944	1.0463	0.41	0.6832	0.6288	0.7423	0.37
3rd day	0.9424	0.8576	1.0355	0.22	0.4967	0.4496	0.5488	0.17
2004								
Any day	1.0521	1.0106	1.0953	< 0.05	1.4140	1.3538	1.4770	**0.12**
1st day	1.0301	0.9911	1.0706	0.13	1.1508	1.1043	1.1993	0.50
2nd day	1.0119	0.9655	1.0606	0.62	1.3339	1.2687	1.4025	0.26
3rd day	1.0559	0.9986	1.1164	0.06	1.7391	1.6377	1.8468	0.07
2005								
Any day	0.9626	0.9226	1.0043	0.08	0.5985	0.5712	0.6271	**< 0.05**
1st day	0.9893	0.9503	1.0299	0.60	0.9058	0.8673	0.9459	0.65
2nd day	0.9827	0.9368	1.0309	0.47	0.6965	0.6617	0.7331	0.17
3rd day	0.9788	0.9238	1.0370	0.47	0.9771	0.9176	1.0405	0.94
2006								
Any day	1.0057	0.9631	1.0502	0.80	1.1723	1.1160	1.2314	0.53
1st day	1.0389	0.9929	1.0871	0.10	1.9436	1.8524	2.0393	**< 0.05**
2nd day	1.0106	0.9600	1.0639	0.69	1.1397	1.0798	1.2029	0.63
3rd day	0.9787	0.9237	1.0370	0.47	0.9307	0.8750	0.9900	0.82
2007								
Any day	1.0045	0.9928	1.0164	0.45	1.1170	1.1022	1.1320	0.10
1st day	1.0103	0.9953	1.0256	0.18	1.0935	1.0760	1.1113	0.28
2nd day	0.9876	0.9695	1.0060	0.18	0.9083	0.8905	0.9265	0.34
3rd day	1.0092	0.9868	1.0322	0.42	1.2268	1.1974	1.2568	0.10
2008								
Any day	1.0028	0.9919	1.0139	0.61	1.0166	1.0043	1.0290	0.79
1st day	0.9990	0.9845	1.0136	0.89	0.9874	0.9719	1.0031	0.87
2nd day	1.0150	0.9978	1.0326	0.09	1.1603	1.1389	1.1821	0.12
3rd day	1.0110	0.9904	1.0321	0.30	1.1741	1.1483	1.2005	0.16
2009								
Any day	1.0017	0.9924	1.0110	0.73	1.0548	1.0440	1.0658	0.31
1st day	1.0093	0.9964	1.0223	0.16	1.0897	1.0749	1.1048	0.22
2nd day	0.9969	0.9814	1.0126	0.70	1.0539	1.0363	1.0717	0.54
3rd day	0.9867	0.9670	1.0069	0.19	0.8997	0.8804	0.9194	0.34
2010								
Any day	1.0260	1.0159	1.0361	< 0.05	1.1450	1.1323	1.1578	< 0.05
1st day	1.0075	0.9939	1.0212	0.28	0.9894	0.9751	1.0041	0.89
2nd day	1.0171	1.0005	1.0340	< 0.05	1.1348	1.1147	1.1552	**0.16**
3rd day	1.0312	1.0109	1.0519	< 0.05	1.2961	1.2684	1.3245	< 0.05
2011								
Any day	1.0004	0.9906	1.0103	0.94	1.1202	1.1079	1.1326	**< 0.05**
1st day	1.0148	1.0011	1.0288	< 0.05	1.2305	1.2127	1.2487	< 0.05
2nd day	0.9869	0.9705	1.0036	0.12	0.9523	0.9354	0.9696	0.59
3rd day	1.0042	0.9838	1.0250	0.69	1.0736	1.0502	1.0975	0.53
2012								
Any day	1.0305	1.0205	1.0406	< 0.05	1.2161	1.2029	1.2295	< 0.05
1st day	1.0189	1.0055	1.0325	< 0.05	1.1252	1.1093	1.1413	**0.10**
2nd day	1.0180	1.0016	1.0347	< 0.05	1.1157	1.0964	1.1353	**0.22**
3rd day	1.0237	1.0034	1.0444	< 0.05	1.2335	1.2073	1.2604	**0.06**
2013								
Any day	1.0168	1.0071	1.0266	< 0.05	1.2231	1.2104	1.2360	< 0.05
1st day	1.0043	0.9908	1.0181	0.53	1.0971	1.0812	1.1133	0.21
2nd day	1.0108	0.9939	1.0279	0.21	1.1586	1.1380	1.1797	0.11
3rd day	1.0225	1.0023	1.0431	< 0.05	1.2665	1.2398	1.2939	< 0.05
2014								
Any day	0.9986	0.9882	1.0091	0.79	1.0207	1.0088	1.0327	0.73
1st day	0.9970	0.9829	1.0114	0.68	0.9818	0.9669	0.9970	0.82
2nd day	0.9953	0.9782	1.0127	0.60	0.9519	0.9342	0.9699	0.61
3rd day	1.0077	0.9858	1.0301	0.49	1.0709	1.0458	1.0966	0.57
2015								
Any day	1.0051	0.9947	1.0156	0.33	1.2467	1.2328	1.2607	**< 0.05**
1st day	1.0071	0.9930	1.0214	0.33	1.1541	1.1368	1.1717	0.06
2nd day	1.0177	1.0003	1.0354	< 0.05	1.3149	1.2909	1.3393	< 0.05
3rd day	1.0173	0.9963	1.0388	0.11	1.3036	1.2747	1.3332	**< 0.05**
2016								
Any day	1.0296	1.0186	1.0406	< 0.05	1.1412	1.1278	1.1549	< 0.05
1st day	1.0277	1.0127	1.0430	< 0.05	1.1876	1.1688	1.2066	< 0.05
2nd day	1.0295	1.0112	1.0482	< 0.05	1.2437	1.2199	1.2681	< 0.05
3rd day	1.0230	1.0007	1.0457	< 0.05	1.0712	1.0462	1.0968	**0.57**

*Original and corrected estimates for interactions from Supplement H.*

**Table:** 

	Old estimate				New estimate			
Heat day	Estimate	2.5%	97.5%	p	Estimate	2.5%	97.5%	p
2000								
Any day	0.9443	0.7704	1.1574	0.58	0.4969	0.4010	0.6156	0.52
1st day	1.1722	0.8804	1.5607	0.28	2.9312	2.1560	3.9851	0.49
2nd day	1.0136	0.7107	1.4456	0.94	1.0813	0.7442	1.5712	0.97
3rd day	0.6749	0.4151	1.0972	0.11	0.0090	0.0054	0.0148	0.07
2001								
Any day	0.9519	0.7836	1.1562	0.62	0.5014	0.4020	0.6253	0.54
1st day	1.0473	0.7939	1.3817	0.74	2.9003	2.1418	3.9274	0.49
2nd day	0.7789	0.5235	1.1589	0.22	0.0758	0.0497	0.1156	0.23
3rd day	0.7273	0.4233	1.2495	0.25	0.0054	0.0031	0.0094	0.07
2002								
Any day	1.0534	0.8531	1.3009	0.63	2.8301	2.2310	3.5900	0.39
1st day	0.8775	0.6143	1.2534	0.47	0.3633	0.2439	0.5413	0.62
2nd day	0.6871	0.4391	1.0749	0.10	0.0174	0.0106	0.0284	0.11
3rd day	0.7273	0.4233	1.2495	0.25	69.4835	38.8563	124.2519	0.15
2003								
Any day	1.1611	0.9524	1.4156	0.14	11.6884	9.3215	14.6564	**< 0.05**
1st day	1.1348	0.8162	1.5778	0.45	9.7846	6.8295	14.0183	0.21
2nd day	0.9508	0.6196	1.4588	0.82	0.9024	0.5774	1.4103	0.96
3rd day	1.8368	1.2226	2.7594	< 0.05	139.0756	91.0386	212.4595	< 0.05
2004								
Any day	1.0272	0.9058	1.1649	0.68	1.2190	1.0593	1.4027	0.78
1st day	1.1850	0.9872	1.4224	0.07	8.2959	6.7905	10.1350	< 0.05
2nd day	0.9205	0.7311	1.1589	0.48	0.2940	0.2298	0.3760	0.33
3rd day	0.8037	0.5995	1.0774	0.14	0.0853	0.0623	0.1168	0.12
2005								
Any day	1.1008	0.9672	1.2528	0.15	4.3792	3.7878	5.0630	**< 0.05**
1st day	1.0888	0.8956	1.3237	0.39	3.6774	2.9680	4.5565	0.23
2nd day	1.0895	0.8674	1.3686	0.46	5.1398	4.0145	6.5806	0.19
3rd day	1.2123	0.9225	1.5930	0.17	4.0832	3.0138	5.5322	0.36
2006								
Any day	1.0740	0.9331	1.2362	0.32	0.9767	0.8337	1.1441	0.98
1st day	1.0997	0.8785	1.3766	0.41	0.9290	0.7339	1.1759	0.95
2nd day	0.9565	0.7413	1.2342	0.73	0.6484	0.4960	0.8477	0.75
3rd day	1.0563	0.7933	1.4066	0.71	0.6745	0.4949	0.9193	0.80
2007								
Any day	0.9811	0.9142	1.0528	0.60	0.9392	0.8684	1.0158	0.88
1st day	1.0253	0.9251	1.1362	0.63	1.3820	1.2352	1.5462	0.57
2nd day	0.9631	0.8473	1.0947	0.56	0.6048	0.5268	0.6942	0.47
3rd day	0.9373	0.7976	1.1014	0.43	0.6173	0.5195	0.7337	0.58
2008								
Any day	0.9911	0.9321	1.0539	0.78	0.7301	0.6818	0.7819	0.37
1st day	1.0069	0.9164	1.1063	0.89	1.0202	0.9208	1.1304	0.97
2nd day	0.9947	0.8893	1.1125	0.93	0.7201	0.6378	0.8131	0.60
3rd day	0.9543	0.8341	1.0917	0.50	0.4485	0.3883	0.5179	0.27
2009								
Any day	0.9772	0.9251	1.0323	0.41	0.6014	0.5658	0.6392	0.10
1st day	0.9188	0.8427	1.0018	0.05	0.3385	0.3088	0.3711	**< 0.05**
2nd day	1.0168	0.9162	1.1285	0.75	1.2062	1.0793	1.3480	0.74
3rd day	1.1579	1.0202	1.3143	< 0.05	3.3197	2.8967	3.8044	**0.08**
2010								
Any day	1.0593	0.9985	1.1236	0.06	2.0142	1.8878	2.1490	**< 0.05**
1st day	1.0025	0.9122	1.1018	0.96	1.1307	1.0210	1.2522	0.81
2nd day	1.1280	1.0108	1.2589	< 0.05	1.8317	1.6273	2.0618	**0.32**
3rd day	1.0317	0.9023	1.1797	0.65	2.2578	1.9498	2.6145	0.28
2011								
Any day	1.0357	0.9765	1.0986	0.24	1.8924	1.7709	2.0224	0.06
1st day	0.9989	0.9103	1.0960	0.98	1.0720	0.9697	1.1852	0.89
2nd day	1.0005	0.8900	1.1247	0.99	1.2928	1.1393	1.4670	0.69
3rd day	0.9915	0.8611	1.1417	0.91	0.9081	0.7800	1.0574	0.90
2012								
Any day	1.0083	0.9502	1.0700	0.78	0.7972	0.7457	0.8522	0.51
1st day	0.9758	0.8909	1.0688	0.60	0.7121	0.6461	0.7849	0.49
2nd day	0.9859	0.8774	1.1078	0.81	0.7069	0.6248	0.7997	0.58
3rd day	0.9844	0.8542	1.1345	0.83	0.6514	0.5601	0.7575	0.58
2013								
Any day	0.9884	0.9332	1.0469	0.69	1.2316	1.1568	1.3112	0.51
1st day	0.9970	0.9071	1.0958	0.95	1.2096	1.0913	1.3406	0.72
2nd day	0.9923	0.8816	1.1168	0.90	1.5142	1.3335	1.7193	0.52
3rd day	0.9897	0.8625	1.1356	0.88	1.2006	1.0358	1.3917	0.81
2014								
Any day	0.9942	0.9311	1.0616	0.86	0.7803	0.7250	0.8397	0.51
1st day	1.0027	0.9090	1.1060	0.96	1.0170	0.9147	1.1308	0.98
2nd day	0.9296	0.8214	1.0520	0.25	0.4366	0.3818	0.4994	0.23
3rd day	1.0659	0.9128	1.2448	0.42	1.8001	1.5194	2.1326	0.50
2015								
Any day	1.1146	1.0457	1.1881	< 0.05	4.0144	3.7491	4.2986	< 0.05
1st day	1.0730	0.9751	1.1808	0.15	2.6014	2.3470	2.8834	0.07
2nd day	1.1101	0.9852	1.2509	0.09	4.6836	4.1202	5.3239	**< 0.05**
3rd day	1.0207	0.8828	1.1802	0.78	1.1026	0.9431	1.2890	0.90
2016								
Any day	1.0456	0.9784	1.1175	0.19	1.7317	1.6103	1.8622	0.14
1st day	0.9830	0.8883	1.0877	0.74	0.9885	0.8862	1.1027	0.98
2nd day	1.1660	1.0383	1.3095	< 0.05	3.6850	3.2514	4.1764	< 0.05
3rd day	0.9712	0.8292	1.1374	0.72	1.3298	1.1220	1.5762	0.74

**Table:** *Original and corrected estimates for main effects from Supplement I.*

	Old estimate				New estimate			
Exposure	Estimate	2.5%	97.5%	p	Estimate	2.5%	97.5%	p
2000								
Lag 0	0.9216	0.9197	0.9235	< 0.05	0.9833	0.9811	0.9856	**0.16**
Lags 0–1	0.8989	0.8967	0.9012	< 0.05	0.9878	0.9851	0.9906	**0.39**
Lags 0–2	0.8736	0.8711	0.8761	< 0.05	0.9957	0.9925	0.9990	**0.80**
Lags 0–3	0.8453	0.8426	0.8481	< 0.05	0.9959	0.9922	0.9996	**0.83**
Lags 0–4	0.8278	0.8249	0.8308	< 0.05	0.9896	0.9856	0.9937	**0.61**
2001								
Lag 0	1.0651	1.0632	1.0670	< 0.05	1.0342	1.0321	1.0364	< 0.05
Lags 0–1	1.1001	1.0978	1.1025	< 0.05	1.0482	1.0456	1.0508	< 0.05
Lags 0–2	1.1190	1.1164	1.1217	< 0.05	1.0489	1.0459	1.0520	< 0.05
Lags 0–3	1.1407	1.1376	1.1437	< 0.05	1.0535	1.0501	1.0569	< 0.05
Lags 0–4	1.1591	1.1557	1.1625	< 0.05	1.0593	1.0555	1.0630	< 0.05
2002								
Lag 0	0.9930	0.9915	0.9945	0.37	0.9933	0.9916	0.9949	0.43
Lags 0–1	0.9988	0.9970	1.0006	0.90	0.9986	0.9965	1.0006	0.89
Lags 0–2	0.9998	0.9977	1.0019	0.99	0.9963	0.9940	0.9986	0.75
Lags 0–3	0.9972	0.9948	0.9995	0.81	0.9887	0.9862	0.9913	0.40
Lags 0–4	0.9846	0.9820	0.9871	0.24	0.9736	0.9707	0.9764	0.07
2003								
Lag 0	0.9895	0.9877	0.9912	0.23	1.0054	1.0035	1.0074	0.58
Lags 0–1	0.9850	0.9828	0.9871	0.17	1.0099	1.0074	1.0123	0.43
Lags 0–2	0.9590	0.9566	0.9614	< 0.05	0.9940	0.9911	0.9970	**0.69**
Lags 0–3	0.9577	0.9550	0.9604	< 0.05	0.9998	0.9964	1.0032	**0.99**
Lags 0–4	0.9570	0.9540	0.9600	< 0.05	1.0011	0.9973	1.0049	**0.96**
2004								
Lag 0	1.0398	1.0387	1.0410	< 0.05	1.0137	1.0125	1.0149	< 0.05
Lags 0–1	1.0428	1.0414	1.0441	< 0.05	1.0153	1.0139	1.0168	< 0.05
Lags 0–2	1.0410	1.0395	1.0426	< 0.05	1.0153	1.0137	1.0169	**0.06**
Lags 0–3	1.0428	1.0411	1.0445	< 0.05	1.0178	1.0159	1.0196	**0.05**
Lags 0–4	1.0388	1.0369	1.0407	< 0.05	1.0119	1.0100	1.0139	**0.24**
2005								
Lag 0	1.0515	1.0503	1.0527	< 0.05	1.0135	1.0122	1.0149	**0.05**
Lags 0–1	1.0779	1.0764	1.0793	< 0.05	1.0208	1.0192	1.0225	< 0.05
Lags 0–2	1.1114	1.1097	1.1132	< 0.05	1.0290	1.0271	1.0309	< 0.05
Lags 0–3	1.1355	1.1335	1.1375	< 0.05	1.0332	1.0310	1.0353	< 0.05
Lags 0–4	1.1494	1.1472	1.1516	< 0.05	1.0290	1.0267	1.0313	< 0.05
2006								
Lag 0	0.9910	0.9898	0.9921	0.14	1.0108	1.0095	1.0122	0.11
Lags 0–1	0.9900	0.9886	0.9914	0.16	1.0158	1.0142	1.0174	0.05
Lags 0–2	0.9841	0.9825	0.9856	< 0.05	1.0194	1.0175	1.0212	< 0.05
Lags 0–3	0.9782	0.9764	0.9799	< 0.05	1.0202	1.0181	1.0222	**0.05**
Lags 0–4	0.9759	0.9740	0.9778	< 0.05	1.0237	1.0214	1.0260	< 0.05
2007								
Lag 0	1.0041	1.0036	1.0045	0.09	1.0113	1.0108	1.0118	< 0.05
Lags 0–1	0.9993	0.9988	0.9998	0.79	1.0122	1.0116	1.0128	< 0.05
Lags 0–2	0.9944	0.9938	0.9950	0.06	1.0110	1.0103	1.0116	< 0.05
Lags 0–3	0.9914	0.9907	0.9920	< 0.05	1.0092	1.0085	1.0099	< 0.05
Lags 0–4	0.9903	0.9896	0.9910	< 0.05	1.0098	1.0090	1.0106	< 0.05
2008								
Lag 0	0.9552	0.9547	0.9557	< 0.05	1.0062	1.0056	1.0067	< 0.05
Lags 0–1	0.9504	0.9498	0.9509	< 0.05	1.0105	1.0099	1.0112	< 0.05
Lags 0–2	0.9413	0.9407	0.9419	< 0.05	1.0088	1.0081	1.0095	< 0.05
Lags 0–3	0.9319	0.9312	0.9325	< 0.05	1.0077	1.0069	1.0085	**0.06**
Lags 0–4	0.9250	0.9243	0.9257	< 0.05	1.0075	1.0066	1.0084	**0.10**
2009								
Lag 0	1.0319	1.0314	1.0324	< 0.05	1.0067	1.0062	1.0072	< 0.05
Lags 0–1	1.0353	1.0347	1.0358	< 0.05	1.0098	1.0092	1.0104	< 0.05
Lags 0–2	1.0344	1.0338	1.0350	< 0.05	1.0093	1.0087	1.0100	< 0.05
Lags 0–3	1.0346	1.0339	1.0352	< 0.05	1.0085	1.0077	1.0092	< 0.05
Lags 0–4	1.0355	1.0348	1.0362	< 0.05	1.0069	1.0061	1.0077	**0.08**
2010								
Lag 0	1.0268	1.0263	1.0273	< 0.05	1.0102	1.0097	1.0107	< 0.05
Lags 0–1	1.0374	1.0368	1.0379	< 0.05	1.0136	1.0131	1.0142	< 0.05
Lags 0–2	1.0425	1.0419	1.0431	< 0.05	1.0126	1.0120	1.0133	< 0.05
Lags 0–3	1.0442	1.0435	1.0448	< 0.05	1.0113	1.0106	1.0120	< 0.05
Lags 0–4	1.0436	1.0429	1.0443	< 0.05	1.0098	1.0090	1.0105	< 0.05
2011								
Lag 0	1.0092	1.0087	1.0097	< 0.05	1.0071	1.0066	1.0076	< 0.05
Lags 0–1	1.0075	1.0070	1.0081	< 0.05	1.0090	1.0084	1.0096	< 0.05
Lags 0–2	1.0013	1.0007	1.0019	0.68	1.0097	1.0090	1.0104	**< 0.05**
Lags 0–3	0.9960	0.9953	0.9966	0.23	1.0083	1.0076	1.0090	**< 0.05**
Lags 0–4	0.9934	0.9927	0.9941	0.07	1.0077	1.0069	1.0085	0.06
2012								
Lag 0	0.9969	0.9964	0.9974	0.23	1.0062	1.0057	1.0068	**< 0.05**
Lags 0–1	0.9966	0.9961	0.9972	0.23	1.0080	1.0074	1.0086	**< 0.05**
Lags 0–2	0.9914	0.9908	0.9920	< 0.05	1.0043	1.0036	1.0050	**0.22**
Lags 0–3	0.9859	0.9853	0.9866	< 0.05	1.0005	0.9998	1.0012	**0.9**
Lags 0–4	0.9819	0.9813	0.9826	< 0.05	0.9995	0.9987	1.0003	**0.91**
2013								
Lag 0	1.0155	1.0150	1.0160	< 0.05	1.0051	1.0045	1.0056	**0.07**
Lags 0–1	1.0204	1.0198	1.0209	< 0.05	1.0087	1.0081	1.0093	< 0.05
Lags 0–2	1.0264	1.0258	1.0270	< 0.05	1.0078	1.0071	1.0084	< 0.05
Lags 0–3	1.0342	1.0336	1.0349	< 0.05	1.0063	1.0056	1.0071	**0.08**
Lags 0–4	1.0416	1.0409	1.0423	< 0.05	1.0042	1.0034	1.0050	**0.29**
2014								
Lag 0	0.9768	0.9763	0.9773	< 0.05	1.0037	1.0032	1.0043	**0.21**
Lags 0–1	0.9775	0.9769	0.9780	< 0.05	1.0034	1.0028	1.0040	**0.3**
Lags 0–2	0.9756	0.9750	0.9762	< 0.05	1.0025	1.0018	1.0032	**0.48**
Lags 0–3	0.9732	0.9726	0.9738	< 0.05	1.0023	1.0015	1.0030	**0.55**
Lags 0–4	0.9711	0.9705	0.9718	< 0.05	1.0024	1.0016	1.0032	**0.56**
2015								
Lag 0	1.0257	1.0251	1.0262	< 0.05	1.0080	1.0074	1.0086	< 0.05
Lags 0–1	1.0262	1.0256	1.0268	< 0.05	1.0077	1.0070	1.0084	< 0.05
Lags 0–2	1.0248	1.0241	1.0254	< 0.05	1.0070	1.0063	1.0077	**0.06**
Lags 0–3	1.0246	1.0239	1.0253	< 0.05	1.0054	1.0047	1.0062	**0.17**
Lags 0–4	1.0277	1.0269	1.0285	< 0.05	1.0063	1.0055	1.0072	**0.14**
2016								
Lag 0	1.0261	1.0255	1.0267	< 0.05	1.0107	1.0100	1.0113	< 0.05
Lags 0–1	1.0302	1.0295	1.0309	< 0.05	1.0106	1.0099	1.0114	< 0.05
Lags 0–2	1.0344	1.0336	1.0351	< 0.05	1.0116	1.0108	1.0125	< 0.05
Lags 0–3	1.0353	1.0345	1.0361	< 0.05	1.0115	1.0106	1.0124	< 0.05
Lags 0–4	1.0333	1.0324	1.0342	< 0.05	1.0098	1.0088	1.0107	< 0.05

**Table:** *Original and corrected estimates for interactions from Supplement I.*

	Old estimate				New estimate			
Exposure	Estimate	2.5%	97.5%	p	Estimate	2.5%	97.5%	p
2000								
Lag 0	1.0093	1.0003	1.0185	0.84	1.0127	1.0028	1.0227	0.80
Lags 0–1	0.9539	0.9435	0.9644	0.40	0.9506	0.9392	0.9621	0.41
Lags 0–2	0.8877	0.8763	0.8992	0.07	0.8912	0.8785	0.9041	0.12
Lags 0–3	0.8555	0.8431	0.8682	< 0.05	0.8743	0.8601	0.8889	**0.11**
Lags 0–4	0.8119	0.7987	0.8253	< 0.05	0.8400	0.8246	0.8557	**0.06**
2001								
Lag 0	1.0206	1.0132	1.0280	0.58	1.0262	1.0178	1.0346	0.54
Lags 0–1	1.0358	1.0272	1.0446	0.41	1.0482	1.0381	1.0584	0.34
Lags 0–2	1.0289	1.0192	1.0387	0.56	1.0418	1.0303	1.0534	0.47
Lags 0–3	1.0349	1.0241	1.0457	0.52	1.0398	1.0271	1.0527	0.53
Lags 0–4	1.0135	1.0021	1.0251	0.82	1.0164	1.0029	1.0301	0.81
2002								
Lag 0	1.0415	1.0343	1.0488	0.25	1.0284	1.0206	1.0362	0.47
Lags 0–1	1.0618	1.0532	1.0704	0.15	1.0469	1.0376	1.0564	0.32
Lags 0–2	1.0774	1.0678	1.0872	0.10	1.0653	1.0547	1.0760	0.21
Lags 0–3	1.0574	1.0469	1.0680	0.27	1.0493	1.0378	1.0608	0.39
Lags 0–4	1.0259	1.0147	1.0372	0.65	1.0325	1.0201	1.0451	0.60
2003								
Lag 0	1.0359	1.0283	1.0436	0.35	1.0578	1.0493	1.0663	0.17
Lags 0–1	1.0248	1.0158	1.0340	0.59	1.0630	1.0528	1.0733	0.22
Lags 0–2	1.0343	1.0237	1.0451	0.52	1.0781	1.0660	1.0904	0.19
Lags 0–3	1.0389	1.0269	1.0511	0.52	1.0832	1.0692	1.0974	0.23
Lags 0–4	1.0443	1.0311	1.0578	0.51	1.0648	1.0492	1.0806	0.40
2004								
Lag 0	1.0119	1.0070	1.0167	0.63	1.0219	1.0167	1.0271	0.40
Lags 0–1	1.0274	1.0217	1.0331	0.34	1.0255	1.0194	1.0316	0.41
Lags 0–2	1.0143	1.0077	1.0209	0.67	1.0153	1.0083	1.0223	0.67
Lags 0–3	0.9966	0.9894	1.0040	0.93	1.0068	0.9990	1.0146	0.87
Lags 0–4	1.0059	0.9978	1.0141	0.89	1.0179	1.0092	1.0267	0.69
2005								
Lag 0	1.0120	1.0075	1.0165	0.60	1.0236	1.0183	1.0288	0.37
Lags 0–1	1.0116	1.0065	1.0168	0.66	1.0334	1.0272	1.0395	0.28
Lags 0–2	1.0089	1.0031	1.0147	0.76	1.0364	1.0295	1.0434	0.30
Lags 0–3	1.0118	1.0054	1.0182	0.72	1.0402	1.0325	1.0479	0.30
Lags 0–4	1.0124	1.0055	1.0194	0.73	1.0431	1.0348	1.0514	0.30
2006								
Lag 0	0.9948	0.9902	0.9994	0.83	0.9735	0.9686	0.9784	0.29
Lags 0–1	0.9852	0.9799	0.9905	0.59	0.9605	0.9548	0.9662	0.18
Lags 0–2	0.9720	0.9660	0.9781	0.37	0.9564	0.9499	0.9629	0.20
Lags 0–3	0.9730	0.9663	0.9797	0.44	0.9596	0.9523	0.9670	0.29
Lags 0–4	0.9799	0.9725	0.9873	0.60	0.9634	0.9553	0.9715	0.39
2007								
Lag 0	0.9994	0.9967	1.0022	0.97	0.9989	0.9959	1.0018	0.94
Lags 0–1	0.9910	0.9878	0.9941	0.58	0.9949	0.9915	0.9983	0.77
Lags 0–2	0.9904	0.9868	0.9939	0.60	0.9946	0.9908	0.9984	0.78
Lags 0–3	0.9884	0.9844	0.9923	0.57	0.9937	0.9894	0.9979	0.77
Lags 0–4	0.9846	0.9803	0.9889	0.49	0.9950	0.9904	0.9997	0.83
2008								
Lag 0	1.0107	1.0080	1.0135	0.44	1.0330	1.0299	1.0362	**< 0.05**
Lags 0–1	1.0070	1.0038	1.0101	0.66	1.0296	1.0260	1.0331	0.10
Lags 0–2	1.0062	1.0027	1.0097	0.73	1.0272	1.0232	1.0312	0.18
Lags 0–3	1.0026	0.9988	1.0065	0.89	1.0226	1.0182	1.0270	0.31
Lags 0–4	1.0005	0.9964	1.0047	0.98	1.0192	1.0144	1.0240	0.43
2009								
Lag 0	1.0077	1.0052	1.0101	0.54	0.9918	0.9892	0.9945	0.55
Lags 0–1	1.0108	1.0080	1.0137	0.45	0.9882	0.9851	0.9913	0.45
Lags 0–2	1.0131	1.0099	1.0162	0.42	0.9873	0.9838	0.9907	0.47
Lags 0–3	1.0162	1.0128	1.0197	0.36	0.9861	0.9824	0.9899	0.48
Lags 0–4	1.0177	1.0140	1.0215	0.35	0.9797	0.9756	0.9838	0.33
2010								
Lag 0	0.9817	0.9791	0.9844	0.18	0.9947	0.9919	0.9975	0.71
Lags 0–1	0.9703	0.9674	0.9732	< 0.05	0.9812	0.9781	0.9843	**0.24**
Lags 0–2	0.9654	0.9623	0.9686	< 0.05	0.9783	0.9749	0.9817	**0.22**
Lags 0–3	0.9632	0.9598	0.9667	< 0.05	0.9778	0.9741	0.9815	**0.25**
Lags 0–4	0.9561	0.9524	0.9598	< 0.05	0.9716	0.9676	0.9757	**0.17**
2011								
Lag 0	1.0303	1.0274	1.0332	< 0.05	1.0283	1.0252	1.0315	**0.08**
Lags 0–1	1.0182	1.0149	1.0215	0.27	1.0200	1.0164	1.0236	0.27
Lags 0–2	1.0074	1.0038	1.0111	0.69	1.0132	1.0092	1.0172	0.51
Lags 0–3	1.0092	1.0052	1.0133	0.65	1.0158	1.0114	1.0202	0.48
Lags 0–4	1.0116	1.0072	1.0160	0.60	1.0186	1.0137	1.0234	0.45
2012								
Lag 0	1.0170	1.0142	1.0198	0.23	1.0058	1.0029	1.0088	0.70
Lags 0–1	1.0267	1.0236	1.0299	0.09	1.0137	1.0104	1.0170	0.41
Lags 0–2	1.0389	1.0354	1.0423	< 0.05	1.0293	1.0256	1.0329	**0.11**
Lags 0–3	1.0430	1.0393	1.0468	< 0.05	1.0338	1.0298	1.0378	**0.09**
Lags 0–4	1.0450	1.0409	1.0490	< 0.05	1.0359	1.0316	1.0402	**0.10**
2013								
Lag 0	0.9742	0.9713	0.9772	0.09	0.9900	0.9867	0.9934	0.57
Lags 0–1	0.9638	0.9604	0.9671	< 0.05	0.9816	0.9778	0.9854	0.35
Lags 0–2	0.9661	0.9624	0.9697	0.07	0.9935	0.9893	0.9978	**0.77**
Lags 0–3	0.9654	0.9615	0.9694	0.09	0.9987	0.9940	1.0034	0.96
Lags 0–4	0.9676	0.9633	0.9719	0.14	1.0040	0.9989	1.0091	0.88
2014								
Lag 0	1.0251	1.0217	1.0285	0.14	1.0152	1.0115	1.0188	0.41
Lags 0–1	1.0356	1.0318	1.0394	0.06	1.0171	1.0130	1.0214	0.42
Lags 0–2	1.0423	1.0381	1.0464	< 0.05	1.0251	1.0204	1.0298	0.29
Lags 0–3	1.0377	1.0332	1.0422	0.09	1.0203	1.0152	1.0255	0.44
Lags 0–4	1.0493	1.0445	1.0541	< 0.05	1.0338	1.0281	1.0395	**0.24**
2015								
Lag 0	1.0060	1.0034	1.0086	0.65	1.0277	1.0247	1.0307	0.07
Lags 0–1	1.0075	1.0046	1.0105	0.62	1.0329	1.0294	1.0363	0.06
Lags 0–2	1.0052	1.0019	1.0085	0.76	1.0323	1.0284	1.0361	0.10
Lags 0–3	1.0061	1.0024	1.0098	0.75	1.0305	1.0263	1.0348	0.15
Lags 0–4	1.0094	1.0053	1.0135	0.65	1.0332	1.0286	1.0379	0.15
2016								
Lag 0	1.0430	1.0388	1.0472	< 0.05	1.0173	1.0128	1.0219	**0.45**
Lags 0–1	1.0267	1.0220	1.0314	0.25	1.0002	0.9952	1.0053	0.99
Lags 0–2	1.0262	1.0211	1.0313	0.31	0.9940	0.9885	0.9996	0.83
Lags 0–3	1.0282	1.0227	1.0337	0.31	0.9899	0.9840	0.9959	0.74
Lags 0–4	1.0328	1.0269	1.0388	0.27	0.9904	0.9840	0.9968	0.77
