# Supplementary material for: Modification of the PM2.5- and extreme heat-mortality relationships by historical redlining: a case-crossover study in thirteen U.S. states
Source: Environ Health. 2024 Feb 7;23:16. doi: 10.1186/s12940-024-01055-5 (PMC10851491; doi:10.1186/s12940-024-01055-5)
Supplement: Supplementary file 1 — Additional file 1: Supplement A. Estimated odds ratios for the main effects of exposure to extreme heat on mortality and interactions with HOLC grade D, using different cutoffs for HOLC grade apportionment. Supplement B. Estimated odds ratios for the main effects of each 10 µg/m-3 increase in ambient PM2.5 on mortality and interactions with HOLC grade D, using different cutoffs for HOLC grade apportionment. Supplement C. Estimated odds ratios for the main effects of exposure to extreme heat on mortality and interactions with HOLC grade D, using different cutoffs for extreme heat. Supplement D. Estimated odds ratios for the main effects of exposure to extreme heat on all-cause mortality and interactions with HOLC grade D, within different subpopulations. Supplement E. Estimated odds ratios for the main effects of each 10 µg/m-3 increase in ambient PM2.5 on all-cause mortality and interactions with HOLC grade D, within different subpopulations. Supplement F. Estimated odds ratios for the main effects of exposure to extreme heat on mortality and interactions with HOLC grade D, by state. Supplement G. Estimated odds ratios for the main effects of exposure to PM2.5 on mortality and interactions with HOLC grade D, by state. Supplement H. Estimated odds ratios for the main effects of exposure to extreme heat on mortality and interactions with HOLC grade D, by year. Supplement I. Estimated odds ratios for the main effects of exposure to PM2.5 on mortality and interactions with HOLC grade D, by year. [file 12940_2024_1055_MOESM1_ESM.docx]

# Supplementary Material

Supplement A: Estimated odds ratios for the main effects of exposure to extreme heat on mortality and interactions with HOLC grade D, using different cutoffs for HOLC grade apportionment

|  | Main effect | | | | Interaction | | | |
| --- | --- | --- | --- | --- | --- | --- | --- | --- |
| Heat day | Estimate | 2.5% | 97.5% | p | Estimate | 2.5% | 97.5% | p |
| Population cutoff for apportionment = 50% | | | | | | | | |
| Any day | 1.013 | 1.0098 | 1.0161 | < 0.05 | 1.0211 | 1.0059 | 1.0364 | < 0.05 |
| 1^st^ day | 1.011 | 1.0062 | 1.0149 | < 0.05 | 1.0096 | 0.9867 | 1.0332 | 0.41 |
| 2^nd^ day | 1.008 | 1.0025 | 1.0130 | < 0.05 | 1.0206 | 0.9923 | 1.0496 | 0.16 |
| 3^rd^ day | 1.013 | 1.0064 | 1.0194 | < 0.05 | 1.0273 | 0.9926 | 1.0633 | 0.13 |
| Population cutoff for apportionment = 90% | | | | | | | | |
| Any day | 1.013 | 1.0100 | 1.0164 | < 0.05 | 1.0218 | 1.0031 | 1.0408 | < 0.05 |
| 1^st^ day | 1.011 | 1.0065 | 1.0151 | < 0.05 | 1.0056 | 0.9773 | 1.0346 | 0.70 |
| 2^nd^ day | 1.008 | 1.0028 | 1.0133 | < 0.05 | 1.0195 | 0.9846 | 1.0557 | 0.28 |
| 3^rd^ day | 1.013 | 1.0070 | 1.0199 | < 0.05 | 1.0155 | 0.9731 | 1.0597 | 0.48 |
| Population cutoff for apportionment = 99% | | | | | | | | |
| Any day | 1.013 | 1.0101 | 1.0164 | < 0.05 | 1.0222 | 1.0021 | 1.0428 | < 0.05 |
| 1^st^ day | 1.011 | 1.0064 | 1.0150 | < 0.05 | 1.0098 | 0.9793 | 1.0413 | 0.53 |
| 2^nd^ day | 1.008 | 1.0028 | 1.0133 | < 0.05 | 1.0208 | 0.9832 | 1.0599 | 0.28 |
| 3^rd^ day | 1.014 | 1.0071 | 1.0200 | < 0.05 | 1.0119 | 0.9664 | 1.0596 | 0.61 |

Supplement B: Estimated odds ratios for the main effects of each 10 µg/m^-3^ increase in ambient PM_2.5_ on mortality and interactions with HOLC grade D, using different cutoffs for HOLC grade apportionment

|  | Main effect | | | | Interaction | | | |
| --- | --- | --- | --- | --- | --- | --- | --- | --- |
| Exposure | Estimate | 2.5% | 97.5% | p | Estimate | 2.5% | 97.5% | p |
| Population cutoff for apportionment = 50% | | | | | | | | |
| Lag 0 | 1.0084 | 1.0082 | 1.0085 | < 0.05 | 1.0051 | 1.0044 | 1.0058 | 0.15 |
| Lags 0-1 | 1.0102 | 1.0100 | 1.0103 | < 0.05 | 1.0035 | 1.0027 | 1.0043 | 0.38 |
| Lags 0-2 | 1.0091 | 1.0089 | 1.0093 | < 0.05 | 1.0028 | 1.0020 | 1.0037 | 0.52 |
| Lags 0-3 | 1.0078 | 1.0076 | 1.0080 | < 0.05 | 1.0022 | 1.0012 | 1.0031 | 0.65 |
| Lags 0-4 | 1.0084 | 1.0082 | 1.0085 | < 0.05 | 1.0021 | 1.0011 | 1.0032 | 0.68 |
| Population cutoff for apportionment = 90% | | | | | | | | |
| Lag 0 | 1.0083 | 1.0082 | 1.0085 | < 0.05 | 1.0093 | 1.0084 | 1.0101 | < 0.05 |
| Lags 0-1 | 1.0101 | 1.0100 | 1.0103 | < 0.05 | 1.0069 | 1.0059 | 1.0078 | 0.15 |
| Lags 0-2 | 1.0090 | 1.0088 | 1.0092 | < 0.05 | 1.0061 | 1.0051 | 1.0072 | 0.26 |
| Lags 0-3 | 1.0077 | 1.0075 | 1.0079 | < 0.05 | 1.0057 | 1.0045 | 1.0069 | 0.34 |
| Lags 0-4 | 1.0070 | 1.0068 | 1.0072 | < 0.05 | 1.0064 | 1.0051 | 1.0076 | 0.32 |
| Population cutoff for apportionment = 99% | | | | | | | | |
| Lag 0 | 1.0084 | 1.0082 | 1.0085 | < 0.05 | 1.0088 | 1.0079 | 1.0098 | 0.05 |
| Lags 0-1 | 1.0101 | 1.0100 | 1.0103 | < 0.05 | 1.0075 | 1.0065 | 1.0085 | 0.15 |
| Lags 0-2 | 1.0090 | 1.0088 | 1.0092 | < 0.05 | 1.0068 | 1.0056 | 1.0079 | 0.24 |
| Lags 0-3 | 1.0077 | 1.0075 | 1.0079 | < 0.05 | 1.0068 | 1.0056 | 1.0081 | 0.28 |
| Lags 0-4 | 1.0070 | 1.0068 | 1.0072 | < 0.05 | 1.0069 | 1.0055 | 1.0082 | 0.32 |

Supplement C: Estimated odds ratios for the main effects of exposure to extreme heat on mortality and interactions with HOLC grade D, using different cutoffs for extreme heat

|  | Main effect | | | | Interaction | | | |
| --- | --- | --- | --- | --- | --- | --- | --- | --- |
| Heat day | Estimate | 2.5% | 97.5% | p | Estimate | 2.5% | 97.5% | p |
| Minimum temperature cutoff for extreme heat = 85^th^ percentile | | | | | | | | |
| Any day | 1.0109 | 1.0080 | 1.0138 | < 0.05 | 1.0279 | 1.0109 | 1.045 | < 0.05 |
| 1^st^ day | 1.0069 | 1.0030 | 1.0109 | < 0.05 | 1.0340 | 1.0074 | 1.061 | < 0.05 |
| 2^nd^ day | 1.0124 | 1.0077 | 1.0171 | < 0.05 | 0.9972 | 0.9665 | 1.029 | 0.86 |
| 3^rd^ day | 1.0079 | 1.0024 | 1.0133 | < 0.05 | 1.0142 | 0.9779 | 1.052 | 0.45 |
| Minimum temperature cutoff for extreme heat = 90^th^ percentile | | | | | | | | |
| Any day | 1.0132 | 1.0100 | 1.0164 | < 0.05 | 1.0218 | 1.0031 | 1.041 | < 0.05 |
| 1^st^ day | 1.0108 | 1.0065 | 1.0151 | < 0.05 | 1.0056 | 0.9773 | 1.035 | 0.70 |
| 2^nd^ day | 1.0080 | 1.0028 | 1.0133 | < 0.05 | 1.0195 | 0.9846 | 1.056 | 0.28 |
| 3^rd^ day | 1.0134 | 1.0070 | 1.0199 | < 0.05 | 1.0155 | 0.9731 | 1.060 | 0.48 |
| Minimum temperature cutoff for extreme heat = 99^th^ percentile | | | | | | | | |
| Any day | 1.0217 | 1.0143 | 1.0292 | < 0.05 | 1.0266 | 0.9793 | 1.076 | 0.28 |
| 1^st^ day | 1.0181 | 1.0092 | 1.0271 | < 0.05 | 1.0368 | 0.9793 | 1.098 | 0.21 |
| 2^nd^ day | 1.0227 | 1.0081 | 1.0376 | < 0.05 | 1.0021 | 0.9081 | 1.106 | 0.97 |
| 3^rd^ day | 1.0501 | 1.0238 | 1.0771 | < 0.05 | 1.0034 | 0.8419 | 1.196 | 0.97 |

Supplement D: Estimated odds ratios for the main effects of exposure to extreme heat on all-cause mortality and interactions with HOLC grade D, within different subpopulations

|  | Main effect | | | | Interaction | | | |
| --- | --- | --- | --- | --- | --- | --- | --- | --- |
| Heat day | Estimate | 2.5% | 97.5% | p | Estimate | 2.5% | 97.5% | p |
| Whole population | | | | | | | | |
| Any day | 1.0132 | 1.0100 | 1.0164 | < 0.05 | 1.0218 | 1.0031 | 1.0408 | < 0.05 |
| 1^st^ day | 1.0108 | 1.0065 | 1.0151 | < 0.05 | 1.0056 | 0.9773 | 1.0346 | 0.70 |
| 2^nd^ day | 1.0080 | 1.0028 | 1.0133 | < 0.05 | 1.0195 | 0.9846 | 1.0557 | 0.28 |
| 3^rd^ day | 1.0134 | 1.0070 | 1.0199 | < 0.05 | 1.0155 | 0.9731 | 1.0597 | 0.48 |
| Black individuals | | | | | | | | |
| Any day | 1.0058 | 0.9956 | 1.0160 | 0.27 | 1.0043 | 0.9739 | 1.0356 | 0.79 |
| 1^st^ day | 1.0008 | 0.9872 | 1.0146 | 0.91 | 0.9977 | 0.9516 | 1.0460 | 0.92 |
| 2^nd^ day | 0.9975 | 0.9810 | 1.0141 | 0.76 | 1.0561 | 0.9978 | 1.1178 | 0.06 |
| 3^rd^ day | 1.0191 | 0.9988 | 1.0399 | 0.07 | 1.0099 | 0.9417 | 1.0830 | 0.78 |
| >50% Black neighborhoods | | | | | | | | |
| Any day | 1.0067 | 0.9936 | 1.0201 | 0.32 | 1.0017 | 0.9685 | 1.0361 | 0.92 |
| 1^st^ day | 1.0086 | 0.9913 | 1.0261 | 0.33 | 0.9707 | 0.9216 | 1.0223 | 0.26 |
| 2^nd^ day | 1.0007 | 0.9801 | 1.0218 | 0.94 | 1.0399 | 0.9775 | 1.1062 | 0.22 |
| 3^rd^ day | 1.0133 | 0.9877 | 1.0395 | 0.31 | 1.0272 | 0.9520 | 1.1084 | 0.49 |
| White individuals | | | | | | | | |
| Any day | 1.0141 | 1.0107 | 1.0175 | < 0.05 | 1.0403 | 1.0146 | 1.0667 | < 0.05 |
| 1^st^ day | 1.0127 | 1.0080 | 1.0173 | < 0.05 | 1.0262 | 0.9874 | 1.0666 | 0.19 |
| 2^nd^ day | 1.0089 | 1.0033 | 1.0145 | < 0.05 | 0.9954 | 0.9487 | 1.0443 | 0.85 |
| 3^rd^ day | 1.0110 | 1.0041 | 1.0180 | < 0.05 | 1.0327 | 0.9745 | 1.0944 | 0.28 |
| >50% White neighborhoods | | | | | | | | |
| Any day | 1.0128 | 1.0093 | 1.0162 | < 0.05 | 1.0383 | 1.0079 | 1.0695 | < 0.05 |
| 1^st^ day | 1.0118 | 1.0071 | 1.0164 | < 0.05 | 1.0359 | 0.9903 | 1.0836 | 0.12 |
| 2^nd^ day | 1.0060 | 1.0003 | 1.0116 | < 0.05 | 1.0064 | 0.9516 | 1.0645 | 0.82 |
| 3^rd^ day | 1.0139 | 1.0069 | 1.0209 | < 0.05 | 1.0259 | 0.9585 | 1.0980 | 0.46 |

Supplement E: Estimated odds ratios for the main effects of each 10 µg/m_-3_ increase in ambient PM^2.5^ on all-cause mortality and interactions with HOLC grade D, within different subpopulations

|  | Main effect | | | | Interaction | | | |
| --- | --- | --- | --- | --- | --- | --- | --- | --- |
| Heat day | Estimate | 2.5% | 97.5% | p | Estimate | 2.5% | 97.5% | p |
| Whole population | | | | | | | | |
| Lag 0 | 1.0083 | 1.0082 | 1.0085 | < 0.05 | 1.0093 | 1.0084 | 1.0101 | < 0.05 |
| Lags 0-1 | 1.0101 | 1.0100 | 1.0103 | < 0.05 | 1.0069 | 1.0059 | 1.0078 | 0.15 |
| Lags 0-2 | 1.0090 | 1.0088 | 1.0092 | < 0.05 | 1.0061 | 1.0051 | 1.0072 | 0.26 |
| Lags 0-3 | 1.0077 | 1.0075 | 1.0079 | < 0.05 | 1.0057 | 1.0045 | 1.0069 | 0.34 |
| Lags 0-4 | 1.0070 | 1.0068 | 1.0072 | < 0.05 | 1.0064 | 1.0051 | 1.0076 | 0.32 |
| Black individuals | | | | | | | | |
| Lag 0 | 1.0117 | 1.0112 | 1.0121 | < 0.05 | 1.0020 | 1.0006 | 1.0034 | 0.78 |
| Lags 0-1 | 1.0130 | 1.0124 | 1.0135 | < 0.05 | 1.0004 | 0.9988 | 1.0020 | 0.96 |
| Lags 0-2 | 1.0130 | 1.0124 | 1.0136 | < 0.05 | 1.0024 | 1.0006 | 1.0042 | 0.79 |
| Lags 0-3 | 1.0128 | 1.0122 | 1.0134 | < 0.05 | 1.0028 | 1.0008 | 1.0047 | 0.78 |
| Lags 0-4 | 1.0127 | 1.0121 | 1.0134 | < 0.05 | 1.0041 | 1.0020 | 1.0063 | 0.70 |
| >50% Black neighborhoods | | | | | | | | |
| Lag 0 | 1.0065 | 1.0059 | 1.0071 | < 0.05 | 1.0099 | 1.0084 | 1.0114 | 0.20 |
| Lags 0-1 | 1.0060 | 1.0053 | 1.0066 | 0.08 | 1.0108 | 1.0091 | 1.0126 | 0.22 |
| Lags 0-2 | 1.0061 | 1.0053 | 1.0068 | 0.10 | 1.0084 | 1.0064 | 1.0103 | 0.40 |
| Lags 0-3 | 1.0039 | 1.0031 | 1.0047 | 0.34 | 1.0081 | 1.0060 | 1.0103 | 0.46 |
| Lags 0-4 | 1.0019 | 1.0011 | 1.0028 | 0.66 | 1.0093 | 1.0069 | 1.0116 | 0.44 |
| White individuals | | | | | | | | |
| Lag 0 | 1.0081 | 1.0079 | 1.0082 | < 0.05 | 1.0115 | 1.0103 | 1.0126 | < 0.05 |
| Lags 0-1 | 1.0100 | 1.0098 | 1.0101 | < 0.05 | 1.0106 | 1.0093 | 1.0119 | 0.11 |
| Lags 0-2 | 1.0086 | 1.0084 | 1.0088 | < 0.05 | 1.0080 | 1.0066 | 1.0094 | 0.27 |
| Lags 0-3 | 1.0071 | 1.0069 | 1.0073 | < 0.05 | 1.0065 | 1.0049 | 1.0081 | 0.42 |
| Lags 0-4 | 1.0063 | 1.0061 | 1.0066 | < 0.05 | 1.0065 | 1.0048 | 1.0082 | 0.46 |
| >50% White neighborhoods | | | | | | | | |
| Lag 0 | 1.0079 | 1.0077 | 1.0081 | < 0.05 | 1.0049 | 1.0035 | 1.0063 | 0.48 |
| Lags 0-1 | 1.0098 | 1.0096 | 1.0100 | < 0.05 | 1.0021 | 1.0006 | 1.0037 | 0.79 |
| Lags 0-2 | 1.0082 | 1.0080 | 1.0084 | < 0.05 | 1.0007 | 0.9990 | 1.0025 | 0.94 |
| Lags 0-3 | 1.0067 | 1.0065 | 1.0069 | < 0.05 | 0.9989 | 0.9970 | 1.0009 | 0.91 |
| Lags 0-4 | 1.0058 | 1.0056 | 1.0061 | < 0.05 | 0.9963 | 0.9942 | 0.9984 | 0.73 |

Supplement F: Estimated odds ratios for the main effects of exposure to extreme heat on mortality and interactions with HOLC grade D, by state

|  | Main effect | | | | Interaction | | | |
| --- | --- | --- | --- | --- | --- | --- | --- | --- |
| Heat day | Estimate | 2.5% | 97.5% | p | Estimate | 2.5% | 97.5% | p |
| California | | | | | | | | |
| Any day | 1.0150 | 1.0087 | 1.0214 | < 0.05 | 1.0239 | 0.9882 | 1.0607 | 0.19 |
| 1st day | 1.0166 | 1.0069 | 1.0264 | < 0.05 | 0.9851 | 0.9306 | 1.0429 | 0.61 |
| 2nd day | 1.0024 | 0.9906 | 1.0142 | 0.69 | 1.0389 | 0.9679 | 1.1151 | 0.29 |
| 3rd day | 1.0117 | 0.9973 | 1.0262 | 0.11 | 1.0402 | 0.9549 | 1.1331 | 0.37 |
| Florida | | | | | | | | |
| Any day | 1.0042 | 0.9956 | 1.0129 | 0.34 | 1.0089 | 0.9381 | 1.0849 | 0.81 |
| 1st day | 1.0066 | 0.9968 | 1.0164 | 0.19 | 0.8867 | 0.7949 | 0.9891 | < 0.05 |
| 2nd day | 0.9954 | 0.9833 | 1.0077 | 0.46 | 0.9972 | 0.8718 | 1.1407 | 0.97 |
| 3rd day | 1.0053 | 0.9898 | 1.0210 | 0.51 | 1.1187 | 0.9424 | 1.3279 | 0.20 |
| Georgia | | | | | | | | |
| Any day | 0.9935 | 0.9658 | 1.0219 | 0.65 | 1.0635 | 0.8713 | 1.2982 | 0.54 |
| 1st day | 0.9909 | 0.9589 | 1.0239 | 0.58 | 1.0903 | 0.7976 | 1.4905 | 0.59 |
| 2nd day | 1.0154 | 0.9767 | 1.0557 | 0.44 | 0.8493 | 0.5684 | 1.2689 | 0.43 |
| 3rd day | 0.9768 | 0.9333 | 1.0224 | 0.31 | 1.3865 | 0.8795 | 2.1856 | 0.16 |
| Illinois | | | | | | | | |
| Any day | 0.9945 | 0.9805 | 1.0087 | 0.45 | 1.0152 | 0.9739 | 1.0583 | 0.48 |
| 1st day | 1.0089 | 0.9944 | 1.0235 | 0.23 | 0.9766 | 0.9163 | 1.0409 | 0.47 |
| 2nd day | 0.9912 | 0.9740 | 1.0087 | 0.32 | 1.0812 | 1.0024 | 1.1663 | < 0.05 |
| 3rd day | 1.0148 | 0.9933 | 1.0368 | 0.18 | 0.9878 | 0.8984 | 1.0860 | 0.80 |
| Indiana | | | | | | | | |
| Any day | 0.9819 | 0.9385 | 1.0273 | 0.43 | 1.0256 | 0.8980 | 1.1713 | 0.71 |
| 1st day | 1.0208 | 0.9802 | 1.0631 | 0.32 | 0.9508 | 0.7765 | 1.1641 | 0.62 |
| 2nd day | 1.0022 | 0.9552 | 1.0515 | 0.93 | 1.0904 | 0.8633 | 1.3773 | 0.47 |
| 3rd day | 0.9960 | 0.9364 | 1.0594 | 0.90 | 1.0593 | 0.8005 | 1.4017 | 0.69 |
| Kansas | | | | | | | | |
| Any day | 1.0164 | 0.9618 | 1.0741 | 0.56 | 0.9867 | 0.7948 | 1.2248 | 0.90 |
| 1st day | 0.9920 | 0.9349 | 1.0525 | 0.79 | 1.0590 | 0.7564 | 1.4827 | 0.74 |
| 2nd day | 0.9833 | 0.9118 | 1.0604 | 0.66 | 0.7153 | 0.4172 | 1.2265 | 0.22 |
| 3rd day | 0.9584 | 0.8742 | 1.0508 | 0.37 | 0.6994 | 0.3742 | 1.3071 | 0.26 |
| Massachusetts | | | | | | | | |
| Any day | 1.0090 | 0.9933 | 1.0249 | 0.26 | 1.0336 | 0.9793 | 1.0909 | 0.23 |
| 1st day | 1.0293 | 1.0141 | 1.0447 | < 0.05 | 1.0332 | 0.9530 | 1.1201 | 0.43 |
| 2nd day | 1.0068 | 0.9888 | 1.0251 | 0.46 | 1.0112 | 0.9175 | 1.1144 | 0.82 |
| 3rd day | 0.9766 | 0.9544 | 0.9993 | < 0.05 | 1.0376 | 0.9195 | 1.1708 | 0.55 |
| Michigan | | | | | | | | |
| Any day | 0.9947 | 0.9771 | 1.0126 | 0.56 | 0.9166 | 0.8583 | 0.9788 | < 0.05 |
| 1st day | 1.0077 | 0.9905 | 1.0251 | 0.38 | 0.9491 | 0.8608 | 1.0464 | 0.29 |
| 2nd day | 1.0106 | 0.9898 | 1.0319 | 0.32 | 0.9356 | 0.8298 | 1.0550 | 0.28 |
| 3rd day | 1.0044 | 0.9786 | 1.0309 | 0.74 | 0.9029 | 0.7768 | 1.0493 | 0.18 |
| Missouri | | | | | | | | |
| Any day | 1.0208 | 0.9980 | 1.0441 | 0.07 | 1.0474 | 0.9477 | 1.1576 | 0.36 |
| 1st day | 1.0145 | 0.9912 | 1.0384 | 0.22 | 1.0663 | 0.9074 | 1.2532 | 0.44 |
| 2nd day | 1.0190 | 0.9908 | 1.0480 | 0.19 | 1.0331 | 0.8472 | 1.2597 | 0.75 |
| 3rd day | 1.0287 | 0.9959 | 1.0625 | 0.09 | 1.0194 | 0.8148 | 1.2753 | 0.87 |
| New Hampshire | | | | | | | | |
| Any day | 1.0007 | 0.9561 | 1.0474 | 0.98 | 0.7292 | 0.4301 | 1.2364 | 0.24 |
| 1st day | 0.9961 | 0.9545 | 1.0395 | 0.86 | 1.1650 | 0.5956 | 2.2786 | 0.66 |
| 2nd day | 1.0596 | 1.0053 | 1.1167 | < 0.05 | 0.2818 | 0.0671 | 1.1839 | 0.08 |
| 3rd day | 0.9796 | 0.9140 | 1.0499 | 0.56 | 0.7075 | 0.1639 | 3.0548 | 0.64 |
| New Jersey | | | | | | | | |
| Any day | 0.9732 | 0.9510 | 0.9959 | < 0.05 | 1.0203 | 0.9514 | 1.0942 | 0.57 |
| 1st day | 0.9871 | 0.9649 | 1.0099 | 0.27 | 1.1206 | 1.0069 | 1.2471 | < 0.05 |
| 2nd day | 1.0011 | 0.9740 | 1.0289 | 0.94 | 0.9623 | 0.8421 | 1.0996 | 0.57 |
| 3rd day | 1.0080 | 0.9756 | 1.0415 | 0.63 | 0.9680 | 0.8248 | 1.1361 | 0.69 |
| Ohio | | | | | | | | |
| Any day | 1.0083 | 0.9917 | 1.0251 | 0.33 | 1.0632 | 0.9809 | 1.1524 | 0.14 |
| 1st day | 1.0096 | 0.9938 | 1.0257 | 0.23 | 1.0894 | 0.9608 | 1.2353 | 0.18 |
| 2nd day | 1.0104 | 0.9919 | 1.0293 | 0.27 | 1.1124 | 0.9623 | 1.2860 | 0.15 |
| 3rd day | 1.0015 | 0.9789 | 1.0246 | 0.90 | 0.8973 | 0.7475 | 1.0771 | 0.24 |
| Texas | | | | | | | | |
| Any day | 1.0015 | 0.9933 | 1.0097 | 0.73 | 1.0671 | 0.9958 | 1.1435 | 0.07 |
| 1st day | 0.9931 | 0.9827 | 1.0035 | 0.19 | 1.0600 | 0.9544 | 1.1772 | 0.28 |
| 2nd day | 1.0053 | 0.9925 | 1.0183 | 0.42 | 0.9206 | 0.8023 | 1.0563 | 0.24 |
| 3rd day | 1.0240 | 1.0086 | 1.0398 | < 0.05 | 1.1452 | 0.9800 | 1.3382 | 0.09 |

Supplement G: Estimated odds ratios for the main effects of exposure to PM_2.5_ on mortality and interactions with HOLC grade D, by state

|  | Main effect | | | | Interaction | | | |
| --- | --- | --- | --- | --- | --- | --- | --- | --- |
| Exposure | Estimate | 2.5% | 97.5% | p | Estimate | 2.5% | 97.5% | p |
| California | | | | | | | | |
| Lag 0 | 1.0116 | 1.0113 | 1.0119 | < 0.05 | 1.0131 | 1.0115 | 1.0147 | 0.12 |
| Lags 0-1 | 1.0131 | 1.0128 | 1.0135 | < 0.05 | 1.0063 | 1.0046 | 1.0081 | 0.49 |
| Lags 0-2 | 1.0136 | 1.0132 | 1.0139 | < 0.05 | 1.0064 | 1.0045 | 1.0084 | 0.52 |
| Lags 0-3 | 1.0146 | 1.0142 | 1.0149 | < 0.05 | 1.0076 | 1.0055 | 1.0097 | 0.48 |
| Lags 0-4 | 1.0163 | 1.0160 | 1.0167 | < 0.05 | 1.0114 | 1.0092 | 1.0137 | 0.32 |
| Florida | | | | | | | | |
| Lag 0 | 0.9980 | 0.9975 | 0.9984 | 0.41 | 1.0115 | 1.0065 | 1.0165 | 0.65 |
| Lags 0-1 | 0.9967 | 0.9962 | 0.9972 | 0.23 | 1.0002 | 0.9946 | 1.0058 | 0.99 |
| Lags 0-2 | 0.9963 | 0.9957 | 0.9969 | 0.23 | 0.9885 | 0.9824 | 0.9947 | 0.72 |
| Lags 0-3 | 0.9943 | 0.9937 | 0.9950 | 0.09 | 0.9952 | 0.9886 | 1.0019 | 0.89 |
| Lags 0-4 | 0.9920 | 0.9913 | 0.9927 | < 0.05 | 1.0230 | 1.0159 | 1.0302 | 0.52 |
| Georgia | | | | | | | | |
| Lag 0 | 0.9896 | 0.9885 | 0.9907 | 0.07 | 1.0284 | 1.0187 | 1.0383 | 0.56 |
| Lags 0-1 | 0.9831 | 0.9819 | 0.9844 | < 0.05 | 1.0243 | 1.0134 | 1.0353 | 0.66 |
| Lags 0-2 | 0.9856 | 0.9842 | 0.9870 | < 0.05 | 1.0373 | 1.0251 | 1.0496 | 0.54 |
| Lags 0-3 | 0.9829 | 0.9814 | 0.9844 | < 0.05 | 1.0495 | 1.0361 | 1.0631 | 0.46 |
| Lags 0-4 | 0.9818 | 0.9802 | 0.9834 | < 0.05 | 1.0644 | 1.0499 | 1.0792 | 0.37 |
| Illinois | | | | | | | | |
| Lag 0 | 1.0043 | 1.0039 | 1.0048 | 0.07 | 1.0071 | 1.0054 | 1.0089 | 0.42 |
| Lags 0-1 | 1.0051 | 1.0045 | 1.0056 | 0.06 | 1.0075 | 1.0055 | 1.0095 | 0.46 |
| Lags 0-2 | 1.0025 | 1.0019 | 1.0031 | 0.4 | 1.0081 | 1.0058 | 1.0104 | 0.48 |
| Lags 0-3 | 0.9992 | 0.9986 | 0.9999 | 0.82 | 1.0058 | 1.0033 | 1.0083 | 0.65 |
| Lags 0-4 | 0.9951 | 0.9944 | 0.9958 | 0.16 | 1.0031 | 1.0004 | 1.0059 | 0.82 |
| Indiana | | | | | | | | |
| Lag 0 | 0.9706 | 0.9695 | 0.9718 | < 0.05 | 1.0775 | 1.0720 | 1.0830 | < 0.05 |
| Lags 0-1 | 0.9639 | 0.9626 | 0.9653 | < 0.05 | 1.0781 | 1.0719 | 1.0843 | < 0.05 |
| Lags 0-2 | 0.9607 | 0.9592 | 0.9621 | < 0.05 | 1.0657 | 1.0589 | 1.0725 | 0.05 |
| Lags 0-3 | 0.9583 | 0.9567 | 0.9599 | < 0.05 | 1.0581 | 1.0507 | 1.0655 | 0.11 |
| Lags 0-4 | 0.9601 | 0.9583 | 0.9619 | < 0.05 | 1.0586 | 1.0506 | 1.0667 | 0.14 |
| Kansas | | | | | | | | |
| Lag 0 | 1.0279 | 1.0258 | 1.0300 | < 0.05 | 0.9561 | 0.9444 | 0.9680 | 0.48 |
| Lags 0-1 | 1.0121 | 1.0097 | 1.0144 | 0.31 | 0.9770 | 0.9634 | 0.9908 | 0.74 |
| Lags 0-2 | 0.9969 | 0.9944 | 0.9995 | 0.81 | 1.0025 | 0.9873 | 1.0180 | 0.97 |
| Lags 0-3 | 0.9829 | 0.9801 | 0.9856 | 0.22 | 1.0396 | 1.0225 | 1.0570 | 0.65 |
| Lags 0-4 | 0.9739 | 0.9710 | 0.9769 | 0.09 | 1.0649 | 1.0460 | 1.0840 | 0.49 |
| Massachusetts | | | | | | | | |
| Lag 0 | 1.0099 | 1.0093 | 1.0104 | < 0.05 | 0.9984 | 0.9960 | 1.0009 | 0.90 |
| Lags 0-1 | 1.0166 | 1.0160 | 1.0173 | < 0.05 | 1.0046 | 1.0017 | 1.0075 | 0.76 |
| Lags 0-2 | 1.0149 | 1.0142 | 1.0157 | < 0.05 | 0.9955 | 0.9922 | 0.9988 | 0.79 |
| Lags 0-3 | 1.0134 | 1.0126 | 1.0142 | < 0.05 | 0.9887 | 0.9851 | 0.9923 | 0.54 |
| Lags 0-4 | 1.0106 | 1.0097 | 1.0114 | < 0.05 | 0.9839 | 0.9800 | 0.9879 | 0.43 |
| Michigan | | | | | | | | |
| Lag 0 | 1.0049 | 1.0044 | 1.0055 | 0.09 | 1.0121 | 1.0093 | 1.0149 | 0.39 |
| Lags 0-1 | 1.0093 | 1.0087 | 1.0100 | < 0.05 | 1.0026 | 0.9995 | 1.0057 | 0.87 |
| Lags 0-2 | 1.0072 | 1.0064 | 1.0079 | 0.06 | 1.0060 | 1.0024 | 1.0095 | 0.74 |
| Lags 0-3 | 1.0040 | 1.0032 | 1.0048 | 0.33 | 1.0059 | 1.0020 | 1.0098 | 0.77 |
| Lags 0-4 | 1.0024 | 1.0015 | 1.0033 | 0.59 | 1.0052 | 1.0010 | 1.0095 | 0.81 |
| Missouri | | | | | | | | |
| Lag 0 | 1.0174 | 1.0165 | 1.0183 | < 0.05 | 1.0262 | 1.0206 | 1.0319 | 0.35 |
| Lags 0-1 | 1.0190 | 1.0180 | 1.0200 | < 0.05 | 1.0195 | 1.0132 | 1.0258 | 0.54 |
| Lags 0-2 | 1.0177 | 1.0166 | 1.0188 | < 0.05 | 1.0311 | 1.0241 | 1.0381 | 0.38 |
| Lags 0-3 | 1.0145 | 1.0133 | 1.0157 | < 0.05 | 1.0159 | 1.0084 | 1.0235 | 0.68 |
| Lags 0-4 | 1.0078 | 1.0065 | 1.0091 | 0.23 | 0.9988 | 0.9909 | 1.0069 | 0.98 |
| New Hampshire | | | | | | | | |
| Lag 0 | 1.0243 | 1.0222 | 1.0265 | < 0.05 | 1.0593 | 1.0313 | 1.0881 | 0.67 |
| Lags 0-1 | 1.0183 | 1.0158 | 1.0209 | 0.15 | 0.9618 | 0.9318 | 0.9928 | 0.81 |
| Lags 0-2 | 1.0098 | 1.0070 | 1.0127 | 0.50 | 0.9259 | 0.8930 | 0.9601 | 0.68 |
| Lags 0-3 | 0.9932 | 0.9902 | 0.9963 | 0.67 | 0.8920 | 0.8570 | 0.9284 | 0.58 |
| Lags 0-4 | 0.9899 | 0.9866 | 0.9932 | 0.55 | 0.7826 | 0.7483 | 0.8185 | 0.28 |
| New Jersey | | | | | | | | |
| Lag 0 | 1.0152 | 1.0146 | 1.0159 | < 0.05 | 0.9974 | 0.9950 | 0.9999 | 0.84 |
| Lags 0-1 | 1.0152 | 1.0144 | 1.0159 | < 0.05 | 0.9943 | 0.9915 | 0.9972 | 0.69 |
| Lags 0-2 | 1.0121 | 1.0112 | 1.0129 | < 0.05 | 0.9920 | 0.9888 | 0.9952 | 0.63 |
| Lags 0-3 | 1.0095 | 1.0086 | 1.0105 | < 0.05 | 0.9906 | 0.9870 | 0.9943 | 0.61 |
| Lags 0-4 | 1.0064 | 1.0054 | 1.0074 | 0.22 | 0.9878 | 0.9839 | 0.9918 | 0.55 |
| Ohio | | | | | | | | |
| Lag 0 | 1.0088 | 1.0083 | 1.0094 | < 0.05 | 0.9978 | 0.9942 | 1.0013 | 0.90 |
| Lags 0-1 | 1.0096 | 1.0090 | 1.0102 | < 0.05 | 0.9976 | 0.9937 | 1.0017 | 0.91 |
| Lags 0-2 | 1.0058 | 1.0051 | 1.0065 | 0.09 | 0.9937 | 0.9893 | 0.9982 | 0.78 |
| Lags 0-3 | 1.0030 | 1.0022 | 1.0037 | 0.43 | 0.9977 | 0.9928 | 1.0027 | 0.93 |
| Lags 0-4 | 1.0008 | 1.0000 | 1.0016 | 0.85 | 1.0000 | 0.9946 | 1.0054 | 1.00 |
| Texas | | | | | | | | |
| Lag 0 | 1.0087 | 1.0082 | 1.0092 | < 0.05 | 0.9901 | 0.9853 | 0.9949 | 0.69 |
| Lags 0-1 | 1.0096 | 1.0091 | 1.0102 | < 0.05 | 0.9836 | 0.9782 | 0.9891 | 0.56 |
| Lags 0-2 | 1.0049 | 1.0043 | 1.0055 | 0.09 | 0.9861 | 0.9800 | 0.9921 | 0.65 |
| Lags 0-3 | 1.0012 | 1.0006 | 1.0019 | 0.69 | 0.9957 | 0.9891 | 1.0023 | 0.90 |
| Lags 0-4 | 0.9999 | 0.9993 | 1.0006 | 0.98 | 1.0081 | 1.0010 | 1.0154 | 0.82 |

Supplement H: Estimated odds ratios for the main effects of exposure to extreme heat on mortality and interactions with HOLC grade D, by year

|  | Main effect | | | | Interaction | | | |
| --- | --- | --- | --- | --- | --- | --- | --- | --- |
| Heat day | Estimate | 2.5% | 97.5% | p | Estimate | 2.5% | 97.5% | p |
| 2000 | | | | | | | | |
| Any day | 0.9485 | 0.8922 | 1.0084 | 0.09 | 0.9443 | 0.7704 | 1.1574 | 0.58 |
| 1st day | 1.0005 | 0.9456 | 1.0587 | 0.99 | 1.1722 | 0.8804 | 1.5607 | 0.28 |
| 2nd day | 0.9966 | 0.9280 | 1.0702 | 0.92 | 1.0136 | 0.7107 | 1.4456 | 0.94 |
| 3rd day | 0.9807 | 0.8987 | 1.0703 | 0.66 | 0.6749 | 0.4151 | 1.0972 | 0.11 |
| 2001 | | | | | | | | |
| Any day | 0.9800 | 0.9237 | 1.0397 | 0.50 | 0.9519 | 0.7836 | 1.1562 | 0.62 |
| 1st day | 1.0296 | 0.9751 | 1.0872 | 0.29 | 1.0473 | 0.7939 | 1.3817 | 0.74 |
| 2nd day | 1.0372 | 0.9683 | 1.1110 | 0.30 | 0.7789 | 0.5235 | 1.1589 | 0.22 |
| 3rd day | 0.9641 | 0.8776 | 1.0591 | 0.45 | 0.7273 | 0.4233 | 1.2495 | 0.25 |
| 2002 | | | | | | | | |
| Any day | 0.9943 | 0.9341 | 1.0585 | 0.86 | 1.0534 | 0.8531 | 1.3009 | 0.63 |
| 1st day | 0.9934 | 0.9368 | 1.0534 | 0.82 | 0.8775 | 0.6143 | 1.2534 | 0.47 |
| 2nd day | 0.9974 | 0.9289 | 1.0709 | 0.94 | 0.6871 | 0.4391 | 1.0749 | 0.10 |
| 3rd day | 0.9641 | 0.8776 | 1.0591 | 0.45 | 0.7273 | 0.4233 | 1.2495 | 0.25 |
| 2003 | | | | | | | | |
| Any day | 0.9973 | 0.9315 | 1.0677 | 0.94 | 1.1611 | 0.9524 | 1.4156 | 0.14 |
| 1st day | 1.0660 | 1.0017 | 1.1344 | < 0.05 | 1.1348 | 0.8162 | 1.5778 | 0.45 |
| 2nd day | 0.9674 | 0.8944 | 1.0463 | 0.41 | 0.9508 | 0.6196 | 1.4588 | 0.82 |
| 3rd day | 0.9424 | 0.8576 | 1.0355 | 0.22 | 1.8368 | 1.2226 | 2.7594 | < 0.05 |
| 2004 | | | | | | | | |
| Any day | 1.0521 | 1.0106 | 1.0953 | < 0.05 | 1.0272 | 0.9058 | 1.1649 | 0.68 |
| 1st day | 1.0301 | 0.9911 | 1.0706 | 0.13 | 1.1850 | 0.9872 | 1.4224 | 0.07 |
| 2nd day | 1.0119 | 0.9655 | 1.0606 | 0.62 | 0.9205 | 0.7311 | 1.1589 | 0.48 |
| 3rd day | 1.0559 | 0.9986 | 1.1164 | 0.06 | 0.8037 | 0.5995 | 1.0774 | 0.14 |
| 2005 | | | | | | | | |
| Any day | 0.9626 | 0.9226 | 1.0043 | 0.08 | 1.1008 | 0.9672 | 1.2528 | 0.15 |
| 1st day | 0.9893 | 0.9503 | 1.0299 | 0.60 | 1.0888 | 0.8956 | 1.3237 | 0.39 |
| 2nd day | 0.9827 | 0.9368 | 1.0309 | 0.47 | 1.0895 | 0.8674 | 1.3686 | 0.46 |
| 3rd day | 0.9788 | 0.9238 | 1.0370 | 0.47 | 1.2123 | 0.9225 | 1.5930 | 0.17 |
| 2006 | | | | | | | | |
| Any day | 1.0057 | 0.9631 | 1.0502 | 0.80 | 1.0740 | 0.9331 | 1.2362 | 0.32 |
| 1st day | 1.0389 | 0.9929 | 1.0871 | 0.10 | 1.0997 | 0.8785 | 1.3766 | 0.41 |
| 2nd day | 1.0106 | 0.9600 | 1.0639 | 0.69 | 0.9565 | 0.7413 | 1.2342 | 0.73 |
| 3rd day | 0.9787 | 0.9237 | 1.0370 | 0.47 | 1.0563 | 0.7933 | 1.4066 | 0.71 |
| 2007 | | | | | | | | |
| Any day | 1.0045 | 0.9928 | 1.0164 | 0.45 | 0.9811 | 0.9142 | 1.0528 | 0.60 |
| 1st day | 1.0103 | 0.9953 | 1.0256 | 0.18 | 1.0253 | 0.9251 | 1.1362 | 0.63 |
| 2nd day | 0.9876 | 0.9695 | 1.0060 | 0.18 | 0.9631 | 0.8473 | 1.0947 | 0.56 |
| 3rd day | 1.0092 | 0.9868 | 1.0322 | 0.42 | 0.9373 | 0.7976 | 1.1014 | 0.43 |
| 2008 | | | | | | | | |
| Any day | 1.0028 | 0.9919 | 1.0139 | 0.61 | 0.9911 | 0.9321 | 1.0539 | 0.78 |
| 1st day | 0.9990 | 0.9845 | 1.0136 | 0.89 | 1.0069 | 0.9164 | 1.1063 | 0.89 |
| 2nd day | 1.0150 | 0.9978 | 1.0326 | 0.09 | 0.9947 | 0.8893 | 1.1125 | 0.93 |
| 3rd day | 1.0110 | 0.9904 | 1.0321 | 0.30 | 0.9543 | 0.8341 | 1.0917 | 0.50 |
| 2009 | | | | | | | | |
| Any day | 1.0017 | 0.9924 | 1.0110 | 0.73 | 0.9772 | 0.9251 | 1.0323 | 0.41 |
| 1st day | 1.0093 | 0.9964 | 1.0223 | 0.16 | 0.9188 | 0.8427 | 1.0018 | 0.05 |
| 2nd day | 0.9969 | 0.9814 | 1.0126 | 0.70 | 1.0168 | 0.9162 | 1.1285 | 0.75 |
| 3rd day | 0.9867 | 0.9670 | 1.0069 | 0.19 | 1.1579 | 1.0202 | 1.3143 | < 0.05 |
| 2010 | | | | | | | | |
| Any day | 1.0260 | 1.0159 | 1.0361 | < 0.05 | 1.0593 | 0.9985 | 1.1236 | 0.06 |
| 1st day | 1.0075 | 0.9939 | 1.0212 | 0.28 | 1.0025 | 0.9122 | 1.1018 | 0.96 |
| 2nd day | 1.0171 | 1.0005 | 1.0340 | < 0.05 | 1.1280 | 1.0108 | 1.2589 | < 0.05 |
| 3rd day | 1.0312 | 1.0109 | 1.0519 | < 0.05 | 1.0317 | 0.9023 | 1.1797 | 0.65 |
| 2011 | | | | | | | | |
| Any day | 1.0004 | 0.9906 | 1.0103 | 0.94 | 1.0357 | 0.9765 | 1.0986 | 0.24 |
| 1st day | 1.0148 | 1.0011 | 1.0288 | < 0.05 | 0.9989 | 0.9103 | 1.0960 | 0.98 |
| 2nd day | 0.9869 | 0.9705 | 1.0036 | 0.12 | 1.0005 | 0.8900 | 1.1247 | 0.99 |
| 3rd day | 1.0042 | 0.9838 | 1.0250 | 0.69 | 0.9915 | 0.8611 | 1.1417 | 0.91 |
| 2012 | | | | | | | | |
| Any day | 1.0305 | 1.0205 | 1.0406 | < 0.05 | 1.0083 | 0.9502 | 1.0700 | 0.78 |
| 1st day | 1.0189 | 1.0055 | 1.0325 | < 0.05 | 0.9758 | 0.8909 | 1.0688 | 0.60 |
| 2nd day | 1.0180 | 1.0016 | 1.0347 | < 0.05 | 0.9859 | 0.8774 | 1.1078 | 0.81 |
| 3rd day | 1.0237 | 1.0034 | 1.0444 | < 0.05 | 0.9844 | 0.8542 | 1.1345 | 0.83 |
| 2013 | | | | | | | | |
| Any day | 1.0168 | 1.0071 | 1.0266 | < 0.05 | 0.9884 | 0.9332 | 1.0469 | 0.69 |
| 1st day | 1.0043 | 0.9908 | 1.0181 | 0.53 | 0.9970 | 0.9071 | 1.0958 | 0.95 |
| 2nd day | 1.0108 | 0.9939 | 1.0279 | 0.21 | 0.9923 | 0.8816 | 1.1168 | 0.90 |
| 3rd day | 1.0225 | 1.0023 | 1.0431 | < 0.05 | 0.9897 | 0.8625 | 1.1356 | 0.88 |
| 2014 | | | | | | | | |
| Any day | 0.9986 | 0.9882 | 1.0091 | 0.79 | 0.9942 | 0.9311 | 1.0616 | 0.86 |
| 1st day | 0.9970 | 0.9829 | 1.0114 | 0.68 | 1.0027 | 0.9090 | 1.1060 | 0.96 |
| 2nd day | 0.9953 | 0.9782 | 1.0127 | 0.60 | 0.9296 | 0.8214 | 1.0520 | 0.25 |
| 3rd day | 1.0077 | 0.9858 | 1.0301 | 0.49 | 1.0659 | 0.9128 | 1.2448 | 0.42 |
| 2015 | | | | | | | | |
| Any day | 1.0051 | 0.9947 | 1.0156 | 0.33 | 1.1146 | 1.0457 | 1.1881 | < 0.05 |
| 1st day | 1.0071 | 0.9930 | 1.0214 | 0.33 | 1.0730 | 0.9751 | 1.1808 | 0.15 |
| 2nd day | 1.0177 | 1.0003 | 1.0354 | < 0.05 | 1.1101 | 0.9852 | 1.2509 | 0.09 |
| 3rd day | 1.0173 | 0.9963 | 1.0388 | 0.11 | 1.0207 | 0.8828 | 1.1802 | 0.78 |
| 2016 | | | | | | | | |
| Any day | 1.0296 | 1.0186 | 1.0406 | < 0.05 | 1.0456 | 0.9784 | 1.1175 | 0.19 |
| 1st day | 1.0277 | 1.0127 | 1.0430 | < 0.05 | 0.9830 | 0.8883 | 1.0877 | 0.74 |
| 2nd day | 1.0295 | 1.0112 | 1.0482 | < 0.05 | 1.1660 | 1.0383 | 1.3095 | < 0.05 |
| 3rd day | 1.0230 | 1.0007 | 1.0457 | < 0.05 | 0.9712 | 0.8292 | 1.1374 | 0.72 |

Supplement I: Estimated odds ratios for the main effects of exposure to PM_2.5_ on mortality and interactions with HOLC grade D, by year

|  | Main effect | | | | Interaction | | | |
| --- | --- | --- | --- | --- | --- | --- | --- | --- |
| Exposure | Estimate | 2.5% | 97.5% | p | Estimate | 2.5% | 97.5% | p |
| 2000 | | | | | | | | |
| Lag 0 | 0.9216 | 0.9197 | 0.9235 | < 0.05 | 1.0093 | 1.0003 | 1.0185 | 0.84 |
| Lags 0-1 | 0.8989 | 0.8967 | 0.9012 | < 0.05 | 0.9539 | 0.9435 | 0.9644 | 0.40 |
| Lags 0-2 | 0.8736 | 0.8711 | 0.8761 | < 0.05 | 0.8877 | 0.8763 | 0.8992 | 0.07 |
| Lags 0-3 | 0.8453 | 0.8426 | 0.8481 | < 0.05 | 0.8555 | 0.8431 | 0.8682 | < 0.05 |
| Lags 0-4 | 0.8278 | 0.8249 | 0.8308 | < 0.05 | 0.8119 | 0.7987 | 0.8253 | < 0.05 |
| 2001 | | | | | | | | |
| Lag 0 | 1.0651 | 1.0632 | 1.0670 | < 0.05 | 1.0206 | 1.0132 | 1.0280 | 0.58 |
| Lags 0-1 | 1.1001 | 1.0978 | 1.1025 | < 0.05 | 1.0358 | 1.0272 | 1.0446 | 0.41 |
| Lags 0-2 | 1.1190 | 1.1164 | 1.1217 | < 0.05 | 1.0289 | 1.0192 | 1.0387 | 0.56 |
| Lags 0-3 | 1.1407 | 1.1376 | 1.1437 | < 0.05 | 1.0349 | 1.0241 | 1.0457 | 0.52 |
| Lags 0-4 | 1.1591 | 1.1557 | 1.1625 | < 0.05 | 1.0135 | 1.0021 | 1.0251 | 0.82 |
| 2002 | | | | | | | | |
| Lag 0 | 0.9930 | 0.9915 | 0.9945 | 0.37 | 1.0415 | 1.0343 | 1.0488 | 0.25 |
| Lags 0-1 | 0.9988 | 0.9970 | 1.0006 | 0.90 | 1.0618 | 1.0532 | 1.0704 | 0.15 |
| Lags 0-2 | 0.9998 | 0.9977 | 1.0019 | 0.99 | 1.0774 | 1.0678 | 1.0872 | 0.10 |
| Lags 0-3 | 0.9972 | 0.9948 | 0.9995 | 0.81 | 1.0574 | 1.0469 | 1.0680 | 0.27 |
| Lags 0-4 | 0.9846 | 0.9820 | 0.9871 | 0.24 | 1.0259 | 1.0147 | 1.0372 | 0.65 |
| 2003 | | | | | | | | |
| Lag 0 | 0.9895 | 0.9877 | 0.9912 | 0.23 | 1.0359 | 1.0283 | 1.0436 | 0.35 |
| Lags 0-1 | 0.9850 | 0.9828 | 0.9871 | 0.17 | 1.0248 | 1.0158 | 1.0340 | 0.59 |
| Lags 0-2 | 0.9590 | 0.9566 | 0.9614 | < 0.05 | 1.0343 | 1.0237 | 1.0451 | 0.52 |
| Lags 0-3 | 0.9577 | 0.9550 | 0.9604 | < 0.05 | 1.0389 | 1.0269 | 1.0511 | 0.52 |
| Lags 0-4 | 0.9570 | 0.9540 | 0.9600 | < 0.05 | 1.0443 | 1.0311 | 1.0578 | 0.51 |
| 2004 | | | | | | | | |
| Lag 0 | 1.0398 | 1.0387 | 1.0410 | < 0.05 | 1.0119 | 1.0070 | 1.0167 | 0.63 |
| Lags 0-1 | 1.0428 | 1.0414 | 1.0441 | < 0.05 | 1.0274 | 1.0217 | 1.0331 | 0.34 |
| Lags 0-2 | 1.0410 | 1.0395 | 1.0426 | < 0.05 | 1.0143 | 1.0077 | 1.0209 | 0.67 |
| Lags 0-3 | 1.0428 | 1.0411 | 1.0445 | < 0.05 | 0.9966 | 0.9894 | 1.0040 | 0.93 |
| Lags 0-4 | 1.0388 | 1.0369 | 1.0407 | < 0.05 | 1.0059 | 0.9978 | 1.0141 | 0.89 |
| 2005 | | | | | | | | |
| Lag 0 | 1.0515 | 1.0503 | 1.0527 | < 0.05 | 1.0120 | 1.0075 | 1.0165 | 0.60 |
| Lags 0-1 | 1.0779 | 1.0764 | 1.0793 | < 0.05 | 1.0116 | 1.0065 | 1.0168 | 0.66 |
| Lags 0-2 | 1.1114 | 1.1097 | 1.1132 | < 0.05 | 1.0089 | 1.0031 | 1.0147 | 0.76 |
| Lags 0-3 | 1.1355 | 1.1335 | 1.1375 | < 0.05 | 1.0118 | 1.0054 | 1.0182 | 0.72 |
| Lags 0-4 | 1.1494 | 1.1472 | 1.1516 | < 0.05 | 1.0124 | 1.0055 | 1.0194 | 0.73 |
| 2006 | | | | | | | | |
| Lag 0 | 0.9910 | 0.9898 | 0.9921 | 0.14 | 0.9948 | 0.9902 | 0.9994 | 0.83 |
| Lags 0-1 | 0.9900 | 0.9886 | 0.9914 | 0.16 | 0.9852 | 0.9799 | 0.9905 | 0.59 |
| Lags 0-2 | 0.9841 | 0.9825 | 0.9856 | < 0.05 | 0.9720 | 0.9660 | 0.9781 | 0.37 |
| Lags 0-3 | 0.9782 | 0.9764 | 0.9799 | < 0.05 | 0.9730 | 0.9663 | 0.9797 | 0.44 |
| Lags 0-4 | 0.9759 | 0.9740 | 0.9778 | < 0.05 | 0.9799 | 0.9725 | 0.9873 | 0.60 |
| 2007 | | | | | | | | |
| Lag 0 | 1.0041 | 1.0036 | 1.0045 | 0.09 | 0.9994 | 0.9967 | 1.0022 | 0.97 |
| Lags 0-1 | 0.9993 | 0.9988 | 0.9998 | 0.79 | 0.9910 | 0.9878 | 0.9941 | 0.58 |
| Lags 0-2 | 0.9944 | 0.9938 | 0.9950 | 0.06 | 0.9904 | 0.9868 | 0.9939 | 0.60 |
| Lags 0-3 | 0.9914 | 0.9907 | 0.9920 | < 0.05 | 0.9884 | 0.9844 | 0.9923 | 0.57 |
| Lags 0-4 | 0.9903 | 0.9896 | 0.9910 | < 0.05 | 0.9846 | 0.9803 | 0.9889 | 0.49 |
| 2008 | | | | | | | | |
| Lag 0 | 0.9552 | 0.9547 | 0.9557 | < 0.05 | 1.0107 | 1.0080 | 1.0135 | 0.44 |
| Lags 0-1 | 0.9504 | 0.9498 | 0.9509 | < 0.05 | 1.0070 | 1.0038 | 1.0101 | 0.66 |
| Lags 0-2 | 0.9413 | 0.9407 | 0.9419 | < 0.05 | 1.0062 | 1.0027 | 1.0097 | 0.73 |
| Lags 0-3 | 0.9319 | 0.9312 | 0.9325 | < 0.05 | 1.0026 | 0.9988 | 1.0065 | 0.89 |
| Lags 0-4 | 0.9250 | 0.9243 | 0.9257 | < 0.05 | 1.0005 | 0.9964 | 1.0047 | 0.98 |
| 2009 | | | | | | | | |
| Lag 0 | 1.0319 | 1.0314 | 1.0324 | < 0.05 | 1.0077 | 1.0052 | 1.0101 | 0.54 |
| Lags 0-1 | 1.0353 | 1.0347 | 1.0358 | < 0.05 | 1.0108 | 1.0080 | 1.0137 | 0.45 |
| Lags 0-2 | 1.0344 | 1.0338 | 1.0350 | < 0.05 | 1.0131 | 1.0099 | 1.0162 | 0.42 |
| Lags 0-3 | 1.0346 | 1.0339 | 1.0352 | < 0.05 | 1.0162 | 1.0128 | 1.0197 | 0.36 |
| Lags 0-4 | 1.0355 | 1.0348 | 1.0362 | < 0.05 | 1.0177 | 1.0140 | 1.0215 | 0.35 |
| 2010 | | | | | | | | |
| Lag 0 | 1.0268 | 1.0263 | 1.0273 | < 0.05 | 0.9817 | 0.9791 | 0.9844 | 0.18 |
| Lags 0-1 | 1.0374 | 1.0368 | 1.0379 | < 0.05 | 0.9703 | 0.9674 | 0.9732 | < 0.05 |
| Lags 0-2 | 1.0425 | 1.0419 | 1.0431 | < 0.05 | 0.9654 | 0.9623 | 0.9686 | < 0.05 |
| Lags 0-3 | 1.0442 | 1.0435 | 1.0448 | < 0.05 | 0.9632 | 0.9598 | 0.9667 | < 0.05 |
| Lags 0-4 | 1.0436 | 1.0429 | 1.0443 | < 0.05 | 0.9561 | 0.9524 | 0.9598 | < 0.05 |
| 2011 | | | | | | | | |
| Lag 0 | 1.0092 | 1.0087 | 1.0097 | < 0.05 | 1.0303 | 1.0274 | 1.0332 | < 0.05 |
| Lags 0-1 | 1.0075 | 1.0070 | 1.0081 | < 0.05 | 1.0182 | 1.0149 | 1.0215 | 0.27 |
| Lags 0-2 | 1.0013 | 1.0007 | 1.0019 | 0.68 | 1.0074 | 1.0038 | 1.0111 | 0.69 |
| Lags 0-3 | 0.9960 | 0.9953 | 0.9966 | 0.23 | 1.0092 | 1.0052 | 1.0133 | 0.65 |
| Lags 0-4 | 0.9934 | 0.9927 | 0.9941 | 0.07 | 1.0116 | 1.0072 | 1.0160 | 0.60 |
| 2012 | | | | | | | | |
| Lag 0 | 0.9969 | 0.9964 | 0.9974 | 0.23 | 1.0170 | 1.0142 | 1.0198 | 0.23 |
| Lags 0-1 | 0.9966 | 0.9961 | 0.9972 | 0.23 | 1.0267 | 1.0236 | 1.0299 | 0.09 |
| Lags 0-2 | 0.9914 | 0.9908 | 0.9920 | < 0.05 | 1.0389 | 1.0354 | 1.0423 | < 0.05 |
| Lags 0-3 | 0.9859 | 0.9853 | 0.9866 | < 0.05 | 1.0430 | 1.0393 | 1.0468 | < 0.05 |
| Lags 0-4 | 0.9819 | 0.9813 | 0.9826 | < 0.05 | 1.0450 | 1.0409 | 1.0490 | < 0.05 |
| 2013 | | | | | | | | |
| Lag 0 | 1.0155 | 1.0150 | 1.0160 | < 0.05 | 0.9742 | 0.9713 | 0.9772 | 0.09 |
| Lags 0-1 | 1.0204 | 1.0198 | 1.0209 | < 0.05 | 0.9638 | 0.9604 | 0.9671 | < 0.05 |
| Lags 0-2 | 1.0264 | 1.0258 | 1.0270 | < 0.05 | 0.9661 | 0.9624 | 0.9697 | 0.07 |
| Lags 0-3 | 1.0342 | 1.0336 | 1.0349 | < 0.05 | 0.9654 | 0.9615 | 0.9694 | 0.09 |
| Lags 0-4 | 1.0416 | 1.0409 | 1.0423 | < 0.05 | 0.9676 | 0.9633 | 0.9719 | 0.14 |
| 2014 | | | | | | | | |
| Lag 0 | 0.9768 | 0.9763 | 0.9773 | < 0.05 | 1.0251 | 1.0217 | 1.0285 | 0.14 |
| Lags 0-1 | 0.9775 | 0.9769 | 0.9780 | < 0.05 | 1.0356 | 1.0318 | 1.0394 | 0.06 |
| Lags 0-2 | 0.9756 | 0.9750 | 0.9762 | < 0.05 | 1.0423 | 1.0381 | 1.0464 | < 0.05 |
| Lags 0-3 | 0.9732 | 0.9726 | 0.9738 | < 0.05 | 1.0377 | 1.0332 | 1.0422 | 0.09 |
| Lags 0-4 | 0.9711 | 0.9705 | 0.9718 | < 0.05 | 1.0493 | 1.0445 | 1.0541 | < 0.05 |
| 2015 | | | | | | | | |
| Lag 0 | 1.0257 | 1.0251 | 1.0262 | < 0.05 | 1.0060 | 1.0034 | 1.0086 | 0.65 |
| Lags 0-1 | 1.0262 | 1.0256 | 1.0268 | < 0.05 | 1.0075 | 1.0046 | 1.0105 | 0.62 |
| Lags 0-2 | 1.0248 | 1.0241 | 1.0254 | < 0.05 | 1.0052 | 1.0019 | 1.0085 | 0.76 |
| Lags 0-3 | 1.0246 | 1.0239 | 1.0253 | < 0.05 | 1.0061 | 1.0024 | 1.0098 | 0.75 |
| Lags 0-4 | 1.0277 | 1.0269 | 1.0285 | < 0.05 | 1.0094 | 1.0053 | 1.0135 | 0.65 |
| 2016 | | | | | | | | |
| Lag 0 | 1.0261 | 1.0255 | 1.0267 | < 0.05 | 1.0430 | 1.0388 | 1.0472 | < 0.05 |
| Lags 0-1 | 1.0302 | 1.0295 | 1.0309 | < 0.05 | 1.0267 | 1.0220 | 1.0314 | 0.25 |
| Lags 0-2 | 1.0344 | 1.0336 | 1.0351 | < 0.05 | 1.0262 | 1.0211 | 1.0313 | 0.31 |
| Lags 0-3 | 1.0353 | 1.0345 | 1.0361 | < 0.05 | 1.0282 | 1.0227 | 1.0337 | 0.31 |
| Lags 0-4 | 1.0333 | 1.0324 | 1.0342 | < 0.05 | 1.0328 | 1.0269 | 1.0388 | 0.27 |
